# Analysis of Life Cycle Environmental Impacts of Using Enogen Corn in Beef Cattle Rations

**DOI:** 10.3390/ani11102916

**Published:** 2021-10-09

**Authors:** Marty Matlock, Martin Christie, Greg Thoma

**Affiliations:** 1 University of Arkansas Resiliency Center, University of Arkansas, Fayetteville, AR 72701, USA; mmatlock@uark.edu; 2 Ralph E. Martin Department of Chemical Engineering, University of Arkansas, Fayetteville, AR 72701, USA; martinchristie@protonmail.com

**Keywords:** life cycle assessment, Enogen^®^, beef sustainability, feed conversion efficiency, key performance indicators, global warming potential

## Abstract

**Simple Summary:**

This study presents a comparative lifecycle assessment of the environmental performance of Enogen^®^ corn as an ingredient in beef rations. We show that the reductions in environmental impact per unit of live weight gain are approximately 5% across four primary impact categories: climate change, embedded energy, land use, and water use. These conclusions are robust under a series of evaluations considering different phases of the supply chain.

**Abstract:**

Agricultural production systems have been identified as significant sources of anthropogenic impacts across several environmental key performance indicators (KPIs). Livestock husbandry is growing in global importance as the demand for high-quality protein continues to increase. It is therefore imperative to have sustainable intensification technologies, and we describe one such technology. The purpose of this study was to evaluate the performance of Enogen^®^ corn grain compared to conventional feed corn when used as an ingredient in backgrounding and feed yard beef rations using life cycle assessment. The project was conducted in compliance with ISO standards, including a third-party panel review. A series of scenarios were analyzed to evaluate the impacts of boundaries and functional units on the outcomes. The use of Enogen corn as a feed component in beef production showed a quantifiable benefit in terms of the sustainability metrics of primary interest in this study. The gate-to-gate improvements at the feed yard and backgrounding based on full field trial datasets from field trials conducted at Kansas State University and at the University of Nebraska, Lincoln showed 3.4 and 5.8 percent reductions in Global Warming Potential, respectively. It is particularly noteworthy that the improvement in feed conversion ratio at the feed yard results in approximately 6 percent improvement in the four key environmental performance metrics of beef production, which demonstrates potential for the sector to meet its sustainability targets.

## 1. Introduction

Agricultural production systems have been identified as significant sources of anthropogenic impacts across several environmental key performance indicators (KPIs). Syngenta Seeds developed Enogen brand corn hybrids that have shown improvements in feed conversion ratios in ruminant feeding studies, including beef production studies (Johnson et al., 2018; Jolly-Breithaupt et al., 2019). Enogen corn is a genetically modified product that was designed to produce a heat-stable and pH-tolerant α-amylase enzyme, which improves the digestibility of the starch content of corn and therefore improves the feed conversion ratio (FCR) in beef.

Beef production systems pose higher environmental impacts relative to other livestock sectors due primarily to enteric methane emissions. Enogen corn is an innovative feed ingredient due to the improvements in feed utilization efficiency arising from the endogenous α-amylase, an enzyme that cleaves bonds in starch molecules, improving starch digestion. As a result of this improved feed conversion, more consumable meat can be produced per unit of corn when Enogen corn is included in the feed rations. Livestock systems that require less feed stuffs will implicitly require less fertilizer, water, and land inputs in the upstream supply chain, resulting in a reduction of overall environmental impacts. Additionally, a slightly lower manure production is expected, further compounding potential benefits of the increased FCR. The purpose of this study was to investigate the differences in supply chain impacts of beef fed rations with conventional field corn and Enogen corn, a feed corn modified to express α-amylase in the starchy endosperm of the grain. We find approximately 5% improvement across the main key performance indicators on a per kilogram live weight gain basis for the backgrounding and feed yard stages combined.

## 2. Materials and Methods

Our overarching approach for this assessment was to adapt the reported animal performance and feed consumption from the feeding trials from Kansas State University (KSU) and the University of Nebraska-Lincoln (UNL) to create input files for the Integrated Farm System Model (IFSM). We translated the results of beef feed trials into production simulation model input and used the simulation model output to create a Life Cycle Assessment (LCA) for each scenario. Life Cycle Impact Assessment (LCIA) was used to compare impacts of the two feed ingredients, with Monte Carlo uncertainty analysis used to evaluate significance.

### 2.1. Life Cycle Assessment Method

LCA is used internationally for evaluating the potential environmental impacts of the activities in the life cycle of a good or service. The LCA methodologies used in this assessment are defined by the International Organization for Standardization (ISO) 14040–14044 standards [1,2]. We used the OpenLCA (oLCA) computational platform [3] and Ecoinvent database for the life cycle inventory [4]. OpenLCA is used to link the individual stages of production into a supply chain model. The cumulative materials and energy inputs required to make the product of interest are converted to equivalencies that are representative of environmental burdens; for example, multiple greenhouse gas emissions are converted to carbon dioxide equivalents.

#### 2.1.1. Goal and Scope

The goal of this project was to quantify the differences in life cycle impacts of beef fed rations containing conventional field corn versus Enogen corn. The objectives of this analysis included:

- Characterize the sources of environmental impacts associated with Enogen versus conventional field corn.
- Quantify the relative impacts of production systems provided by Enogen corn rations versus conventional field corn rations based on edible beef cuts and live weight gain.
- Evaluate the robustness of conclusions through uncertainty assessment and scenario analyses.

#### 2.1.2. Life Cycle Impact Assessment

The intention of this comparative LCA was to determine if the substitution of a conventional corn (CNV) with Enogen corn (EFC) provides environmental benefits in the beef supply chain. The scope of this comparative LCA was cradle to harvest gate since there is no differential performance due to the feed rations after animal harvest. We used the ReCiPe impact assessment framework with the Hierarchist (H) cultural perspective [5,6]. Key Performance Indicators (KPIs) of environmental impacts included:

1. Total Live Weight Gain (LWG), kg liveweight
2. Average Daily Gain (ADG), kg liveweight
3. Gain to Feed Ratio (G:F)
4. Climate Change Potential, kg CO_2 eq._
5. Land Occupation (Land), m^2^ a (occupation of 1 square meter for 1 year)
6. Water Consumption (Water), m^3^
7. Fossil Energy (Energy), kg oil eq.

The assessment of KPIs based on live weight gain (LWG) is reported on a gate-to-gate basis for backgrounding, feed yard, and backgrounding plus feed yard. Inputs that were estimated to impact the performance characteristics and environmental impacts by less than two percent (cumulatively) were included if readily available but were not given priority for additional data collection.

#### 2.1.3. Functional Unit for LCA

The Life Cycle Assessment methodology requires the definition of a functional unit, a common basis for comparing multiple systems. The complete suite of analyses in this study evaluated several sub-systems and functional units:

1. 1000 kg liveweight gained—for gate-to-gate systems of backgrounding and feed yard.
2. 1000 kg of retail cuts.

Each functional unit was analyzed for the performance characteristics (KPI 1–3 above) and environmental impacts (KPI 4–7 above).

#### 2.1.4. System Boundary Description

**Figure 1:**
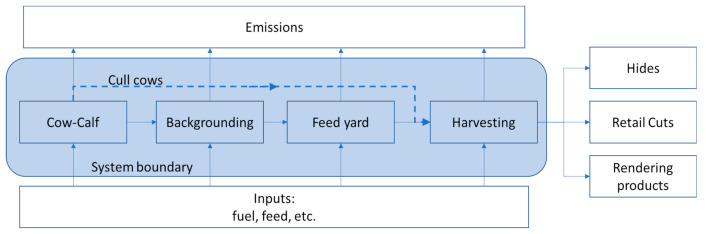
Beef lifecycle stages. Note that this is not representative of all potential pathways; sometimes weaned calves leave the cow/calf stage directly to feed yards.

LCA requires the definition of the physical boundaries at which material and energy flows can be considered inputs or outputs to the supply chain. Beef cattle production is comprised of multiple steps where animals are fed until they are large enough for the next production stage (Figure 1). The process starts at the cow/calf operation where calves are born and stay with their mothers while raised on pastureland. Once the calves reach an acceptable weaning weight, they move to backgrounding or stocker operations, where a higher proportion of energy dense feedstuff is introduced. The function of the backgrounding operation is growing animals that become feeders who enter the feed yard. The feed yard is the final live stage of production prior to harvesting. Cattle are provided grain rich diets for rapid weight gain. Cattle are then sent to the harvesting facility where edible cuts of beef are produced, along with rendering co-products and hides.

In the base case for this study, we assumed that calves were weaned at 244.5 kg, then entered the backgrounding stage where they were fed to a weight of 293 kg, and then they were transferred to a feed yard and raised to 570 kg (CNV) or 590 kg (EFC). This study includes analyses of the supply chain impacts for three distinct system boundaries, including cradle to harvesting gate (quantifying full system, impacts of producing 1000 kg of retail cuts, Figure 1). The full lifecycle provides a picture of how differences in feed corn characteristics influence the environmental burden of a system representative of actual production. Edible cuts were selected as a functional unit to present the results relevant to the downstream supply chain.

We also evaluated the backgrounding gate to feed yard gate (quantifying the impacts of 1000 kg liveweight gained across the two feeding phases receiving EFC) and feed yard gate to harvesting facility receiving gate (quantifying the gate-to-gate impacts of 1000 kg of live weight gain).

#### 2.1.5. Reference Flows and System Description

The reference flows for this project were organized by systems to support the cradle to harvest gate boundaries. The reference flows, which were necessary to provide the specific functional unit for the analysis, were:

1. Cow/calf: Calves to backgrounding and culled cows to harvesting as outputs;
2. Backgrounding: Growing animals as inputs to finishing operations;
3. Finishing: Live animal body weight as an input to harvesting;
4. Animal model outputs are all inputs to the harvesting process;
5. Edible meat, rendering products, and hides are the outputs of the harvesting process.

Upstream processes for purchased inputs (e.g., fuel, electricity, transportation, etc.) were taken, unmodified, from the Ecoinvent v3.4 cut-off database. We chose the average market process for US electricity. Each of the reference flows is described below.

##### Cow/Calf Operation

The life cycle of beef cattle begins with calves born and kept with their grazing mothers on pastureland. Calves are weaned and removed from their mothers between 180–210 days (modeled at 244.5 kg to match the Kansas background feeding trial starting weight). Concentrate rations are generally not provided at this stage of production, so there will not be direct improvements associated with EFC. However, there will be upstream benefits that arise due to improved feed efficiency at backgrounding and the feed yard; systems with more efficient backgrounders and finishers will require fewer calves to produce the same mass of edible beef. We included the cow/calf operation in the retail cut scenarios to quantify these benefits. This is primarily a consequence of the observation in the Nebraska feed yard trials, in which there was an interaction effect between improved feed conversion and live weight gain for the EFC treatment.

##### Backgrounding

Backgrounding is an intermediate production phase prior to placement at the feed yard [7]. During this stage, animals are provided with feed concentrates in addition to forage; the Kansas backgrounding study included an evaluation of CNV and EFC in the ration. It should be noted that not all calves proceed from the cow/calf to the backgrounding phases. Some proceed directly from the cow/calf operation to the finishing stage; however, this potential supply chain option is not included in the evaluation reported here.

##### Finishing

The primary goal of this stage is to maximize healthy weight gain by providing rations with a higher ratio of concentrates to forage. The inclusion of EFC during this stage was shown through field trials to result in improved gain to feed ratios, and in turn, lower emissions, manure production, and upstream input requirements.

##### Harvesting

Finished cattle leaving the feed yard are received by the harvesting facilities for processing into edible cuts of beef. The boundary for the study system is the packaged, edible cuts leaving the loading dock. Transportation, processing, or consumption downstream of the harvesting facility is not considered in this analysis. There will be no direct benefit of EFC at the harvesting stage.

##### Feed Production

Corn silage, alfalfa silage, and corn grain production were simulated using the “crop farming” module of IFSM. Briefly, single crop simulations for each of the feed ingredients were performed for production in the same region that beef farms were simulated. The IFSM simulates crop growth based on planting dates, simulated weather, and a full complement of field operations from field preparations through harvest, including fertilization and application of crop protection chemicals. The inventory generated from these simulations was transferred to the OpenLCA platform and linked to the Ecoinvent database.

#### 2.1.6. Allocation Procedures

Many processes produce multiple products and are known as multi-functional processes. Assignment of environmental impacts to co-products is a key methodological decision point in an LCA study. There are relatively few processes in this system that require allocation. These include distiller’s grains from ethanol production, soybean meal, and beef byproducts from harvesting. In this system, cull cows from cow/calf operation could be considered a co-product; however, in our modeling we have avoided this allocation step by assigning all the burden to the calves sent to backgrounding operations. The culled cows are then reincorporated to the input inventory at the harvesting stage, without additional burden. This is mathematically equivalent to assigning an allocation factor at the cow/calf operation and then recombining it at the harvest facility receiving dock. Cull cows are removed from the cow/calf herd for slaughter, but do not include animals that die naturally or from illness, which are managed as a waste stream.

#### 2.1.7. Temporal, Geographic, and Technological Boundaries

The geographic region included the central and intermountain US production regions for cow/calf operations and Kansas and Nebraska feeding trial locations based on work by Rotz et al. [8]. We adapted IFSM farm files for these regions to simulate the beef production phases of the supply chain for this study. The technological boundaries were modern production practices across each of the categories of analyses.

#### 2.1.8. Precision, Completeness, and Representativeness of Data

The effects under investigation in this study are intended to quantify the sustainability metrics associated with different feed ingredients to produce finished beef. Because the impact assessment is focused on a subset of the available midpoint categories from the ReCiPe 2016 (H) impact assessment framework, we did not emphasize collection of data regarding pesticide use in the beef sector and included generic pesticides for crop production. There are no substantive omissions of input information relevant to the main impact categories. The experimental trials that form the basis of the lifecycle inventory data are expected to be representative of backgrounding and feed yard operations in the upper Midwest.

#### 2.1.9. Data Quality

All the data points were curated through a data quality criteria process that included assigned quality rankings based upon variability reported in the studies and based on variation in the multi-year simulations of IFSM. Many of the input variables and emissions used in the IFSM simulations had coefficients of variation that were artificially small due to the number of iterations analyzed. In those cases, based on our expert judgment, we adjusted the coefficient of variation to 5% for inputs and 10% for emissions for purposes of a Monte Carlo simulation.

#### 2.1.10. Cut-off Criteria

We adopted the Food and Agriculture Organization of the United Nations LEAP (Livestock Environmental Assessment and Performance) guidelines regarding cutoff data [9]. We did not include items such as veterinary services for health management, artificial insemination, or supporting services such as nutrition or accounting services.

### 2.2. Sensitivity Analysis for Refining the System Boundary

We identified a suite of system boundary scenarios to highlight the expected contributions to sustainability performance of each beef production stage, individually and as components of a full system. We considered the sensitivity of the results to modeling choices regarding the approach to account for the overlapping weight ranges between the KSU background trial and UNL feed yard trial. This is intended to address an ISO requirement regarding refinement of system boundaries to ensure important inputs/impacts are not missed. Regarding sensitivity of the results to cut-off processes, we have identified the excluded activities previously, which are uniform across all scenarios. Further, no known significant activities were excluded, and thus we did not perform this sensitivity evaluation.

### 2.3. Scenario Development

Because of the mismatch between the backgrounding trial ending weight and the feed yard trial starting weight in the published studies, we performed alternative calculation models to assess the robustness of conclusions. We included both gate-to-gate evaluation of backgrounding (BG) and feed yard (FY) studies. The rationale behind selection of multiple scenarios (Table 1) was to evaluate alternate sets of assumptions to account for the discontinuities of animal weights in the experimental data. For Scenarios 5 and 6, we have assumed that the animal performance simulated by IFSM is representative of conditions that deviate from the calibration conditions. In Scenario 5, we assumed that the weight gain is the primary determining factor for resource use and emissions because the inputs and emissions and LWG modeled for the feed yard were held constant, although the final liveweight produced was larger. This accommodated larger backgrounders entering the feed yard (to match the KSU backgrounding ending weight). This will necessarily lead to an underestimation of the emissions on a liveweight basis because larger animals will have higher maintenance requirements that are ignored. In Scenario 6, we simulate the larger animal entering the feed yard using the calibrated IFSM input files, except for changing the starting and finishing weights. In this instance, the IFSM simulated ration is changed from the UNL report, despite identical feed nutrition characteristics and maximum inclusion rates. This is expected to be more representative of animal performance at the feed yard for the situation with larger backgrounders entering.

### 2.4. Life Cycle Inventory

The life cycle inventory was constructed from a combination of data from public databases such as USDA-ARS and NASS, peer-reviewed scientific data, specific feed trials sponsored by Syngenta and published by Kansas State University and The University of Nebraska, and simulated processes using IFSM. Unit processes were constructed in Microsoft Excel and transferred into the OpenLCA platform for lifecycle impact assessment.

#### 2.4.1. Cow/Calf Operation

The same cow/calf unit process was used in each of the simulations, so there is no direct performance difference arising from this phase in production. However, the improved gain to feed ratio observed for cattle fed EFC results in a reduction of the upstream requirements to produce a unit of edible meat than their conventional corn-fed counterparts.

The cow/calf unit process produces weaned calves and cull cows. The reference flow for weaned calves stays within the system boundary. The cull cow reference flow bypasses intermediate stages as described in Section 2.1.6.

#### 2.4.2. Backgrounding

Backgrounding inventory data were derived from calibrated IFSM simulations of the feeding trial conducted by Kansas State University [10]. The purpose of this calibration is to establish a representative simulation to provide inventory data for processes not measured in the trials (e.g., enteric methane and manure management). For purposes of this assessment, the dry-rolled and whole corn treatments were pooled, leaving two ration compositions as the experimental treatments, Enogen corn (EFC) and conventional corn (CNV). Two IFSM farm files were developed to represent backgrounding operations of the study, one for CNV treatment and one for EFC treatment. Supplementary Material provides complete animal performance data used for the backgrounding calibration as well as the calibrated IFSM outputs.

#### 2.4.3. Finishing

As with the backgrounding process, IFSM was used to generate inventory matched to the UNL experimental treatment information. Simulated animal performance and ration composition/quantity consumed were calibrated to UNL trial results. The Supplementary Material provides complete animal performance data used for the feed yard calibration as well as the calibrated IFSM outputs.

#### 2.4.4. Harvesting

A representative, average harvesting process was used. Proprietary data from several harvesting facilities, collected and aggregated under a previous project, were used to calculate the harvesting facility emissions. Because this model was constructed with an input of carcass weight, rather than liveweight, we added an intermediate unit process that combined culled cows with a dressing percentage of 50% and finished animals with a dressing percentage of 63% into a carcass input process for harvesting [8].

#### 2.4.5. Feed Trial Data

Feed trial data from two independent investigations were used to calibrate systems modeling responses for this LCA. The two trials included a feed efficiency assessment in calf diets conducted by Kansas State University [10] and an assessment on finishing cattle performance and carcass characteristics conducted by the University of Nebraska, Lincoln [11].

### 2.5. The Integrated Farm System Model

The IFSM has been developed over the past 40 years to provide production decision support for US beef producers [12]. The model integrates the physical and biological processes on beef production facilities at the farm level, making it an appropriate scale for simulating production practices. IFSM simulated crop production, feed use, nutrient cycling with land application of manure, and animal responses to nutritive value of available feeds. Rotz et al. applied IFSM to an LCA of environmental footprints of beef production in Kansas, Oklahoma, and Texas [13]. Rotz et al. [8] analyzed the US beef environmental footprint for GWP, energy use, water consumption, and nitrogen loss using IFSM to simulate regional scenarios [12].

Our simulations were based on a KSU backgrounding trial and a UNL finishing trial, in which beef animals were fed dry-rolled Enogen corn and were compared with animals fed conventional field corn as part of the complete ration [10,11]. The IFSM output data were formatted to match Open LCA data structure requirements (extracted from text files, transferred to Excel, and then imported into Open LCA) and used to populate unit processes with lifecycle inventory inputs and emissions. We augmented the IFSM output to include indirect nitrous oxide using the IPCC factors based on the IFSM reported ammonia emissions.

#### 2.5.1. IFSM Calibration Methodology

A calibration procedure was used to match the ration composition, mass of ration consumed, and final herd weights for each treatment in each respective study. These variables included land area for crop production, duration of the finishing period and body weight targets. The IFSM model does not support direct specification of the animal ration, since it is a whole farm simulation tool that simulates the availability of self-grown rations. The IFSM input variables, which were adjusted to match the simulated results with observed results, include the number of finishing cattle, the feeding limit in kg dry matter per animal per day of each ingredient (excluding grain silage), and the net energy of maintenance (NEm) of grain silage. We modified the nutrition and digestibility characteristics of some of the ration ingredients to fine tune the IFSM-reported ration, to match the reported feeding trial ration. The number of cattle in each treatment of the UNL trial was 60 head. However, IFSM calculates the liveweight of the herd as an output, rather than the weight gained per animal. Therefore, the herd size can be adjusted by one or two head to decrease the difference between observed and simulated final liveweights of the herd. Additionally, choosing mass as a functional unit in the LCA software eliminates the need to exactly match the herd size considering the total liveweight of the herd and total feed consumed, associated with the simulated inventory matches the UNL study.

The inclusion limit sets the maximum quantity of a ration ingredient that can be provided to a single animal in a single day. This parameter was used to match the simulated quantity of dry grain (corn), crude protein supplement (urea), and rumen undegradable protein (distiller’s grain) to the reported consumption rates in the UNL study. A manual, iterative approach was used to match the simulated inclusion of each ingredient to its respective composition in the UNL study. The inclusion limit of individual ingredients was adjusted up or down to increase or decrease the mass of the ingredient until the total simulated consumption matched the UNL trial. This was performed for each ingredient, excluding grain silage, until the ration composition and quantity matched that in the UNL trial within one percent.

The total NE_m_ of the ration determines if the energy required for maintenance is met and if additional caloric energy is available for weight gain. There is an inverse relationship between NE_m_ and inclusion rate, meaning that a more energetically dense (higher NE_m_) silage will result in a lower dry matter intake to meet the energy requirement. This allowed the adjustment of corn silage inclusion until the simulated values matched those in the UNL trial. Based on the IFSM documentation [12], there should be no unreasonable effects of adjusting inclusion rates via NE_m_.

#### 2.5.2. Key Assumptions

We analyzed the systems to determine the impacts (if any) of an improved gain to feed ratio. We assumed the management practices used to produce animals are the same at each step, except for different feed ration components (CNV versus EFC). The IFSM was assumed to consistently simulate beef production systems across the two feed components.

### 2.6. Uncertainty Analysis

We conducted pairwise Monte Carlo simulations for each of the scenarios described above to assess the level of confidence that inclusion of EFC corn leads to reduction in environmental impacts [14]. The Monte Carlo runs were conducted sequentially using a random variate from the probability density function for each of the input flows for which a distribution has been defined. The individual results of these randomly selected inputs are then compiled to provide a probability distribution function for the output, in this case the impact categories. Subsequently, statistical tests can be performed to determine if the results from two systems delivering the same functional unit are different from each other or not.

We constructed product systems in the OpenLCA (oLCA) software platform that had reference flows of +1000 kg and −1000 kg of CNV treatment and EFC treatment, respectively. Each pair (CNV—EFC) was simulated 250 times using the Monte Carlo impact assessment tool in oLCA. We then performed a bootstrap evaluation for each pair [15,16,17,18]. Briefly, 30 samples were randomly selected, with replacement, from the 250 MCS runs, and a one-sided t-test was performed on the paired differences to determine the probability of rejecting the null hypothesis that the mean(CNV) = mean(EFC) in favor of the alternate hypothesis that the mean(CNV) > mean(EFC). The bootstrap selection of 30 samples was repeated 300 times, generating a distribution of *p*-values. We then calculated the 99% confidence interval for this distribution and rejected the null hypothesis only when the 99th percentile of *p*-values was less than 0.01.

## 3. Results

### 3.1. Scenarios 1 and 2: Calibration and Backgrounding and Finishing: Gate-to-Gate

Our first set of system boundary scenarios is a gate-to-gate evaluation of the two phases most directly affected by the selection of EFC, the backgrounding and feed yard stages. And in this section, we consider the separate systems using a consistent set of assumptions that are also used in the retail cut evaluation to provide the functional units of 1000 kg LWG and 1000 kg of retail cut, which are provided in the Supplemental Material.

#### 3.1.1. Scenario 1: Feed Yard Trial Calibration and Comparison

The unmodified Nebraska feed yard trial analyses showed a consistent reduction across all four KPIs (Table 2). These results are expectedly due to a combination of improved feed conversion and enhanced weight gain during the finishing period. The calibration procedure is described above.

#### 3.1.2. Scenario 2: Backgrounding Trial Calibration and Comparison

Results for the KSU trial analyses were similar to UNL (Table 3). Over the full 92-day trial, there were notable benefits in each of the impact categories. Because ending weights were similar for the two treatments, the improvement is largely attributable to the increased digestibility of the EFC and subsequent FCR increase.

### 3.2. Scenarios 3 and 4: Truncated Background and Matched Systems

#### 3.2.1. Scenario 3: Truncated Backgrounding

Comparative results for Scenario 3 are not presented as standalone results but are incorporated into Scenario 4 and Scenario 6.

#### 3.2.2. Scenario 4: Matched Systems; Gate-to-Gate and Cradle-to-Harvest Gate

There are two primary system boundary combinations under consideration in this scenario: the combined feeding stages (BG plus FY) presented with a functional unit of 1000 kg of live weight gain, and a full cradle-to-harvest gate assessment that includes both cow/calf and a harvesting unit process with a functional unit of 1000 kg of retail cut beef. The differences as percentage improvements from EFC in the combined (truncated) background and feed yard stages (gate-to-gate) showed greater than five percent for all KPIs (Table 4). The driver for the improvement shown in this table is expected to be principally from the feed yard, as the truncated background has a relatively small benefit due to the limited number of days on feed needed to reach the feeder starting weight reported in the UNL trial. The percentage improvement associated with adoption of EFC in the context of a full production and processing supply chain (cradle to harvest gate for the functional unit of 1000 kg of retail cuts) were smaller than the gate-to-gate improvements. This is because there are only minor effects of the backgrounding feed yard stages (Table 5) on the up- and down-stream impacts, thus the addition of emissions from the cow/calf and harvesting stages, in essence, dilutes the benefit from the EFC feeding stages.

**Figure 2:**
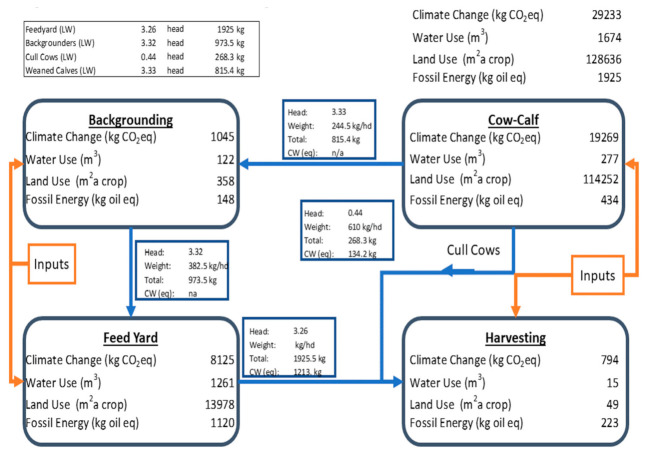
Base case for 1000 kg of retail cuts. Truncated BG; Full FY; Enogen Corn.

**Figure 3:**
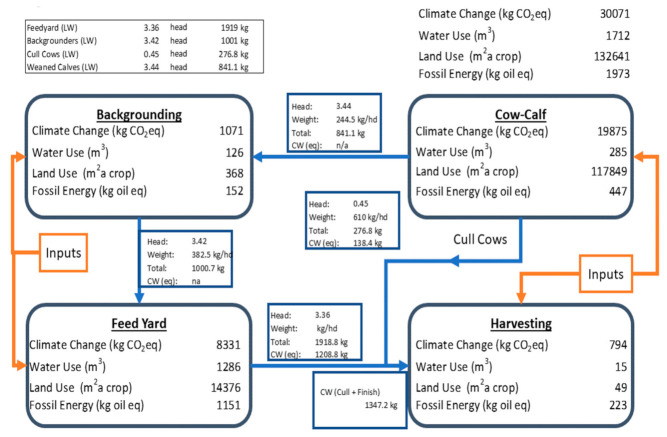
Base case for 1000 kg of retail cuts. Truncated BG; Full FY; Conventional Corn.

We present impact results and reference flows in Figure 2 and Figure 3 for the cradle-to-harvest gate system producing 1000 kg of retail cut beef for the EFC treatment and the CNV treatment, respectively. These diagrams show each production stage’s contribution to the overall impact (tabulated in the upper right corner of the diagram). In addition, the movement and weight of animals is presented. These diagrams represent the post-harvest allocation results, and therefore all the reported impacts at each stage are associated to the functional unit of 1000 kg retail cut products. Because we used a revenue-based allocation, the dressing percentages estimated from the animal weight in the figure will not match the actual dressing percentages used in the modeling.

Note that the same cow/calf unit process was used in each of the simulations, so there is no direct performance difference arising from this phase in production. However, the improved gain to feed ratio, coupled with the improved weight gain observed in cattle fed EFC, results in reduced upstream requirements to produce a unit of edible meat compared to the animals fed with the conventional corn ration.

### 3.3. Scenario 5 and 6: Paired and Matched Live Weight Gain

#### 3.3.1. Scenario 5: Paired LWG

This scenario is a simulation intended to test the removal of the interaction effect of improved feed conversion and increased weight gain observed in the UNL trial (Table 6). In summary, we used a modified version of both the background and feed yard trial simulations, in which the modifications imposed were related to the starting and ending weights of the animals. The background trial started with the same weaned calf weight and produced feeders weighing 380 kg, for both feed treatments. The feed yard simulation was modified to start with animals weighing 380 kg and finished them at 662 kg, again maintaining the same starting and ending weight for both treatments. Thus, the simulated feed conversion for the feed yard stage was lower than observed in the UNL trial, because larger animals are somewhat less efficient at feed conversion.

#### 3.3.2. Scenario 6: Matched LWG

An alternate perspective to Scenario 4 is presented in Table 7. This scenario was constructed because Scenario 4 does not capture all the benefits of using EFC in the backgrounding stage because animals in both treatments track very similarly until about 60 days into the trial, when the EFC treatment begins showing improved performance. Scenario 6 was constructed to simulate the feed yard trial with the modification that the feeder starting weight was set to the finishing weight from the backgrounding phase. The remaining feed yard calibration parameters were not adjusted in the simulation. Thus, the feed yard ration characteristics and inclusion rates were held constant, as was the target live weight gain for each treatment; however, the ration simulated by the IFSM no longer matched the Nebraska trial data. The simulated ration included significantly more corn silage, and the animals consumed more total feed—as expected given their larger body weight and therefore larger maintenance energy requirements. Nonetheless, this simulation suggests that use of EFC for heavier animals will result in similar improvements, lending additional support to the overall conclusions regarding the potential benefits of Enogen corn.

### 3.4. Uncertainty Analysis

The Monte Carlo simulations showed that, for most of the scenarios and impact categories, the CNV treatment had a higher impact than the EFC treatment. Based on the bootstrap approach, we can assert that we have 99% confidence that the *p*-value for the pairwise comparison is less than 0.01, indicating 99% confidence that the EFC treatment leads to reduction in impact. Only the truncated background feed yard and Scenario 5 failed to reject the null hypothesis that the conventional and EFC treatments are equivalent for all the selected KPI’s (Table 8). In the backgrounding trial, the treatments did not begin to significantly separate until approximately 60 days into the trial, and the truncated simulation only included the first 25 days. In Scenario 5, the benefit of improved feed conversion is captured; however, as described in the methodology section, the interaction effect observed in the trial between feed conversion and cumulative weight gain is not captured in this scenario, thus having some impacts that are not statistically different was expected.

## 4. Discussion

The experimental trial data available from KSU (backgrounding) and UNL (feed yard) were independently conducted and not coordinated. Thus, as mentioned, scenarios were constructed to evaluate the robustness of conclusions subject to alternate modeling approaches to account for these differences. The two calibration scenarios (1 and 2) show between 3.5 and 5% improvement across the four key performance indicators demonstrating the efficacy of EFC and its potential for reducing environmental impacts from beef production. The impacts across the two stages are not fully additive because of the mismatch in finishing and starting weights between the BY and FY stages in the feeding trials. The alternate modeling presented in scenarios 5 and 6 have the same directional results, although, due to simulating larger animals, the magnitude of improvement is somewhat reduced.

These results support the hypothesis that the application of EFC in beef rations leads to overall environmental benefits mediated by improved feed conversion associated with improved digestibility of EFC and the consequent reductions in the upstream inputs for the production.

### 4.1. Data Quality

The lifecycle inventory data used in this assessment is derived directly from the two feed trials conducted at Kansas State University and at the University of Nebraska, and we believe these data are of high quality given the number of replications and statistical analysis provided by the authors. The Integrated Farm System Model is a well-established tool for simulating beef production systems, and the rigorous calibration procedure that was followed ensures just as good of data quality for the additional inventory metrics used in the OpenLCA platform. Specifically, the estimations of emissions associated with enteric methane and manure management are expected to be robust based on the wide acceptance and use of IFSM in simulating beef production.

### 4.2. Limitations

The study’s conclusions are robust under the assumptions and limitations imposed by the availability of experimental data. For the backgrounding study, we used pooled data combining dry rolled with whole corn treatments, and thus the generic substitution of Enogen Corn for conventional corn will provide the environmental benefits, but this study does not differentiate between the substitution of whole corn and dry rolled corn.

The Nebraska feeding trial had two ration treatments, one based on Sweet Bran and a second based on wet distillers’ grains as an additive to the dry rolled corn. This evaluation only considered the distiller’s grain-based ration, and thus the conclusions should be applied cautiously to substitutions in other situations.

## 5. Conclusions

The purpose of this analysis was to evaluate the differences in beef production efficiency and environmental impacts using conventional corn and Enogen Corn as feed components. Enogen corn provides a quantifiable benefit (approximately 5% improvement in the impact metrics at both backgrounding and feed yard on a live weight gained basis) in terms of the sustainability metrics of primary interests in this study. These conclusions are robust under a series of evaluations considering alternate system boundaries and functional units. The fractional improvement across the full supply chain is, of course, lower than the benefit observed for the production stages where the Enogen corn is directly utilized. This is essentially a dilution effect, in which the larger overall footprint causes the improvement at the backgrounding and feed yard stage to represent a smaller fraction of the total.

In the context of overall improvement in the supply-chain sustainability, this technology provides quantifiable improvements. Continual improvement is needed as the global population continues to grow and the number of affluent, middle-class people demanding high-quality nutritious protein is burgeoning. Technologies such as Enogen corn are important contributing factors in support of more sustainable and secure food supplies.

## Figures and Tables

**Table 1:** Scenarios analyzed in the LCA comparing conventional corn and Enogen corn feed component efficiencies and impacts on beef.

				Functional Unit	
Scenario	Calibrated IFSM Files	IFSM Parameters	Rationale for Scenario	1000 kg Retail Cuts *	1000 kg LWG (BG and/or FY)
1: UNL Calibration	--	UNL start weight to finish weight as reported	Calibration	N. A.	Yes
2: KSU Calibration	--	244.5 kg starting weight up to KSU end weight	Calibration	N. A.	Yes
3: KSU Truncated Calibration	--	244.5 kg to UNL start weight using ~25 days of interpolated KSU data	Calibration.	N. A.	Yes
4: Matched ** systems	Truncated BG: Scenario 3 Full FY: Scenario 1	Truncated BG: Scenario 3 data. Full FY: Scenario 1 data.	Combined stages most relevant for EFC benefits.	Yes	Yes.
5: Paired LWG	Full BG: modified Full FY: modified	Background end weight = 380 kg for both feed treatments. Feed yard gain from 380 kg to 662 kg for both feed treatments.	Testing intended to eliminate the observed interaction between feed conversion ratio and weight gain.	Yes	Yes
6: Matched LWG	Full BG: Scenario 2 Full FY: Modified	BG Scenario 2 model used. Modified FY: starting weight chosen as background ending weight and live weight gain maintained at UNL trial values.	Combination of full, calibrated KSU simulation with FY with feeder weights based on KSU end weights.	Yes. Intended to capture the full backgrounding (92 days on feed) plus the interaction of FCR and weight at FY.	Yes

* For the retail cut functional unit, the background (BG) and feed yard (FY) stages were coupled with generic cow/calf and harvesting unit operations. ** Matched (#4 &#6) means simulations matched to observed feeding trial; paired means both treatments were simulated with the same LWG (Total Live Weight Gain).

**Table 2:** Scenario 1: Environmental impacts and improvements for full gate-to-gate UNL feed yard trial.

Impact Category	Units	Conventional	Enogen	Enogen^®^ Percent Decrease in Impact
Climate change	(kg CO_2_eq/1000 kg LWG)	8608 ^a^	8109 ^b^	−5.80%
Land use	(m^2^ a/1000 kg LWG)	15,405 ^a^	14,461 ^b^	−6.13%
Water use	(m^3^/1000 kg LWG)	1384 ^a^	1307 ^b^	−5.61%
Fossil energy	(kg oil eq/1000 kg LWG)	1127 ^a^	1060 ^b^	−5.99%

Values with different letters (^a, b^) within a category (row) are significantly different (*p* < 0.01). LWG: Total Live Weight Gain.

**Table 3:** Scenario 2: Environmental impacts and improvements for full gate-to-gate KSU backgrounding trial.

Impact Category	Units	Conventional	Enogen	Enogen^®^ Percent Decrease in Impact
Climate change	(kg CO_2_eq/1000 kg LWG)	6954 ^a^	6719 ^b^	−3.39%
Land use	(m^2^ a/1000 kg LWG)	3365 ^a^	3191 ^b^	−5.17%
Water use	(m^3^/1000 kg LWG)	1147 ^a^	1088 ^b^	−5.13%
Fossil energy	(kg oil eq/1000 kg LWG)	577 ^a^	557 ^b^	−3.53%

Values with different letters (^a, b^) within a category (row) are significantly different (*p* < 0.01). LWG: Total Live Weight Gain.

**Table 4:** Environmental impacts and improvements for gate-to-gate backgrounding plus feed yard (truncated BG; full FY).

Impact Category	Units	Conventional	Enogen	Enogen^®^ Percent Decrease in Impact
Climate change	(kg CO_2_eq/1000 kg LWG)	8546 ^a^	8076 ^b^	−5.49%
Land use	(m^2^ a/1000 kg LWG)	13,414 ^a^	12,638 ^b^	−5.78%
Water use	(m^3^/1000 kg LWG)	1284 ^a^	1218 ^a,^*	−5.10%
Fossil energy	(kg oil eq/1000 kg LWG)	1184 ^a^	1117 ^b^	−5.72%

Values with different letters (^a, b^) within a category are significantly different (*p* < 0.01). * *p* < 0.03 for water use, thus there is 97% rather than 99% confidence that the treatments are different. LWG: Total Live Weight Gain.

**Table 5:** Environmental impacts and improvements for cradle-to-harvest gate. Simulation with truncated BG and full FY conditions plus generic cow/calf and harvest facility models.

Impact Category	Units	Conventional	Enogen	Enogen^®^ Percent Decrease in Impact
Climate change	(kg CO_2_eq/1000 kg retail cut)	30,071 ^a^	29,233 ^b^	−2.79%
Land use	(m^2^ a/1000 kg retail cut)	132,641 ^a^	128,636 ^b^	−3.02%
Water use	(m^3^/1000 kg retail cut)	1712 ^a^	1674 ^b^	−2.21%
Fossil energy	(kg oil eq/1000 kg retail cut)	1973 ^a^	1925 ^b^	−2.48%

Values with different letters (^a, b^) within a category (row) are significantly different (*p* < 0.01).

**Table 6:** Environmental impacts and improvements for Scenario 5, full BG plus full FY. Animals simulated for both feed treatments to reach the same background and finish weights; FY gain from 380 to 662 kg.

Impact Category	Units	Conventional	Enogen	Enogen^®^ Percent Decrease in Impact
Climate change	(kg CO_2_eq/1000 kg retail cut)	8286 ^a^	8131 ^a^	−1.87%
Land use	(m^2^ a/1000 kg retail cut)	11,442 ^a^	11,269 ^b^	−1.51%
Water use	(m^3^/1000 kg retail cut)	1310 ^a^	1264 ^b^	−3.52%
Fossil energy	(kg oil eq/1000 kg retail cut)	1021 ^a^	1010 ^a^	−1.13%

Values with different letters (^a, b^) within a category (row) are significantly different (*p* < 0.01).

**Table 7:** Scenario 6: Environmental impacts and improvements for gate-to-gate UNL feed yard trial. IFSM modified the simulated larger animals with the same live weight gain as the UNL trial.

Impact Category	Units	Conventional	Enogen	Enogen^®^ Percent Decrease in Impact
Climate change	(kg CO_2_eq/1000 kg LWG)	9301 ^a^	8844 ^b^	−4.92%
Land use	(m^2^ a/1000 kg LWG)	15,797 ^a^	14,910 ^b^	−5.61%
Water use	(m^3^/1000 kg LWG)	1490 ^a^	1432 ^a^	−3.85%
Fossil energy	(kg oil eq/1000 kg LWG)	1266 ^a^	1194 ^b^	−5.68%

Values with different letters (^a, b^) within a category are significantly different (*p* < 0.01). LWG: Total Live Weight Gain.

**Table 8:** Heatmap of Statistical Evaluation of Bootstrap Monte Carlo Results.

System Boundary	Scenario	Global Warming	Water Consumption	Land Use	Fossil Resource Scarcity
UNL calibration	1	2.3 × 10^−8^	6.6 × 10^−5^	2.1 × 10^−18^	7.5 × 10^−9^
KSU calibration	2	4.8 × 10^−3^	5.9 × 10^−6^	6.6 × 10^−7^	4.9 × 10^−5^
BGFY (GtG ^§^)	4	4.1 × 10^−4^	2.7 × 10^−2^	8.8 × 10^−4^	4.0 × 10^−4^
Cradle-to-FY gate	4	0.0	4.2 × 10^−4^	0.0	0.0
BGFY (GtG ^§^)	5	9.8 × 10^−3^	6.2 × 10^−4^	2.3 × 10^−4^	7.8 × 10^−2^
Retail cut	5	1.4 × 10^−1^	1.2 × 10^−3^	1.5 × 10^−3^	6.6 × 10^−2^
BGFY (GtG ^§^)	6	5.6 × 10^−8^	2.2 × 10^−5^	2.1 × 10^−15^	3.0 × 10^−7^
Retail cut	6	0.0	3.3 × 10^−9^	0.0	0.0
FY (GtG ^§^)	6	7.4 × 10^−6^	3.1 × 10^−3^	1.4 × 10^−15^	4.1 × 10^−9^

The data reported in this table are the 99 percent confidence limit of the one-sided bootstrap-calculated *p*-values for the rejection of the null hypothesis that CNV = EFC, in favor of the alternative hypothesis that CNV > EFC. Darker green indicates *p* < 10^−5^ (very high confidence), light green indicates *p* < 0.01 (high confidence) and salmon color represents cases for which the null hypothesis is not rejected at *p*-value < 0.01. ^§^ Gate to Gate.

## Data Availability

Supporting data have been provided in the Supplementary Material.

## Supplementary Materials

The following are available online at www.mdpi.com/article/10.3390/ani11102916/s1, Scenario 1—UNL Feed yard—Full trial calibration.; Scenario 2—KSU Backgrounding. Full trial calibration. Pooling.; Scenario 3—Truncated KSU trial calibration.; Scenario 4—Matched systems.; Scenario 5—Paired LWG.; Scenario 6—Coupled full system simulation.; Cow/calf operation inventory data.; System diagrams with animal flow and impacts.; Bootstrap Monte Carlo simulation statistical significance testing.; Contribution analysis.; Additional LCI datasets.
