# Supplementary material for: Analysis of Life Cycle Environmental Impacts of Using Enogen Corn in Beef Cattle Rations
Source: Animals (Basel). 2021 Oct 9;11(10):2916. doi: 10.3390/ani11102916 (PMC8532713; doi:10.3390/ani11102916)
Supplement: Supplementary file 1 [file animals-11-02916-s001.zip › animals-1403029-supplementary.pdf]

ANALYSIS OF LIFE CYCLE ENVIRONMENTAL IMPACTS OF USING ENOGEN CORN IN BEEF CATTLE RATIONS

Supplementary material

Greg Thoma, Ph.D., P.E.

Marty Matlock, Ph.D., P.E., B.C.E.E.

Martin Christy, Ph.D.

Table of Contents

Appendix A. Scenario 1 - UNL Feed yard – Full trial calibration. 5

Appendix B. Scenario 2 - KSU Backgrounding. Full trial calibration. 9

Pooling. 9

Appendix C. Scenario 3 - **Truncated KSU** trial calibration. 14

Truncation/Interpolation: 14

Appendix D. Scenario 4 – Matched systems. 19

Appendix E. Scenario 5 - Paired LWG 20

Appendix F. Scenario 6 - Coupled full system simulation 26

Feed yard: Hypothetical heavy animals 27

Appendix G. Cow/calf operation inventory data. 31

Appendix H. System diagrams with animal flow and impacts. 32

Appendix I. Bootstrap Monte Carlo simulation statistical significance testing. 42

Appendix J. Contribution analysis. 44

Appendix K. Additional LCI datasets. 55

Table of Figures

Figure C-1. Pooled, truncated cumulative weight gain for conventional treatment. 14

Figure C-2. Pooled, truncated cumulative weight gain for Enogen treatment. 14

Figure C-3. Pooled dry matter intake of backgrounders fed conventional corn 15

Figure C-4. Pooled dry matter intake of backgrounders fed Enogen Feed Corn 15

Figure H-1. Scenario 4 for retail cuts. Truncated BG; Calibrated full FY; Enogen Feed Corn. 33

Figure H-2. Scenario 4 for retail cuts. Truncated BG; Calibrated full FY; Conventional Corn 33

Figure H-3. Scenario 6 for retail cuts. Full BG; Full FY (matched LWG); Enogen Feed Corn, 34

Figure H-4. Scenario 6 for retail cuts. Full background; Full FY (matched LWG); Conventional Corn 34

Figure H-5. Scenario 5 for retail cuts. Full BG; Full FY (paired LWG); Enogen Feed Corn, Paradigm 3 35

Figure H-6. Scenario 5 for retail cuts. Full background; Full UNL (paired LWG); Conventional Corn 35

Figure H-7. Scenario 1: Calibrated UNL full trial; Enogen Feed Corn 36

Figure H-8. Scenario 1: Calibrated UNL full trial; Conventional Corn 36

Figure H-9. Scenario 2: Calibrated KSU full backgrounding trial. Enogen Feed Corn. 37

Figure H-10. Scenario 2: Calibrated KSU full backgrounding trial. Conventional Corn. 37

Figure H-11. Scenario 4: truncated BG plus calibrated FY yard. Enogen Feed Corn. 38

Figure H-12. Scenario 4: truncated BG plus calibrated FY. Conventional Corn 38

Figure H-13. Scenario 6: feed yard only; matched LWG. Enogen Feed Corn. 39

Figure H-14. Scenario 6: feed yard only; matched LWG. Conventional Corn. 39

Figure H-15. Scenario 5: background plus feed yard; paired LWG. Enogen Feed Corn. 40

Figure H-16. Scenario 5: background plus feed yard; paired LWG. Conventional Corn. 40

Figure H-17. Scenario 6: full BG plus FY; matched LWG yard. Enogen Feed Corn. 41
Figure H-18. Scenario 6: full BG plus FY; matched LWG yard. Conventional Corn. 41
Figure J-1. Scenario 1: UNL Feed Yard Calibrated Conventional Corn 46
Figure J-2. Scenario 1: UNL Feed Yard Calibrated Enogen Feed Corn 46
Figure J-3. Scenario 2: KSU Full Background Calibrated Conventional Corn 47
Figure J-4. Scenario 2: KSU Full Background Calibrated Enogen Feed Corn 47
Figure J-5. Scenario 4: Background (truncated) plus Feed Yard GtG Conventional Corn 48
Figure J-6. Scenario 4: Background (truncated) plus Feed Yard GtG Enogen Feed Corn 48
Figure J-7. Scenario 4: Cradle-to-Retail Cut; Truncated BG; Full FY Conventional Corn 49
Figure J-8. Scenario 4: Cradle-to-Retail Cut; Truncated BG; Full FY Enogen Feed Corn 49
Figure J-9. Scenario 5: Background plus Feed Yard GtG Conventional Corn - paired LWG 50
Figure J-10. Scenario 5: Background plus Feed Yard GtG Enogen Feed Corn - paired LWG 50
Figure J-11. Scenario 5: Cradle-to-Retail Cut; Conventional Corn - paired LWG 51
Figure J-12. Scenario 5: Cradle-to-Retail Cut; Enogen Feed Corn - paired LWG 51
Figure J-13. Scenario 6 Background plus Feed Yard GtG Conventional Corn - matched LWG 52
Figure J-14. Scenario 6: Background plus Feed Yard GtG Conventional Corn - matched LWG 52
Figure J-15. Scenario 6: Cradle-to-Retail Cut; Conventional Corn - matched LWG 53
Figure J-16. Scenario 6: Cradle-to-Retail Cut; Enogen Feed Corn - matched LWG 53
Figure J-17. Scenario 6: Feed Yard, Conventional Corn - matched LWG 54
Figure J-18. Scenario 6: Feed Yard, Enogen Feed Corn - matched LWG 54

Table of Tables

Table A-1. UNL Feed yard: Trial and Calibrated IFSM Data 6
Table A-2. IFSM ration characteristic input values for UNL feed trial, Scenario 1 7
Table A-3. Inventory Input to OpenLCA from UNL trial. 7
Table A-4. IFSM simulated animal performance metrics 8
Table A-5. IFSM simulated rations 8
Table B-1. KSU Backgrounding Trial and Calibrated IFSM Data 10
Table B-2. Pooled treatment data for calibration of IFSM against KSU Backgrounding trial 11
Table B-3. IFSM simulated rations for Scenario 3 12
Table B-4. IFSM simulated animal performance metrics 12
Table B-5. Inventory Input to Open LCA 13
Table C-1. Cumulative weight and dry matter intake regressed versus days on feed. 15
Table C-2. Truncation calculations-interpolation of days on feed and feed consumption. 15
Table C-3. Calibrated IFSM simulated animal performance metrics for truncated background scenario 16
Table C-4. Calibrated IFSM simulated rations for truncated background scenario 16
Table C-5. KSU Backgrounding trial and truncated / interpolated data to match UNL starting weights. 17
Table C-6. Inventory Input to Open LCA: backgrounding phase (truncated ending weights). 18
Table D-1. Environmental impacts and improvements for gate-to-gate backgrounding plus feed yard (truncated backgrounding; full feed yard). 19

Table D-2. Environmental impacts and improvements for cradle-to-harvest gate. Simulation with truncated background and matched UNL trial conditions plus generic cow calf and harvest facility models.......... 19
Table E-1. Environmental impacts and improvements for Scenario 5, full background plus full feed yard-gate-to-gate. Animals simulated for both treatments to reach the same backgrounding and finishing weights; feed yard gain from: 382 finishing at 662 kg.......... 20
Table E-2. Environmental impacts and improvements for Scenario 5: cradle-to-harvest gate. Simulation with background and modified UNL (662 kg finish weight) plus generic cow calf operation and harvest facility model.......... 21
Table E-3. KSU Backgrounding modified to produce animals at 380 kg. 22
Table E-4. UNL Feed yard trial data and calibrated IFSM information. 23
Table E-5. Inventory Input to Open LCA: backgrounding phase. 24
Table E-6. Inventory Input to Open LCA- finishing phase 25
Table F-1. Environmental impacts and improvements for Scenario 7, full background plus full feed yard-gate-to-gate. Feed yard simulated as starting with KSU end weight and maintaining the reported LWG from the Nebraska trial.......... 26
Table F-2. Environmental impacts and improvements for Scenario 5: cradle-to-harvest gate. Simulation with full background and UNL larger animals, matched feed yard LWG.......... 26
Table F-3. UNL Feed yard: Trial and Calibrated IFSM Data 28
Table F-4. Inventory Input to Open LCA for Nebraska field trial; heavy animal 29
Table F-5. IFSM simulated rations for Nebraska field trial; heavy animal 30
Table F-6. IFSM simulated animal performance metrics: Nebraska field trial, large animal.......... 30
Table G-1. Inventory Input to Open LCA: cow/calf phase. 31
Table I-1. Bootstrap Monte Carlo results. Green cells indicate p-value less than 0.01; salmon color cells indicate p-value greater than 0.01.......... 43
Table J-1. Lifecycle impact assessment results comparing CNV and EFC ration treatments across all scenarios. 44

Appendix A. Scenario 1 - UNL Feed yard – Full trial calibration.

Scenario 1 is a feed yard gate-to-gate analysis matched as closely as possible to the UNL feed yard trial results (Jolly-Breithaupt et al. 2019). This study investigates the differences in performance of finishing cattle fed rations with two different corn types, conventional field corn (CNV) and Enogen Feed corn (EFC). The observed performance results, the ration information from the study, and the IFSM calibration targets are included in the following tables. Table A-1 presents the data from the field trial in the left panel and the calibrated results from IFSM in the right panel. The IFSM information is a combination of both the input data and the calculated results. The main input data are the target starting and ending weights and the days on feed. The main output data are the final herd weight and feed consumption; the remaining parameters are calculated from these data. An important aspect of the calibration was modifying the feed ingredient characteristics and inclusion limits for individual ingredients so that the simulated ration matched as nearly as possible the reported ration from the field trial.

Modifying IFSM input files for calibration.

All calibration parameters can be found in the “Herd/Feed information” section in IFSM version 4.4 (Rotz et al. 2015). The herd size can be edited by clicking on the “Herd/feed information” button and editing the field titled “Finishing cattle” in the “Number of” section. The time spent at the feed yard and animal weight ranges can be edited by clicking on the “Management options” button within the “Herd/feed information” window and editing the “Finishing period” field and “Finish shrunk body weight goals” fields, respectively, in the “Growth period goals” section. Feed information can be edited by clicking on the “Herd/feed information” button and navigating to the “Feeding” tab. The ingredients must be selected using the drop-down menus in the “Ration constituent” section of the “Feeding” tab. The field “Relative forage to grain ratio” was set to “Low”, the “Crude protein supplement” field was set to “Urea”, the “Undegradable protein / full fat seed supplement” field was set to “Distiller’s grain”, and the “Energy supplement” field was set to “Grain”.

Once the ingredients are selected, the characteristics can be edited by clicking on the “Feed characteristics” button in the “Ration constituent” section of the “Feeding” tab. The “Feeding limit” for the distiller’s grain, urea, and corn were manually adjusted until IFSM output matched inclusion from the UNL study. The Net Energy of Maintenance (NE_m) was edited for corn silage to match inclusion from the UNL study. The identity and value of each calibration variable is included in Table A-2.

IFSM reports the mass of each purchased ingredient in metric tons. The feeding trial data was converted to cumulative metric tons consumed by multiplying the daily feed intake by the number of days on feed and then by the number of animals. This provided both individual ingredient and total mixed ration (TMR) feed consumption for direct comparison to the IFSM output. The mass of ingredients in each ration treatment is included in Table A-3 below.

The iterative calibration procedure, as explained in the LCA report, was followed until the difference between the field trial data and the IFSM outputs was less than one percent (excluding urea). Due to limited decimals on the output values, only one decimal place on the simulated urea value was available. Therefore, IFSM was calibrated to use 0.6 metric tons of urea in each ration. The observed values, IFSM simulated values, and calculated differences between these values are presented in Table A-4 and Table A-5.

Table A-1. UNL Feed yard: Trial and Calibrated IFSM Data

Observed in UNL Trial			Calibrated IFSM Input/Output		
**Animal Performance**			**Animal Performance**		
	**Conventional**	**Enogen**		**Conventional**	**Enogen**
Initial LW (kg/head)	293	294	Initial LW (kg/head)	293	294
Final LW (kg/head)	570	590	Final LW (kg/head)	571	591
Treatment size (head)	60	60	Simulated herd size (head)	60	60
Final herd LW (kg/herd)	34,200	35,400	Final herd LW (kg/herd)	34,278	35,481
Liveweight gain (kg/head)	277	296	Liveweight gain (kg/head)	278	297
Feed consumed (kg/head)	1,776	1,793	Feed consumed (kg/head)	1,767	1,790
Days on feed	166	166	Days on feed	166	166
Average daily gain (kg/day)	1.67	1.78	Average daily gain (kg/day)	1.68	1.79
G:F	0.157	0.166	G:F	0.157	0.166
**Ration composition (% DM)**			**Ration composition (% DM)**		
	**Conventional**	**Enogen**		**Conventional**	**Enogen**
Conventional Corn (CNV)	64.0	0	Conventional Corn (CNV)	64.3	0
Enogen Feed Corn (EFC)	0	64.0	Enogen Feed Corn (EFC)	0.00	64.1
Silage	15.0	15.0	Silage	15.1	15.0
DDG	15.0	15.0	DDG	15.1	15.0
Supplement*	6	6	Supplement	6	6
**Ration composition (kg/head)**			**Ration composition (kg/head)**		
	**Conventional**	**Enogen**		**Conventional**	**Enogen**
Conventional Corn	1,137	0.0	Conventional Corn	1,137	0.0
Enogen Feed Corn	0.0	1,147	Enogen Feed Corn	0.0	1,147
Silage	266.4	268.9	Silage	266.7	268.3
DDG	266.4	268.9	DDG	266.7	268.3
Supplement	106.6	107.6	Supplement	106.6	107.6

* supplement includes 0.64 t urea as reported in UNL trial

Table A-2. IFSM ration characteristic input values for UNL feed trial, Scenario 1

	Input values		
Input variable	Conventional	Enogen (EFC)	Unit
NE_m (silage)	2.77	2.36	Mcal/kg DM
Feeding limit (corn)	6.60	6.66	kg. DM/head/day
Feeding limit (urea)	0.064	0.064	kg. DM/head/day
Feeding limit (distiller's grain)	1.6	1.61	kg. DM/head/day

Table A-3. Inventory Input to OpenLCA from UNL trial.

**Inventory: Inputs to finishing**			
**Flow**	**Conventional**	**Enogen**	**Unit**
Corn grain feed	68.2	68.8	t
Diesel	318,099	318,470	MJ
DDG	16.0	16.1	t
Electricity	1.9	1.9	kWh
Silage	16.0	16.1	t
Natural gas	0.8	0.8	m^3
Vitamin premix	6.4	6.4	t
Drinking water	0.32	0.32	t
**Inventory: Outputs from finishing**			
**Flow**	**Conventional**	**Enogen**	**Unit**
Ammonia; housing	833	829	Kg
Ammonia; field emissions	61.6	61.4	Kg
Ammonia; manure	271	265	Kg
Dinitrogen monoxide; housing	42.3	41.6	Kg
Dinitrogen monoxide; animals	4.8	4.8	Kg
Dinitrogen monoxide; manure	13.6	13.5	Kg
Dinitrogen monoxide; indirect	15.8	15.7	Kg
Hydrogen sulfide; housing	6.7	6.6	Kg
Methane, non-fossil; manure storage	587	581	kg
Methane, non-fossil; field emissions	3.1	3.1	kg
Methane, non-fossil; animal	962	994	kg
Methane, non-fossil; housing manure	39.3	39.7	kg
Nitrogen; leaching	92.0	91.0	kg
Nitrogen; runoff	9.0	9.0	kg
NMVOC*; housing	5.3	5.3	kg
NMVOC*; field and grazing	1.7	1.7	kg
Product			
**Flow**	**Conventional**	**Enogen**	**Unit**
Finished animal	34,278	35,481	kg (LW)
Finished animal	16,698	17,841	kg (LWG)

*Non-methane volatile organic compounds

Table A-4. IFSM simulated animal performance metrics

**Conventional Corn (CNV)**			
Ingredient	*Study values (metric Tons)*	*IFSM values (metric Tons)*	*IFSM relative error*
Final herd liveweight (kg)	34,200	34,278	0.23%
Final animal liveweight (kg)	570	571	0.18%
Total feed consumed by the herd (kg)	106,572	106,020	-0.52%
Gain to feed ratio (kg gain / kg feed intake)	0.157	0.157	0.0%
**Enogen Feed Corn (EFC)**			
Ingredient	*Study values (metric Tons)*	*IFSM values (metric Tons)*	*IFSM relative error*
Final herd liveweight (kg)	35,400	35,481	0.23%
Final animal liveweight (kg)	590	591	0.17%
Total feed consumed by the herd (kg)	107,568	107,400	-0.16%
Gain to feed ratio (kg gain / kg feed intake)	0.166	0.166	0.0%

Table A-5. IFSM simulated rations

**Conventional Corn (CNV)**			
Ingredient	Study values (metric Tons)	IFSM values (metric Tons)	IFSM relative error
Corn (CNV)	68.2	68.2	-0.01%
Corn silage	15.99	16	0.09%
Distiller's grain	15.99	16	0.09%
Urea	0.64	0.6	6.1%
**Enogen Feed Corn (EFC)**			
Ingredient	Study values (metric Tons)	IFSM values (metric Tons)	IFSM relative error
Corn (EFC)	68.8	68.8	-0.06%
Corn silage	16.1	16.1	-0.22%
Distiller's grain	16.1	16.1	-0.22%
Urea	0.645	0.6	7%

Appendix B. Scenario 2 - KSU Backgrounding. Full trial calibration.

Scenario 2 is a gate-to-gate simulation of the backgrounding operation matched as closely as possible to the KSU backgrounding trial (Johnson et al. 2018). Table B-1 presents the trial data and calibrated IFSM simulation information. As in the other sections, the left panel shows the data from the field trial and the right panel shows the calibrated information used as inputs to the IFSM and the simulated outputs as described above. Table B-2 presents the pooled information used for calibration as described below. Summary calibration information as quantity consumed animal performance with the percent difference from the field trial data is given in Table B-4 and Table B-3. Table B-5 presents the input and emission inventory data for OpenLCA.

Pooling.

Within each corn type treatment (CNV or EFC), two corn processing treatments were evaluated in the field trial. These included dry-rolled corn (DRC) and whole corn (WC). For this evaluation, we pooled the two processing treatments. Specifically, the weighted average of cumulative body weight, dry matter intake, and gain to feed ratio (Table B-2) was calculated for the two processing types within each corn type treatment. The purpose of pooling the treatment data was to focus the study on effect of corn type and to ignore the effect of corn processing technology on animal performance.

The pooling process is described by the following equations, let $x_{t,p}$ represent a measurement (weight gain, dry matter intake, or gain to feed ratio) for ration treatment with corn type t (CNV or EFC) and processing type p (DRC or WC), $n_{t,p}$ represent the number of animals in the treatment with corn type t and processing type p, and $x_{AVG,t}$ represent the weighted average of the two processing types within a corn type t.

Conventional: $x_{AVG,CNV} = \frac{n_{CNV,DRC} * x_{CNV,DRC} + n_{CNV,WC} * x_{CNV,WC}}{n_{CNV,DRC} + n_{CNV,WC}}$

Enogen: $x_{AVG,EFC} = \frac{n_{EFC,DRC} * x_{EFC,DRC} + n_{EFC,WC} * x_{EFC,WC}}{n_{EFC,DRC} + n_{EFC,WC}}$

This calculation was completed for each entry of each observation-that is at each time data were reported. The tables below include the data used for this calculation in addition to the result.

Table B-1. KSU Backgrounding Trial and Calibrated IFSM Data

KSU background trial, observed data			Calibrated IFSM input/output		
**Animal Performance**			**Animal Performance**		
	**Conventional**	**Enogen**		**Conventional**	**Enogen**
Initial LW (kg/head)	244.5	244.5	Initial LW (kg/head)	244	244
Final LW (kg/head)	382	386	Final LW (kg/head)	382	385
Treatment size (head)	190	189	Simulated herd size (head)	190	189
Final herd LW (kg/herd)	72,675	72,954	Final herd LW (kg/herd)	72,586	72,772
Liveweight gain (kg/head)	138.0	141.5	Liveweight gain (kg/head)	138.0	141.0
Feed consumed (kg/head)	870.4	843.6	Feed consumed (kg/head)	870.7	843.7
Days on feed	91	91	Days on feed	91	91
Average daily gain (kg/day)	1.52	1.55	Average daily gain (kg/day)	1.52	1.55
G:F	0.159	0.168	G:F	0.159	0.167
**Ration composition (% DM)**			**Ration composition (% DM)**		
	**Conventional**	**Enogen**		**Conventional**	**Enogen**
Field Corn (CNV)	28.6	0.0	Field Corn (CNV)	28.6	0.0
Enogen Feed Corn (EFC)	0.0	28.6	Enogen Feed Corn (EFC)	0.0	28.6
Hay	35.0	35.0	Hay	35.0	35.0
DDG	30.0	30.0	DDG	30.0	30.0
Supplement	6.4	6.4	Supplement	6.4	6.4
**Ration composition (kg/head)**			**Ration composition (kg/head)**		
	**Conventional**	**Enogen**		**Conventional**	**Enogen**
Field Corn	248.7	0.0	Field Corn	248.9	0.0
Enogen Feed Corn	0.0	241.0	Enogen Feed Corn	0.0	241.3
Hay	304.6	295.3	Hay	304.7	295.2
DDG	261.1	253.1	DDG	261.1	252.9
Supplement	56.0	54.2	Supplement	55.8	54.2

Table B-2. Pooled treatment data for calibration of IFSM against KSU Backgrounding trial

				Days on feed						
				0	7	14	35	63	77	91
**Conventional**	*Feed Intake*	*Dry rolled corn*	*kg feed/day*	-	6.62	7.71	8.58	9.08	9.13	9.44
		Whole Corn	*kg feed/day*	-	6.57	7.61	8.54	9.2	9.34	9.69
		Pooled CNV	*kg feed/day*	-	6.6	7.61	8.56	9.12	9.23	9.55
	Gain:Feed	*Dry rolled corn*	*kg gain/kg feed*	-	0.302	0.307	0.21	0.175	0.165	0.164
		Whole Corn	*kg gain/kg feed*	-	0.304	0.279	0.201	0.167	0.16	0.154
		Pooled CNV	*kg gain/kg feed*	-	0.303	0.291	0.205	0.171	0.162	0.159
	Live Weight	*Dry rolled corn*	*kg liveweight*	244	258	277	307	344	360	385
		Whole Corn	*kg liveweight*	245	259	274	305	341	360	380
		Pooled CNV	*kg liveweight*	244.5	258.5	275.5	306	342.5	360	382.5
**Enogen**	*Feed Intake*	*Dry rolled corn*	*kg feed/day*	-	6.45	7.47	8.56	9.02	9.03	9.3
		Whole Corn	*kg feed/day*	-	6.24	7.3	8.12	8.78	8.9	9.24
		Pooled EFC	*kg feed/day*	-	6.33	7.369	8.305	8.902	8.973	9.263
	Gain:Feed	*Dry rolled corn*	*kg gain/kg feed*	-	0.325	0.294	0.211	0.182	0.176	0.168
		Whole Corn	*kg gain/kg feed*	-	0.332	0.299	0.218	0.18	0.174	0.168
		Pooled EFC	*kg gain/kg feed*	-	0.327	0.296	0.213	0.181	0.175	0.168
	Live Weight	*Dry rolled corn*	*kg liveweight*	244	259	275	307	348	367	386
		Whole Corn	*kg liveweight*	245	259	275	306	344	364	386
		Pooled EFC	*kg liveweight*	244.5	259	275	306.5	346	365.5	386

Table B-3. IFSM simulated rations for Scenario 3

**Conventional Corn (CNV)**			
Ingredient	*Study values (metric Tons)*	*IFSM values (metric Tons)*	*IFSM relative error*
Corn (CNV)	47.25	47.3	0.11%
Hay mix	57.88	57.9	0.03%
Distiller's grain	49.61	49.6	-0.03%
**Enogen Feed Corn (EFC)**			
Ingredient	*Study values (metric Tons)*	*IFSM values (metric Tons)*	*IFSM relative error*
Corn (EFC)	45.55	45.6	0.10%
Hay mix	55.81	55.8	-0.01%
Distiller's grain	47.83	47.8	-0.07%

Table B-4. IFSM simulated animal performance metrics

**Conventional Corn (CNV)**			
Ingredient	*Study values (metric Tons)*	*IFSM values (metric Tons)*	*IFSM relative error*
Final herd liveweight (kg)	72,675	72,586	-0.12%
Final animal liveweight (kg)	382.5	3823	-0.12%
Total feed consumed by the herd (kg)	165,376	165,433	0.03%
Gain to feed ratio (kg gain / kg feed intake)	0.159	0.159	0.0%
**Enogen Feed Corn (EFC)**			
Ingredient	*Study values (metric Tons)*	*IFSM values (metric Tons)*	*IFSM relative error*
Final herd liveweight (kg)	72,954	72,772	-0.25%
Final animal liveweight (kg)	386	385	-0.25%
Total feed consumed by the herd (kg)	159,440	159,459	-0.01%
Gain to feed ratio (kg gain / kg feed intake)	0.168	0.167	-0.6%

Table B-5. Inventory Input to Open LCA

Inventory: Inputs to full backgrounding simulation			
**Flow**	**Conventional**	**Enogen**	**Unit**
Corn grain feed	47.3	45.6	t
Diesel	220,213	219,743	MJ
DDG	49.6	47.8	t
Electricity	3,225	3,208	kWh
Hay mix	57.9	55.8	t
Natural gas	550	531	m3
Vitamin premix	10.6	10.3	t
Drinking water	413	413	t
**Inventory: Outputs from full backgrounding simulation**			
**Flow**	**Conventional**	**Enogen**	**Unit**
Ammonia; field emissions	51	50	kg
Ammonia; housing	1,553	1,471	
Dinitrogen monoxide; animals	9	9	kg
Dinitrogen monoxide; indirect	24	22	kg
Dinitrogen monoxide; housing	98	97	kg
Hydrogen sulfide; housing	12	11	kg
NMVOC*; field and grazing	4	4	kg
NMVOC*; housing	9	8	
Methane, non-fossil; animal	2,558	2,525	
Methane, non-fossil; housing manure	70	67	kg
Methane, non-fossil; filed emission	3	3	kg
Nitrogen; leaching	240	229	kg
**Product**			
**Flow**	**Conventional**	**Enogen**	**Unit**
Backgrounded cattle	72,586	72,772	kg (LW)
Backgrounded cattle	26,226	26,656	kg (LWG)

*Non-methane volatile organic compounds

Appendix C. Scenario 3 - Truncated KSU trial calibration.

Scenario 3 describes the supporting calculations for the base case cradle-to-harvest gate analysis of beef cattle. This scenario describes the calculations necessary to perform the link of the simulated KSU backgrounding study with the simulated UNL feed yard study. The purpose of this analysis is to evaluate the environmental effects of Enogen Feed Corn fed in rations in a full cattle production system. Time series data in the backgrounding trial allows interpolation of the animal performance to closely match the reported starting weights from the UNL feed yard trial.

Truncation/Interpolation:

This analysis uses the pooled data introduced in Scenario 2 (Table B-2). However, in this analysis the full KSU backgrounding data set is truncated at the starting weights of animals in the UNL feed yard study, which are 293 kg and 294 kg, for the CNV and EFC treatments, respectively. This means the mass of feed required to raise cattle to 293 and 294 kg in the KSU feeding trial must be determined. First, the cumulative weight gain from day 0 to day 35 is plotted versus the number of days on feed (DoF) when the trial weights exceed the UNL starting weights. The truncated pooled data set is tabulated and plotted (Figure C-1 and Figure C-2) as cumulative weight gain versus DoF for both CNV and EFC treatments.

The truncated pooled data set is tabulated and plotted versus the number of DoF for both CNV and EFC treatments (Figure C-3 and Figure C-4). The regression curves for cumulative weight versus DoF and average DMI versus DoF were calculated and are included in Table C-1, where the independent variable x is the number of DoF, and y is either cumulative weight or dry matter intake.

The next step used the Goal Seek plugin from Excel to determine the number of days on feed required, for both CNV- and EFC-fed cattle, to reach the respective feed yard starting weights. The regression equation was inverted to determine the number of DoF to reach the target feed yard starting weight. The calculated DoF were used as inputs to the regressions for dry matter intake versus days on feed to calculate the average dry matter intake of finished backgrounders. The cumulative feed intake was calculated by multiplying the days on feed times the respective dry matter intake. A summary of the calculated results is presented in Table C-2.

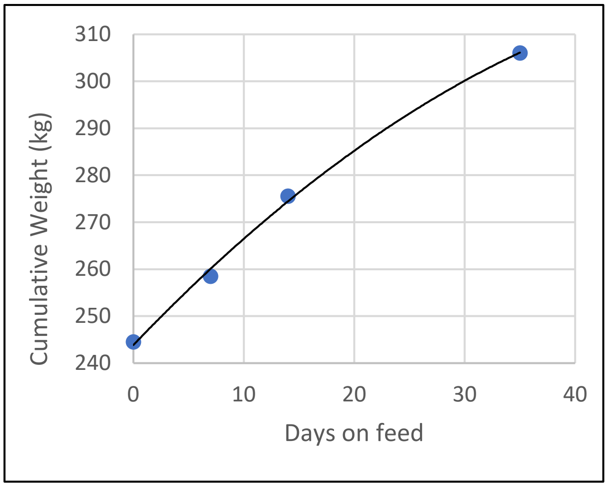

Figure C-1. Pooled, truncated cumulative weight gain for conventional treatment.

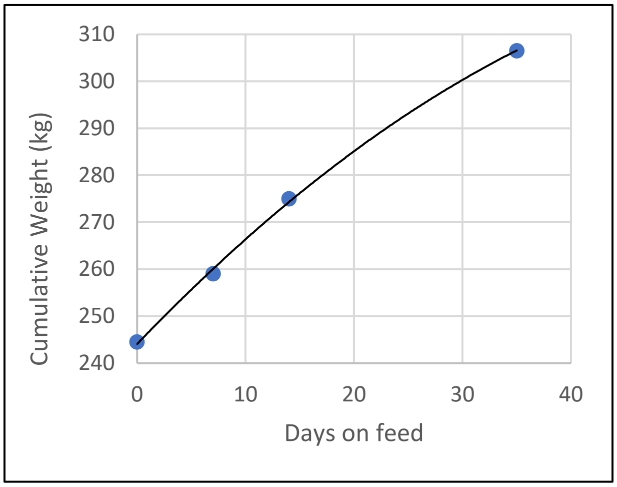

Figure C-2. Pooled, truncated cumulative weight gain for Enogen treatment.

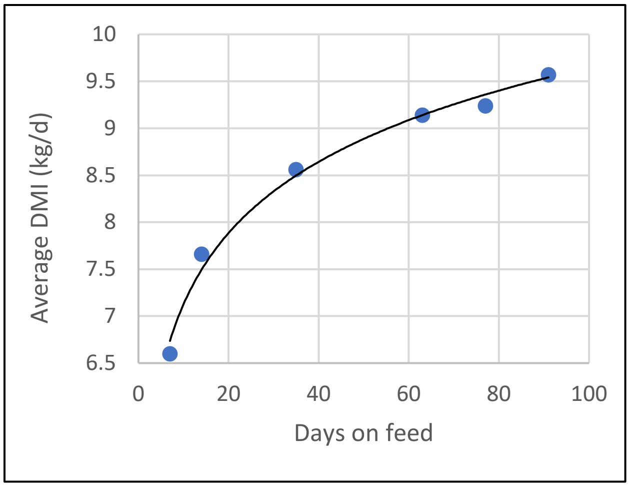

Figure C-3. Pooled dry matter intake of backgrounders fed conventional corn

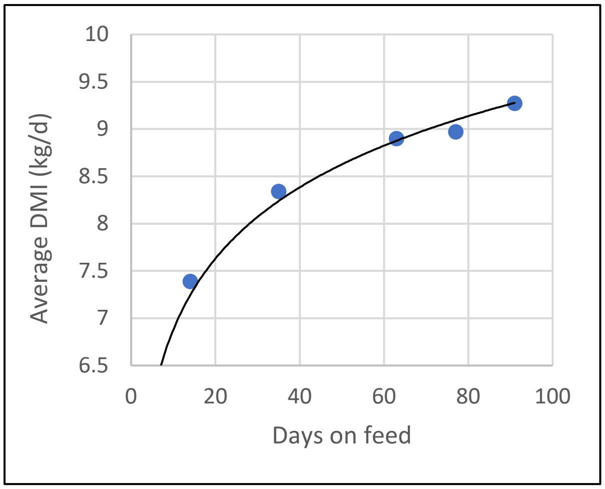

Figure C-4. Pooled dry matter intake of backgrounders fed Enogen Feed Corn

Table C-1. Cumulative weight and dry matter intake regressed versus days on feed.

**Cumulative weight versus days on feed**		
Treatment	*Regression equation*	R^2
Conventional	Eq. (1) $y = -0.0193 * x^2 + 2.45 * x + 243.9$	0.998
Enogen	Eq. (2) $y = -0.0178 * x^2 + 2.41 * x + 244.1$	0.999
**Dry matter intake versus days on feed**		
Treatment	*Regression equation*	R^2
Conventional	Eq. (3) $y = 1.094 * \ln(x) + 4.608$	0.990
Enogen	Eq. (4) $y = 1.086 * \ln(x) + 4.376$	0.989

Table C-2. Truncation calculations-interpolation of days on feed and feed consumption.

Parameter	*Conventional*	*Enogen*	*Unit*	*Note*
Liveweight	293	294	kg. liveweight	Target ending weights
Days on feed	24.9	25. 6	Days	Inversion solution for DoF
Dry matter intake	8.13	7.9	kg. feed/day	Calculated: Input DoF as the variable x in the dry matter intake regressions in Table C-1.
Cumulative feed consumed	202.3	201.9	kg. feed	Calculated: DoF * DMI

Table C-3 and Table C-4 present the calibration information and statistics for the truncated backgrounding simulation. The calibration tolerances are slightly looser than for other situations; however, because the animals are simulated with approximately 25 days on feed, the calibration quality will not have a significant effect on the calculated results.

Table C-3. Calibrated IFSM simulated animal performance metrics for truncated background scenario

**Conventional Corn (CNV)**			
Ingredient	*Study values (metric Tons)*	*IFSM values (metric Tons)*	*IFSM relative error*
Final herd liveweight (kg)	55,670	55,552	-0.21%
Final animal liveweight (kg)	293	292.4	-0.20%
Total feed consumed by the herd (kg)	38,440	38,470	0.08%
Gain to feed ratio (kg gain / kg feed intake)	0.240	0.236	-1.37%
**Enogen Feed Corn (EFC)**			
Ingredient	*Study values (metric Tons)*	*IFSM values (metric Tons)*	*IFSM relative error*
Final herd liveweight (kg)	55,566	55,445	-0.22%
Final animal liveweight (kg)	294	293.36	-0.22%
Total feed consumed by the herd (kg)	38,150	38,250	0.26%
Gain to feed ratio (kg gain / kg feed intake)	0.245	0.241	-1.56%

Table C-4. Calibrated IFSM simulated rations for truncated background scenario

**Conventional Corn (CNV)**			
Ingredient	*Study values*	*IFSM values*	*IFSM relative error*
Corn (CNV) *(metric Tons)*	10.98	11.00	0.17%
Hay mix *(metric Tons)*	13.45	13.5	0.35%
Distiller's grain *(metric Tons)*	11.53	11.5	-0.27%
**Enogen Feed Corn (EFC)**			
Ingredient	*Study values*	*IFSM values*	*IFSM relative error*
Corn (EFC) *(metric Tons)*	10.9	11.00	0.92%
Hay mix *(metric Tons)*	13.35	13.4	0.36%
Distiller's grain *(metric Tons)*	11.44	11.4	-0.39%

Table C-5 presents the summary data of the calibration target and IFSM inputs and outputs on the left and right panels, respectively. As with previous tables, the output data are row and computed from I FSM inputs and outputs.

Table C-5. KSU Backgrounding trial and truncated / interpolated data to match UNL starting weights.

**Truncated, pooled KSU trial (match UNL starting weights)**			**Calibrated, truncated IFSM Input/Output**		
**Animal Performance**			**Animal Performance**		
	**Conventional**	**Enogen**		**Conventional**	**Enogen**
Initial LW (kg/head)	244.5	244.5	Initial LW (kg/head)	244.4	244.4
Final LW (kg/head)	293	294	Final LW (kg/head)	292.4	293.4
Treatment size (head)	190	189	Simulated herd size (head)	190	189
Final herd LW (kg/herd)	55,670	55,566	Final herd LW (kg/herd)	55,552	55,445
Liveweight gain (kg/head)	48.5	49.5	Liveweight gain (kg/head)	47.9	48.9
Feed consumed (kg/head)	202.3	201.9	Feed consumed (kg/head)	202.5	202.4
Days on feed	24.9	25.6	Days on feed	24.9	25.6
Average daily gain (kg/day)	1.95	1.94	Average daily gain (kg/day)	1.93	1.91
G:F	0.240	0.245	G:F	0.237	0.242
**Ration composition (% DM)**			**Ration composition (% DM)**		
	**Conventional**	**Enogen**		**Conventional**	**Enogen**
Field Corn (CNV)	28.57	0	Field Corn (CNV)	28.6	0.0
Enogen Feed Corn (EFC)	0	28.6	Enogen Feed Corn (EFC)	0.0	28.8
Hay	35	35	Hay	35.1	35.0
DDG	30	30	DDG	29.9	29.8
Supplement	6.43	6.43	Supplement	6.4	6.4
**Ration composition (kg/head)**			**Ration composition (kg/head)**		
	**Conventional**	**Enogen**		**Conventional**	**Enogen**
Field Corn	57.8	0.0	Field Corn	57.9	0.0
Enogen Feed Corn	0.0	57.7	Enogen Feed Corn	0.0	58.2
Hay	70.8	70.6	Hay	71.1	70.9
DDG	60.7	60.6	DDG	60.5	60.3
Supplement	13.0	13.0	Supplement	13.0	13.0

Table C-6 presents the input and emission inventory used for the foreground processes in the open LCA platform. The inventory for the feed yard process is presented in Appendix A, Scenario 1.

Table C-6. Inventory Input to Open LCA: backgrounding phase (truncated ending weights).

**Inventory: Inputs to backgrounding (truncated)**			
**Flow**	**Conventional**	**Enogen**	**Unit**
Corn grain feed	11.0	11.0	t
Diesel	209,828	209,786	MJ
DDG	11.5	11.4	t
Electricity	879	897	kWh
Hay	13.5	13.4	t
Natural gas	128	128	m3
Vitamin premix	2.5	2.5	t
Drinking water	99.9	102.1	t
**Inventory: Outputs from backgrounding (truncated)**			
**Flow**	**Conventional**	**Enogen**	**Unit**
Ammonia; housing	333	326	kg
Ammonia; field emissions	12.0	12.0	kg
Dinitrogen monoxide; housing	25.0	25.0	kg
Dinitrogen monoxide; animals	2.0	2.0	kg
Dinitrogen monoxide; indirect	5.0	5.0	kg
Hydrogen sulfide; housing	3.0	3.0	kg
Methane, non-fossil; animal	656	658	kg
Methane, non-fossil; housing manure	16.0	16.0	kg
Nitrogen; leaching	52.0	52.0	kg
NMVOC*; housing	2.0	2.0	kg
NMVOC*; field and grazing	1.0	1.0	kg
**Product**			
**Flow**	**Conventional**	**Enogen**	**Unit**
Backgrounded cattle	55,552	55,445	kg (LW)
Backgrounded cattle	9,108	9,246	kg (LWG)

*Non-methane volatile organic compounds

Appendix D. Scenario 4 – Matched systems.

The lifecycle inventory data for the results presented below are taken from Scenario 3 for the backgrounding phase and from Scenario 1 for the feed yard phase. The comparative result for the combined feeding phases in terms of the impact per 1000 kg LW G are presented in Table D-1. For the cradle-to-harvest gate results presented in Table D-2, the feeding stages were coupled with a generic cow calf and generic harvesting operation.

Table D-1. Environmental impacts and improvements for gate-to-gate backgrounding plus feed yard (truncated backgrounding; full feed yard).

Impact category	units	Conventional	Enogen	Enogen percent decrease in impact
Climate change	(kg CO_2eq/ 1,000 kg LWG)	8,546 [a]	8,076 [b]	-5.49%
Land use	(m^2a/ 1,000 kg LWG)	13,414 [a]	12,638 [b]	-5.78%
Water use	(m^3/ 1,000 kg LWG)	1,284 [a]	1,218 [b*]	-5.10%
Fossil Energy	(kg oil eq/ 1,000 kg LWG)	1,184 [a]	1,117 [b]	-5.72%

Values with different letters within a category are significantly different ($p<0.01$).

*$p<0.03$ for water use, thus there is 97% rather than 99+% confidence that the treatments are different.

Table D-2. Environmental impacts and improvements for cradle-to-harvest gate. Simulation with truncated background and matched UNL trial conditions plus generic cow calf and harvest facility models.

Impact category	units	Conventional	Enogen	Enogen percent decrease in impact
Climate change	(kg CO_2eq/ 1,000 kg retail cut)	30,071 [a]	29,233 [b]	-2.79%
Land use	(m^2a/ 1,000 kg retail cut)	132,641 [a]	128,636 [b]	-3.02%
Water use	(m^3/ 1,000 kg retail cut)	1,712 [a]	1,674 [b]	-2.21%
Fossil Energy	(kg oil eq/ 1,000 kg retail cut)	1,973 [a]	1,925 [b]	-2.48%

Values with different letters within a category (row) are significantly different ($p<0.01$).

Appendix E. Scenario 5 - Paired LWG

The information presented in this appendix refers to the "Paired LWG" scenario. For this test, we set the final backgrounding weight for both treatments, conventional and Enogen to 380 kg, and subsequently coupled these simulated performance data to a feed yard simulation in which animals from each treatment started at 380 kg and finished ag 662 kg. Table E-1 presents the comparison of impacts for this scenario from the backgrounding-to-feed yard gate perspective, and Table E-2 resents the same comparison for the cradle-to-harvest gate system boundary. The uncertainty analysis shows that land and water use are significantly different for the scenarios, but that climate change and fossil energy are not statistically different.

Table E-3 and Table E-4 present the data used for calibrating IFSM for the modified backgrounding (modified finishing weight) and for the modified feed yard trial (modified starting and finishing weights-held constant for both feed treatments), respectively. The backgrounding trial simulation files were modified from the calibrated full trial simulation files (Appendix B) by adjusting the ending weight to 380 kg. The feed yard trial input data was modified to start animals at 380 kg and finished them at 662 kg which is approximately the average live weight gain reported for the two treatments. This scenario is included as a robustness test and eliminates the observed interaction in the field trial between feed conversion and cumulative weight gain. The IFSM simulation input files were not modified beyond the change in animal weights.

Table E-5 and Table E-6 present the input and emissions data used in open LCA for full system simulation.

Table E-1. Environmental impacts and improvements for Scenario 5, full background plus full feed yard-gate-to-gate. Animals simulated for both treatments to reach the same backgrounding and finishing weights; feed yard gain from: 382 finishing at 662 kg.

Impact category	units	Conventional	Enogen	Enogen percent decrease in impact
Climate change	(kg CO_2eq/ 1,000 kg retail cut)	8,286 [a]	8,131 [a]	-1.87%
Land use	(m^2*a/ 1,000 kg retail cut)	11,442 [a]	11,269 [b]	-1.51%
Water use	(m^3/ 1,000 kg retail cut)	1,310 [a]	1,264 [b]	-3.52%
Fossil Energy	(kg oil eq/ 1,000 kg retail cut)	1,021 [a]	1,010 [a]	-1.13%

Values with different letters within a category (row) are significantly different ($p<0.01$).

Table E-2. Environmental impacts and improvements for Scenario 5: cradle-to-harvest gate. Simulation with background and modified UNL (662 kg finish weight) plus generic cow calf operation and harvest facility model.

Impact category	units	Conventional	Enogen	Enogen percent decrease in impact
Climate change	(kg CO_2eq/ 1,000 kg LWG)	28,387 [a]	28,337 [a]	-0.177%
Land use	(m^2*a/ 1,000 kg LWG)	117,308 [a]	117,261 [b]	-0.040%
Water use	(m^3/ 1,000 kg LWG)	1,875 [a]	1,859 [b]	-0.864%
Fossil Energy	(kg oil eq/ 1,000 kg LWG)	1,869 [a]	1,865 [a]	-0.195%

Values with different letters within a category (row) are significantly different ($p<0.01$).

Table E-3. KSU Backgrounding modified to produce animals at 380 kg.

KSU background trial, observed data			Modified IFSM Input/Output backgrounders finished at 380 kg.		
**Animal Performance**			**Animal Performance**		
	**Conventional**	**Enogen**		**Conventional**	**Enogen**
Initial LW (kg/head)	244.5	244.5	Initial LW (kg/head)	244.4	244.4
Final LW (kg/head)	382	386	Final LW (kg/head)	380	380
Treatment size (head)	190	189	Simulated herd size (head)	190	189
Final herd LW (kg/herd)	72,675	72,954	Final herd LW (kg/herd)	72,200	71,820
Liveweight gain (kg/head)	138.0	141.5	Liveweight gain (kg/head)	135.6	135.6
Feed consumed (kg/head)	870	844	Feed consumed (kg/head)	860.2	831.0
Days on feed	91	91	Days on feed	91	91
Average daily gain (kg/day)	1.52	1.55	Average daily gain (kg/day)	1.49	1.49
G:F	0.159	0.168	G:F	0.158	0.163
**Ration composition (% DM)**			**Ration composition (% DM)**		
	**Conventional**	**Enogen**		**Conventional**	**Enogen**
Field Corn (CNV)	28.6	0	Field Corn (CNV)	28.9	0.0
Enogen Feed Corn (EFC)	0	28.6	Enogen Feed Corn (EFC)	0.0	29.0
Hay	35	35	Hay	34.2	34.0
DDG	30	30	DDG	30.3	30.4
Supplement	6.43	6.43	Supplement	6.50	6.53
**Ration composition (kg/head)**			**Ration composition (kg/head)**		
	**Conventional**	**Enogen**		**Conventional**	**Enogen**
Field Corn	248.7	0.0	Field Corn	248.9	0.0
Enogen Feed Corn	0.0	241.0	Enogen Feed Corn	0.0	241.3
Hay	304.6	295.3	Hay	294.2	282.5
DDG	261.1	253.1	DDG	261.1	252.9
Supplement	55.97	54.24	Supplement	55.9	54.2

Table E-4. UNL Feed yard trial data and calibrated IFSM information.

	Observed in UNL Trial		Modified starting weight scenario: IFSM Input/Output		
**Animal Performance**			**Animal Performance**		
	**Conventional**	**Enogen**		**Conventional**	**Enogen**
Initial LW (kg/head)	293	294	Initial LW (kg/head)	380	380
Final LW (kg/head)	570	590	Final LW (kg/head)	662	662
Treatment size (head)	60	60	Treatment size (head)	60	60
Final herd LW (kg/herd)	34,200	35,400	Final herd LW (kg/herd)	39,720	39,720
Liveweight gain (kg/head)	277	296	Liveweight gain (kg/head)	282	282
Feed consumed (kg/head)	1,776	1,793	Feed consumed (kg/head)	1,794	1,745
Days on feed	166	166	Days on feed	166	166
Average daily gain (kg/day)	1.67	1.78	Average daily gain (kg/day)	1.70	1.70
G:F	0.157	0.166	G:F	0.157	0.162
**Ration composition (% DM)**			**Ration composition (% DM)**		
	**Conventional**	**Enogen**		**Conventional**	**Enogen**
Conventional Corn (CNV)	64.0	0.0	Conventional Corn (CNV)	63.3	0.0
Enogen Feed Corn (EFC)	0.0	64.0	Enogen Feed Corn (EFC)	0.0	65.7
Hay	15.0	15.0	Hay	15.9	12.8
DDG	15.0	15.0	DDG	14.9	15.4
Supplement	6.0	6.0	Supplement	5.4	5.5
**Ration composition (kg/head)**			**Ration composition (kg/head)**		
	**Conventional**	**Enogen**		**Conventional**	**Enogen**
Conventional Corn (CNV)	1,137	0.0	Conventional Corn (CNV)	1,137	0.0
Enogen Feed Corn (EFC)	0.0	1,147	Enogen Feed Corn (EFC)	0.0	1,148
Silage	266.4	269	Silage	285	223
DDG	266.4	269	DDG	267	268
Supplement	106.6	107.6	Supplement	107	108

Table E-5. Inventory Input to Open LCA: backgrounding phase.

Inventory: Inputs to backgrounding			
**Flow**	**Conventional**	**Enogen**	**Unit**
Corn grain feed	47.3	45.6	t
Diesel	220,043	219,530	MJ
DDG	49.6	47.8	t
Electricity	3,225	3,208	kWh
Hay	55.9	53.4	t
Natural gas	550	531	m3
Vitamin premix	10.6	10.3	t
Drinking water	411	410	t
**Inventory: Outputs from backgrounding**			
**Flow**	**Conventional**	**Enogen**	**Unit**
Ammonia; housing	1,530	1,455	kg
Ammonia; field emissions	51	49	kg
Dinitrogen monoxide; animals	9	8	kg
Dinitrogen monoxide; indirect	23	22	kg
Dinitrogen monoxide; housing	99	97	kg
Hydrogen sulfide; housing	12	11	kg
NMVOC*; housing	9	8	kg
NMVOC*; field and grazing	4	4	kg
Methane, non-fossil; housing manure	68	66	kg
Methane, non-fossil; animal	2,480	2,431	Kg
Methane, non-fossil; filed emission	3	3	kg
Nitrogen; leaching	237	225	kg
**Product**			
**Flow**	**Conventional**	**Enogen**	**Unit**
Backgrounded cattle	72,200	71,820	kg (LW)
Backgrounded cattle	25,756	25,621	kg (LWG)

*Non-methane volatile organic compounds

Table E-6. Inventory Input to Open LCA- finishing phase

Inventory: Inputs to finishing			
**Flow**	**Conventional**	**Enogen**	**Unit**
Corn grain feed	68.2	68.8	t
Diesel	369,786	369,786	MJ
DDG	16.0	16.1	t
Electricity	1,880	1,880	kWh
Silage	17.1	13.4	t
Natural gas	794	801	m3
Vitamin premix	5.8	5.8	t
Drinking water	322	323	t
**Inventory: Outputs from finishing**			
**Flow**	**Conventional**	**Enogen**	**Unit**
Ammonia; housing	846	806	kg
Ammonia; field emissions	63	59	kg
Ammonia; manure	270	263	kg
Dinitrogen monoxide; housing	42	42	kg
Dinitrogen monoxide; animals	4.8	4.7	kg
Dinitrogen monoxide; manure	14	13	kg
Dinitrogen monoxide; indirect	16	15	kg
Hydrogen sulfide; housing	6.6	6.8	kg
Methane, non-fossil; manure storage	592	567	kg
Methane, non-fossil; field emissions	3.1	2.9	kg
Methane, non-fossil; animal	974	967	kg
Methane, non-fossil; housing manure	40	37	kg
Nitrogen; leaching	93	89	kg
Nitrogen; runoff	10	9.0	kg
NMVOC*; housing	5.4	5.1	kg
NMVOC*; field and grazing	1.8	1.6	kg
**Product**			
**Flow**	**Conventional**	**Enogen**	**Unit**
Finished cattle	39,720	39,720	kg (LW)
Finished cattle	16,920	16,920	kg (LWG)

*Non-methane volatile organic compounds

Appendix F. Scenario 6 - Coupled full system simulation

This scenario is part of the robustness of the evaluation associated with the mismatched background ending and feed yard starting weights. The KSU backgrounding simulation was based on the pooled data from rolled and whole corn treatments. Unmodified full backgrounding trial IFSM input simulation files were linked with a modified feed yard simulation. For the feed yard, the same LWG from the original calibrated UNL data set was maintained. The originally calibrated IFSM input farm file was only edited to reflect heavier feeders entering the feed yard while maintaining the UNL LWGs, hence resulting in larger animals sent to harvest.

Table F-1 presents the overall impact category comparison for the conventional versus Enogen Feed corn treatments for this scenario for the gate-to-gate of backgrounding plus feed yard. Table F-2 presents the impact comparison for the cradle-to-harvest gate supply chain.

Table F-1. Environmental impacts and improvements for Scenario 7, full background plus full feed yard-gate-to-gate. Feed yard simulated as starting with KSU end weight and maintaining the reported LWG from the Nebraska trial.

Impact category	units	Conventional	Enogen	Enogen percent decrease in impact
Climate change	(kg CO_2eq/ 1,000 kg LWG)	8,559 [a]	8,172 [b]	-4.52%
Land use	(m^2*a/ 1,000 kg LWG)	11,733 [a]	11,160 [b]	-4.88%
Water use	(m^3/ 1,000 kg LWG)	1,381 [a]	1,323 [b]	-4.22%
Fossil Energy	(kg oil eq/ 1,000 kg LWG)	1,043 [a]	991 [b]	-4.96%

Values with different letters within a category (row) are significantly different ($p<0.01$).

Table F-2. Environmental impacts and improvements for Scenario 5: cradle-to-harvest gate. Simulation with full background and UNL larger animals, matched feed yard LWG

Impact category	units	Conventional	Enogen	Enogen percent decrease in impact
Climate change	(kg CO_2eq/ 1,000 kg retail cut)	28,718 [a]	27,943 [b]	-2.70%
Land use	(m^2*a/ 1,000 kg retail cut)	117,730 [a]	114,224 [b]	-2.98%
Water use	(m^3/ 1,000 kg retail cut)	1,957 [a]	1,915 [b]	-2.18%
Fossil Energy	(kg oil eq/ 1,000 kg retail cut)	1,891[a]	1,843 [b]	-2.54%

Values with different letters within a category (row) are significantly different ($p<0.01$).

Feed yard: Hypothetical heavy animals

This section describes a feed yard gate-to-gate analysis based on a modification of Scenario 1. This assessment was conducted as part of the robustness evaluation of the overall conclusions, as discussed in the main report. For this case, the calibrated input farm file developed for Scenario 1 was modified so that the animal starting and finishing weights were changed to simulate CNV- and EFC-fed cattle growing from 381 kg to 657 kg and 387 kg to 683 kg, respectively (shown in **red**). No other inputs were changed for this simulation.

The purpose of this scenario is to evaluate the potential of Enogen feed corn to provide benefit for animals finished at a higher weight. In the backgrounding trial was observed that the main effects did not occur until later in the trial, and thus the base case Scenario 4 potentially did not capture the backgrounding benefits for the combined feeding stations. These datasets were only used in conjunction with Scenario 7, and comparative evaluation of the feed yard as a stand-alone stage was not conducted.

The original UNL observations compared to the IFSM simulated outputs are presented in Table F-3. The left side of this table reproduces the data from Johnson et al. (2018) in the right panel shows the results of simulation using the IFSM. Note that in the simulated feed yard that the silage consumption increased significantly, and the corn consumption remained constant in terms of total mass consumed but decreased as a percentage of the ration. This is not unexpected given the change in starting weight and the ration formulation heuristics of IFSM, which favor forage under circumstances where the input parameters do not constrain ration formulation.

Table F-4 presents the input and output data used in OpenLCA to calculate the lifecycle impact assessment for the scenario. Table F-5 shows the comparison between the simulated and experimental values for the total ration consumed, again highlighting the significant increase in corn silage consumption by the simulated heavier animals. Table F-6 shows, as expected, increased herd weights at the end of the simulated feed yard trial and a reduced feed conversion efficiency expressed as gain: feed, also an expected result based on known patterns of animal performance as a function of body weight.

Table F-3. UNL Feed yard: Trial and Calibrated IFSM Data

Original Nebraska study			IFSM input/output: Same LWG; FY starting weight set to BG ending weights from KSU trial, by treatment		
**Animal Performance**			**Animal Performance**		
	**Conventional**	**Enogen**		**Conventional**	**Enogen**
Initial LW (kg/head)	293	294	Initial LW (kg/head)	**380.1**	**385.6**
Final LW (kg/head)	570	590	Final LW (kg/head)	658.5	684.6
Treatment size (head)	60	60	Simulated herd size (head)	60	60
Final herd LW (kg/herd)	34,200	35,400	Final herd LW (kg/herd)	39,510	41,074
Liveweight gain (kg/head)	277	296	Liveweight gain (kg/head)	278	299
Feed consumed (kg/head)	1,776	1,793	Feed consumed (kg/head)	1,886	1,935
Days on feed	166	166	Days on feed	166	166
Average daily gain (kg/day)	1.67	1.78	Average daily gain (kg/day)	1.68	1.80
G:F	0.157	0.166	G:F	0.148	0.155
**Ration composition (% DM)**			**Ration composition (% DM)**		
	**Conventional**	**Enogen**		**Conventional**	**Enogen**
Field Corn (CNV)	64.0	0	Field Corn (CNV)	**60.3**	0.0
Enogen Feed Corn (EFC)	0	64.0	Enogen Feed Corn (EFC)	0.0	**59.3**
Silage	15	15	Silage	**20.0**	**21.4**
DDG	15	15	DDG	14.1	13.9
Supplement	6.0	6.0	Supplement	5.6	5.5
**Ration composition (kg/head)**			**Ration composition (kg/head)**		
	**Conventional**	**Enogen**		**Conventional**	**Enogen**
Field Corn	1136.8	0.0	Field Corn	1136.7	0.0
Enogen Feed Corn	0.0	1147.4	Enogen Feed Corn	0.0	1146.7
Silage	266.4	268.9	Silage	**376.7**	**413.3**
DDG	266.4	268.9	DDG	266.7	268.3
Supplement	106.6	107.6	Supplement	106.0	106.8

Table F-4. Inventory Input to Open LCA for Nebraska field trial; heavy animal

Flow	Conventional	Enogen	Unit
**Inventory: Inputs to finishing**			
**Flow**	**Conventional**	**Enogen**	**Unit**
Corn grain feed	68.2	68.8	t
Diesel	318,099	318470	MJ
DDG	16.0	16.1	t
Electricity	1,880	1,880	kWh
Silage	22.6	24.8	t
Natural gas	794	801	m3
Vitamin premix	5.8	5.8	t
Drinking water	374	384	t
**Inventory: Outputs from finishing**			
**Flow**	**Conventional**	**Enogen**	**Unit**
Ammonia; housing	935	952	kg
Ammonia; field emissions	68.4	70.6	kg
Ammonia; manure	269	268	kg
Nitrous oxide; housing	41.1	40.7	kg
Nitrous oxide; animals	5.1	5.2	kg
Nitrous oxide; manure	14.5	14.7	kg
Nitrous oxide; indirect	17.2	17.5	kg
Hydrogen sulfide; housing	6.3	6.1	kg
Methane, non-fossil; manure storage	616	626	kg
Methane, non-fossil; field emissions	3.4	3.5	kg
Methane, non-fossil; animal	1,033.9	1,075.5	kg
Methane, non-fossil; housing manure	45.3	47.4	kg
Nitrogen; leaching	98.0	100.0	kg
Nitrogen; runoff	10.0	10.0	kg
NMVOC*; housing	5.8	6.0	kg
NMVOC*; field and grazing	2.0	2.1	kg
**Product**			
**Flow**	**Conventional**	**Enogen**	**Unit**
Finished cattle	39,510	41,074	kg (LW)
Finished cattle	16,704	17,940	kg (LWG)

*Non-methane volatile organic compounds

Table F-5. IFSM simulated rations for Nebraska field trial; heavy animal

**Conventional Corn (CNV)**			
Ingredient	Study values (metric Tons)	IFSM values (metric Tons)	IFSM relative error
Corn (CNV)	68.2	68.2	-0.01%
Corn silage	15.99	22.6	**41.3%**
Distiller's grain	15.99	16	0.09%
Urea	0.639	0.6	6.1%
**Enogen Feed Corn (EFC)**			
Ingredient	Study values (metric Tons)	IFSM values (metric Tons)	IFSM relative error
Corn (EFC)	68.8	68.8	-0.06%
Corn silage	16.1	24.8	**53.7%**

Table F-6. IFSM simulated animal performance metrics: Nebraska field trial, large animal

**Conventional Corn (CNV)**			
Ingredient	*Study values*	*IFSM values*	*IFSM relative error*
Final herd liveweight (kg)	34,200	39,510	15.5%
Final animal liveweight (kg)	570	659	15.6%
Total feed consumed by the herd (kg)	106,572	113,160	6.2%
Gain to feed ratio (kg gain / kg feed intake)	0.157	0.148	-5.7%
**Enogen Feed Corn (EFC)**			
Ingredient	*Study values*	*IFSM values*	*IFSM relative error*
Final herd liveweight (kg)	35,400	41,074	16.0%
Final animal liveweight (kg)	590	685	16.1%
Total feed consumed by the herd (kg)	107,568	116,110	7.9%
Gain to feed ratio (kg gain / kg feed intake)	0.166	0.155	-6.6%

Appendix G. Cow/calf operation inventory data.

The following table presents the inventory for cow calf operations that was used in the cradle-to-harvest gate evaluation of retail cuts of meat.

Table G-1. Inventory Input to Open LCA: cow/calf phase.

Flow	Conventional	Unit
**Inventory: Inputs to cow/calf operations**		
**Flow**	**Conventional**	**Unit**
Corn grain feed	33.4	t
Corn gluten meal	16.6	MJ
Diesel	369,635	t
Electricity	24,283	kWh
Hay	11.4	t
Small grazing pasture	350	m3
Grass pasture	815	ha*yr
Pesticide	163	t
Distiller's grain	35.2	t
Vitamin premix	9.6	t
Drinking water	2,930	t
**Inventory: Outputs from cow/calf operations**		
**Flow**	**Conventional**	**Unit**
Ammonia; grazing	2,098	kg
Nitrous oxide; indirect sources	0.04	kg
Nitrous oxide; animals	49	kg
Nitrous oxide; farmland	169	kg
Hydrogen Sulfide; grazing	2.3	kg
Methane; animals	27,169	kg
Methane; field application	453	kg
Nitrogen; leaching	142	kg
NMVOC*; field and grazing	263	kg
Phosphorous; leaching	0.5	kg
Phosphorous; soluble runoff	1.1	kg
Phosphorous; sediment runoff	0.7	kg
**Product**		
Flow	**Conventional**	***Unit***
Weaned calves (ref. flow)	189	Head
Cull cattle (coproduct)	15,571	kg

*Non-methane volatile organic compounds

Appendix H. System diagrams with animal flow and impacts.

The following figures present the individual scenarios for retail cuts and for each of the gate-to-gate system boundaries for the backgrounding and feed yard stages. Each figure includes the stage-by-stage allocated impact for production of the system functional unit which is either edible cuts of beef or live weight gain. The cumulative impact for the four categories is shown in the upper right corner while in the upper left corner is the summary of animal number and weight moving through the system. The results presented for the retail cuts scenarios have been allocated based on the post-harvesting revenue generated by beef cuts, rendering products, and hides.

For the gate-to-gate scenarios, the cow-calf and harvesting unit processes will have no impact reported. On each page and particular scenario, the Enogen system is shown in the top panel and the conventional system is shown in the bottom panel. The comparative improvement results have been presented in the main document.

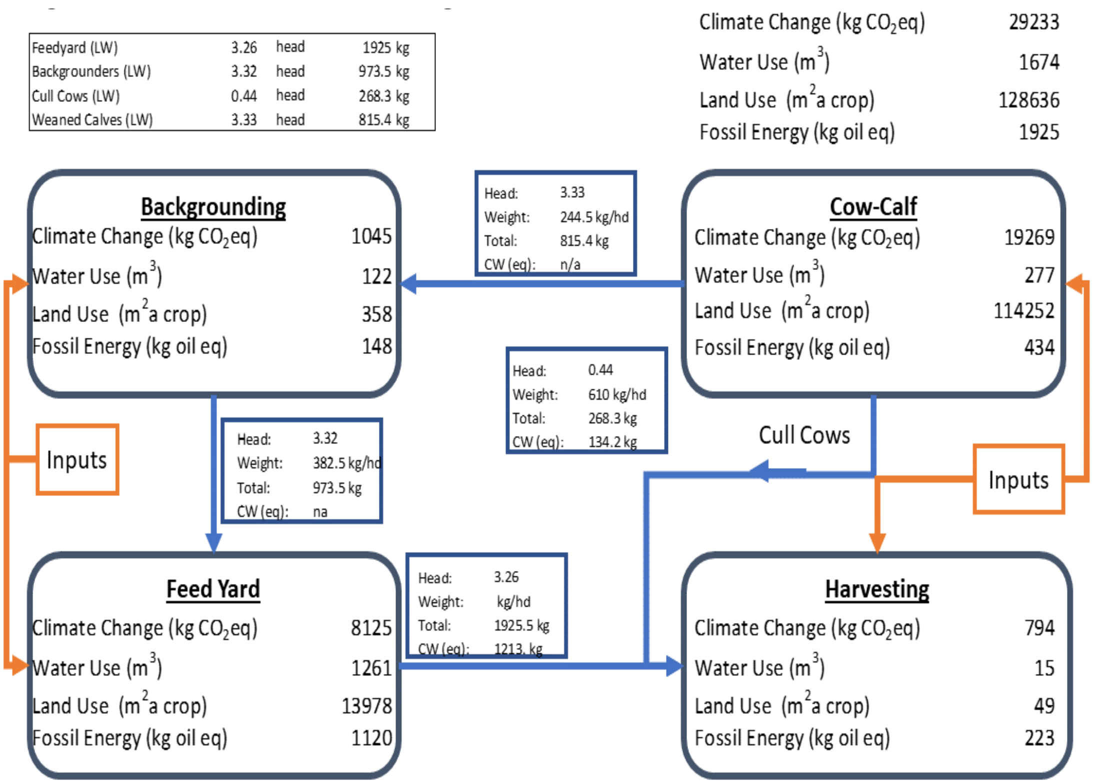

Figure H-1. Scenario 4 for retail cuts. Truncated BG; Calibrated full FY; Enogen Feed Corn

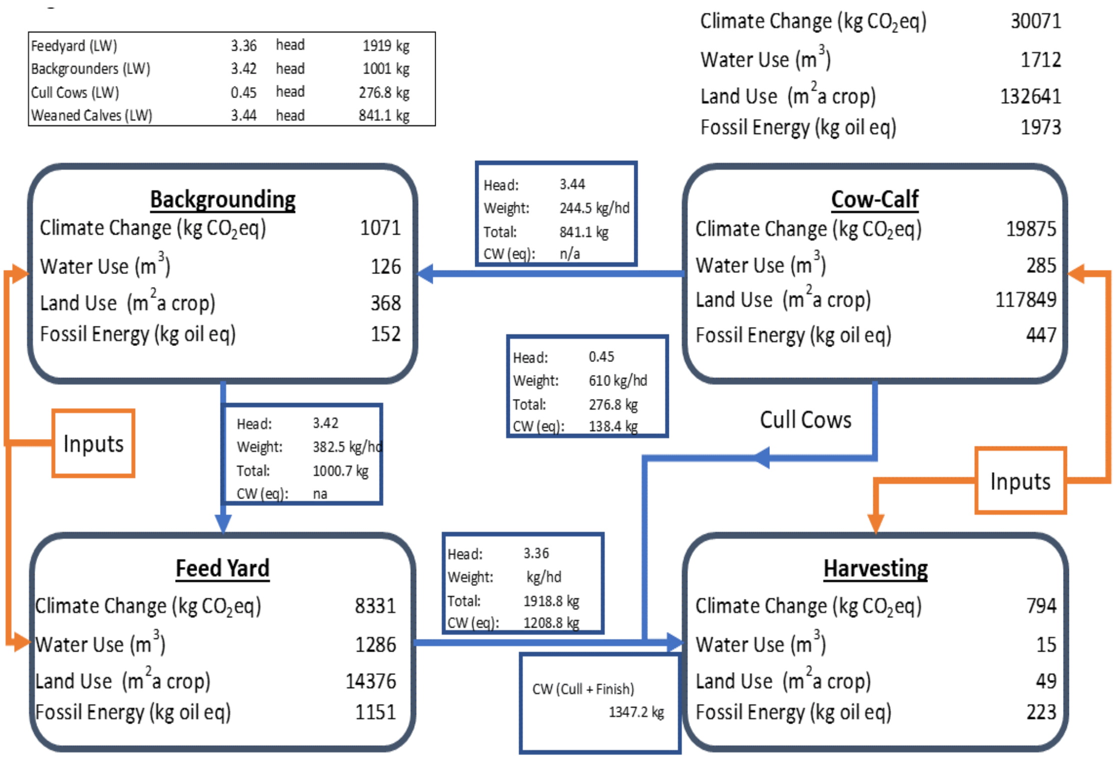

Figure H-2. Scenario 4 for retail cuts. Truncated BG; Calibrated full FY; Conventional Corn

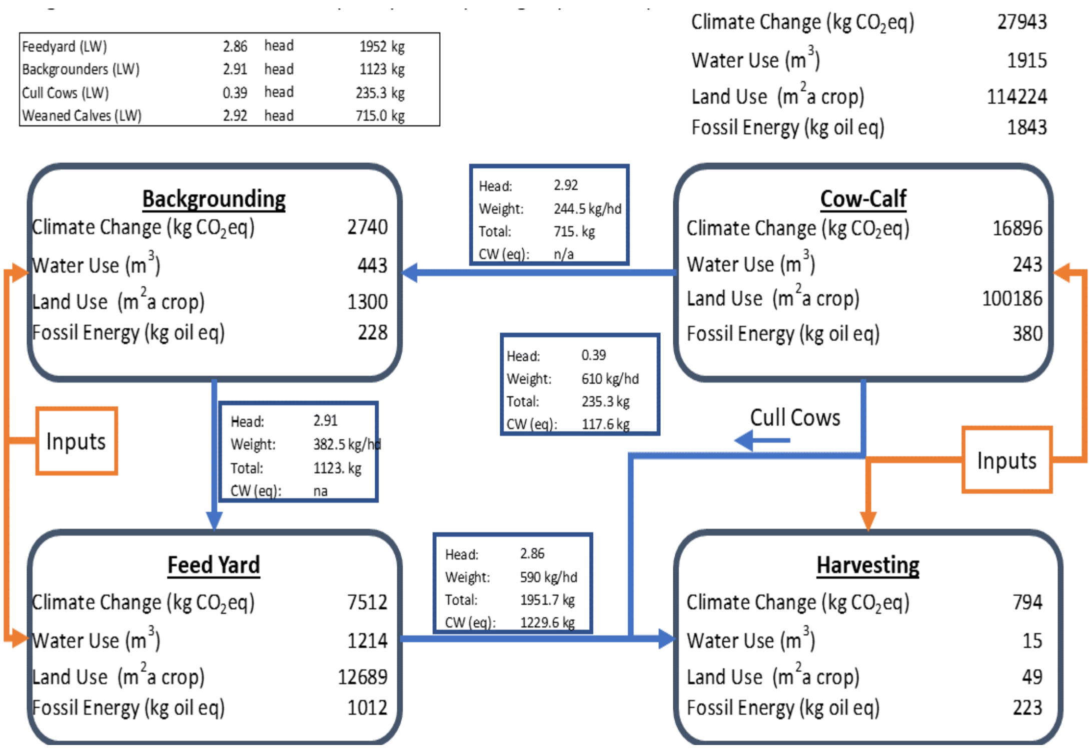

Figure H-3. Scenario 6 for retail cuts. Full BG; Full FY (matched LWG); Enogen Feed Corn,

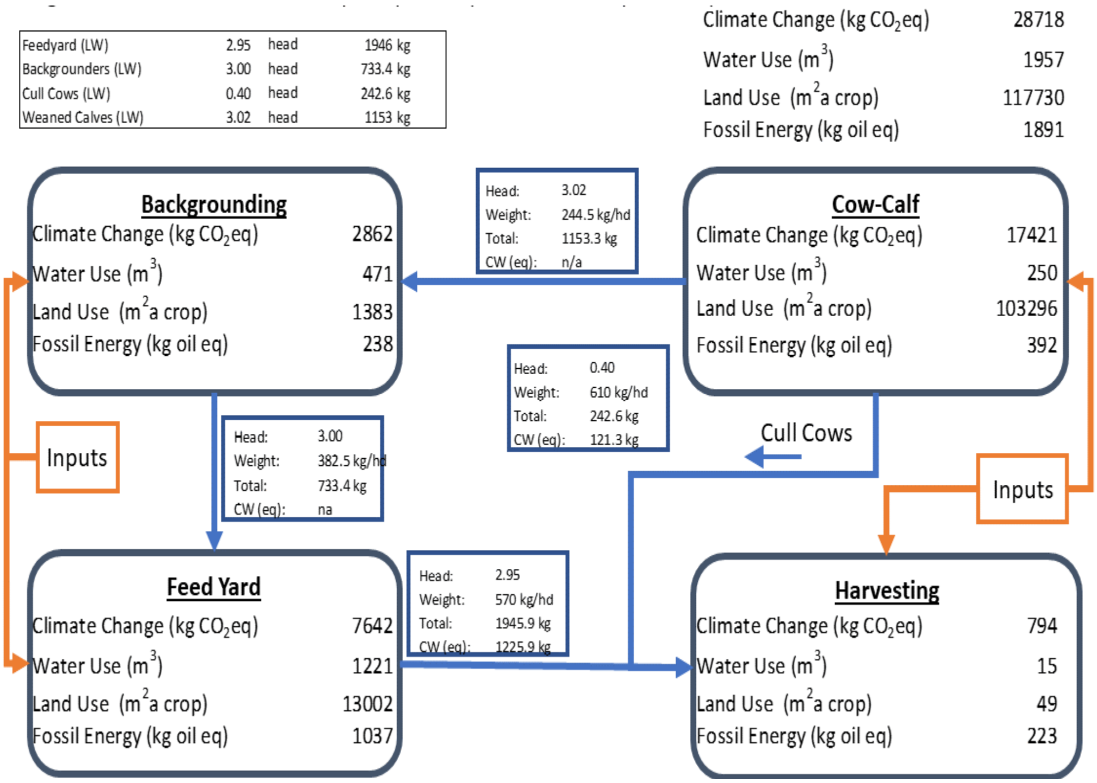

Figure H-4. Scenario 6 for retail cuts. Full background; Full FY (matched LWG); Conventional Corn

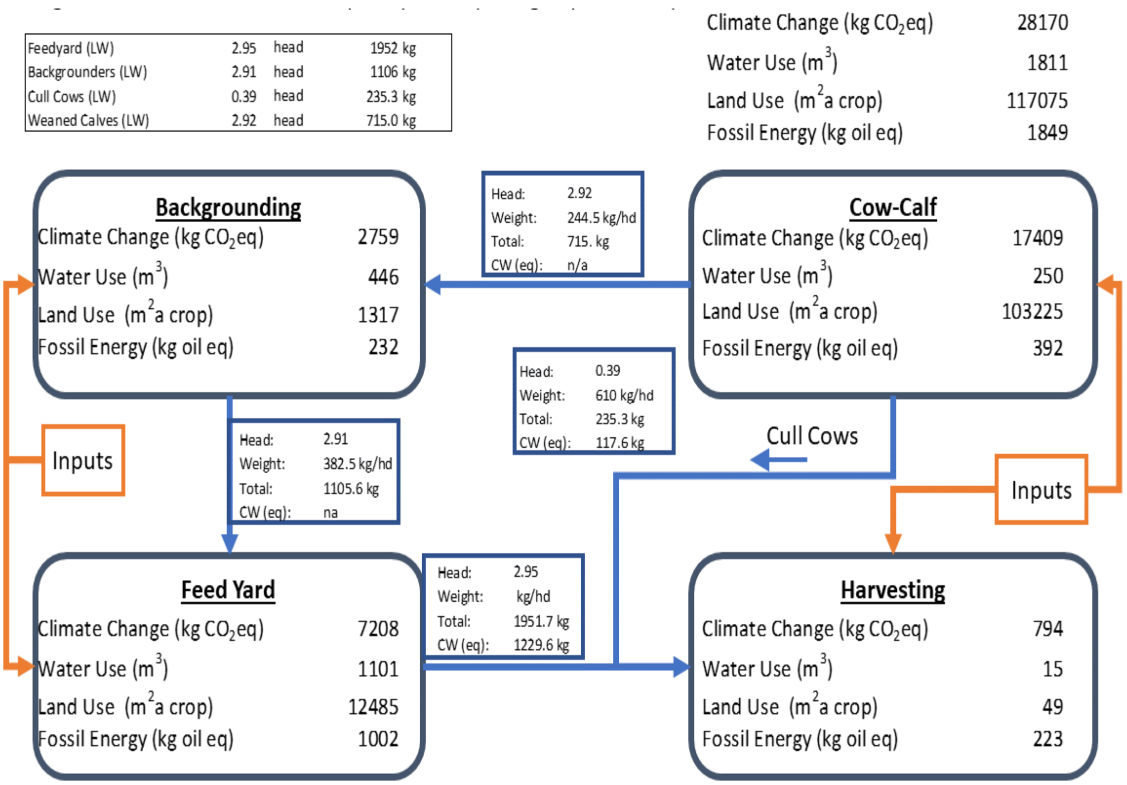

Figure H-5. Scenario 5 for retail cuts. Full BG; Full FY (paired LWG); Enogen Feed Corn, Paradigm 3

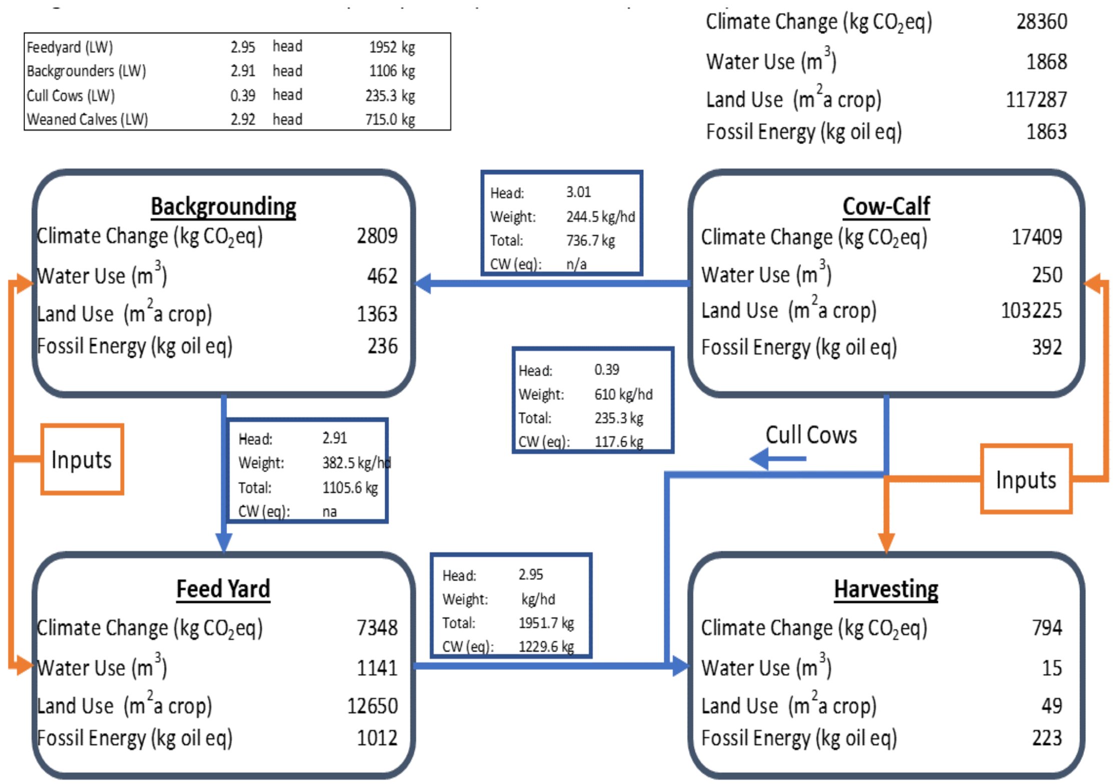

Figure H-6. Scenario 5 for retail cuts. Full background; Full UNL (paired LWG); Conventional Corn

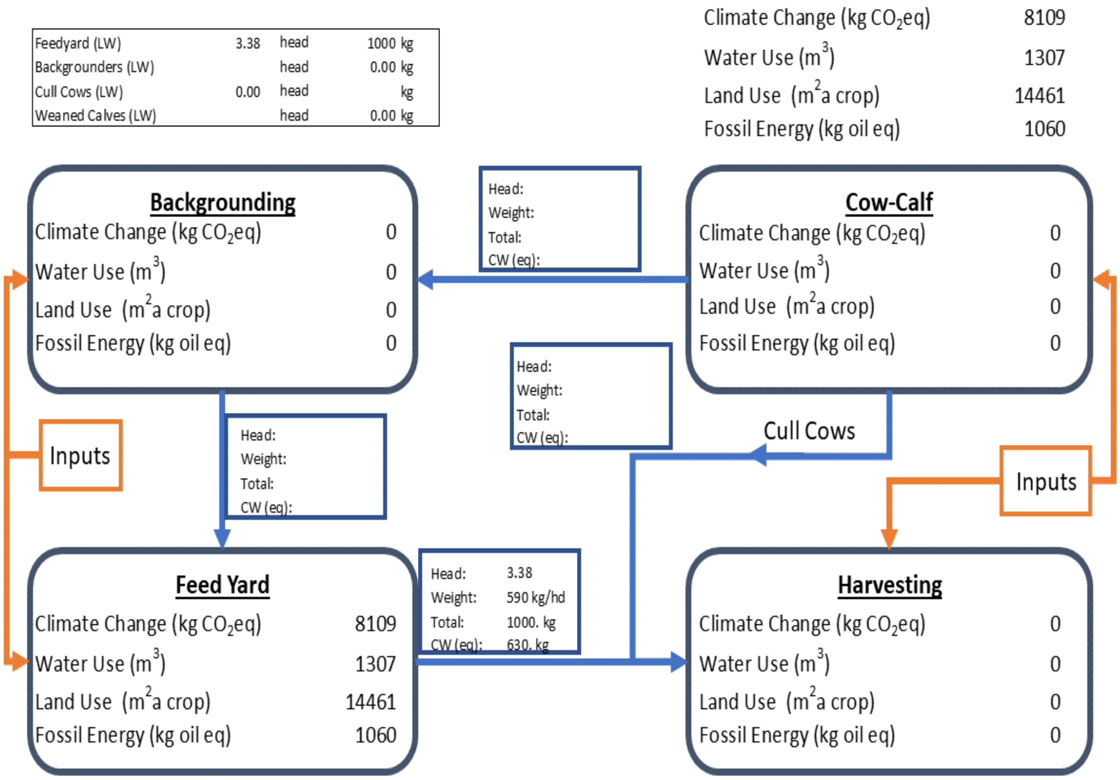

Figure H-7. Scenario 1: Calibrated UNL full trial; Enogen Feed Corn

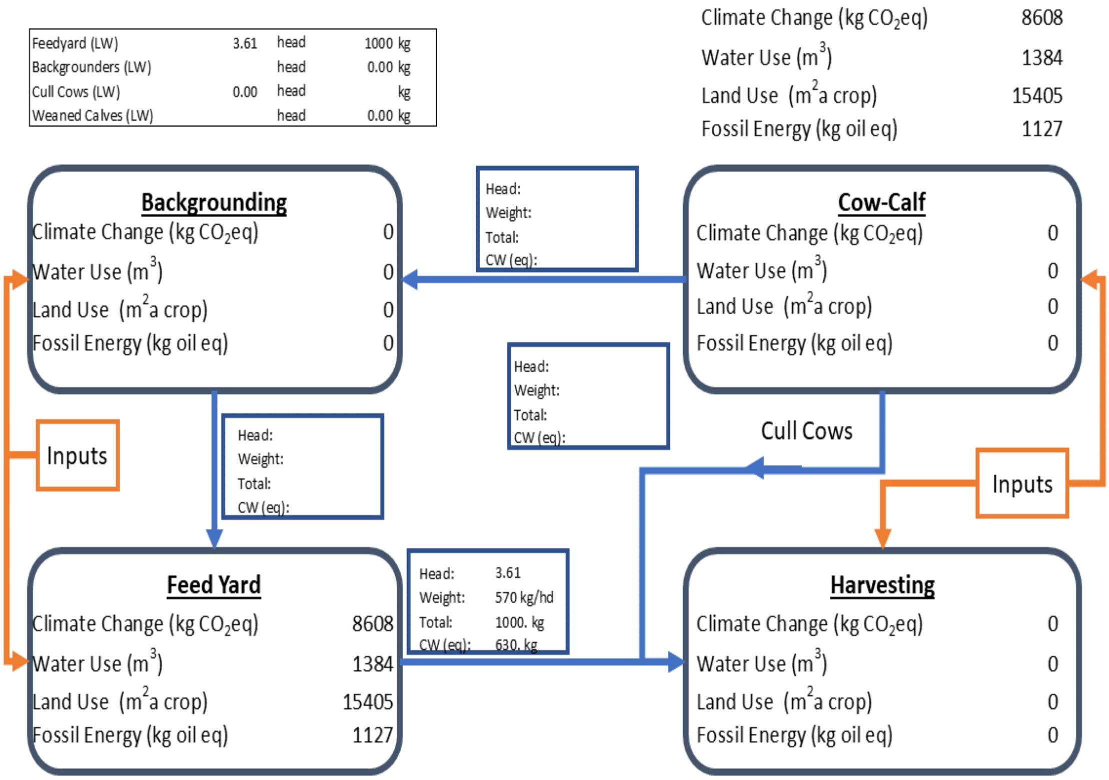

Figure H-8. Scenario 1: Calibrated UNL full trial; Conventional Corn

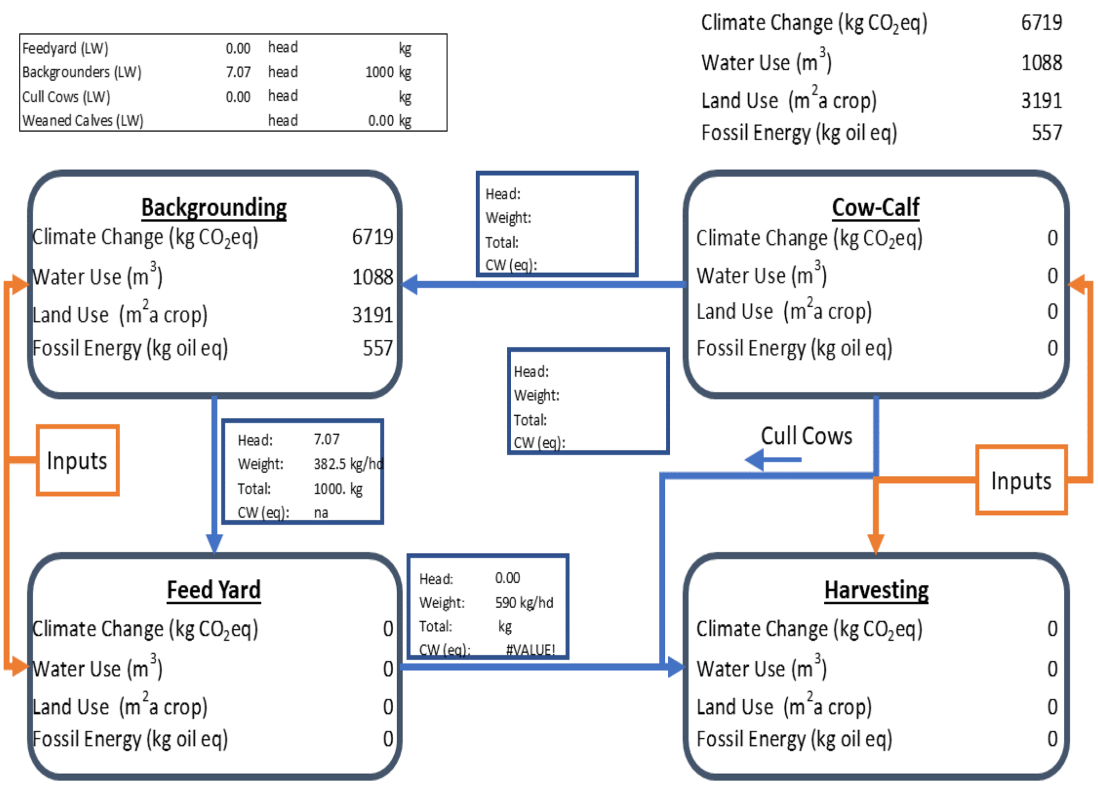

Figure H-9. Scenario 2: Calibrated KSU full backgrounding trial. Enogen Feed Corn.

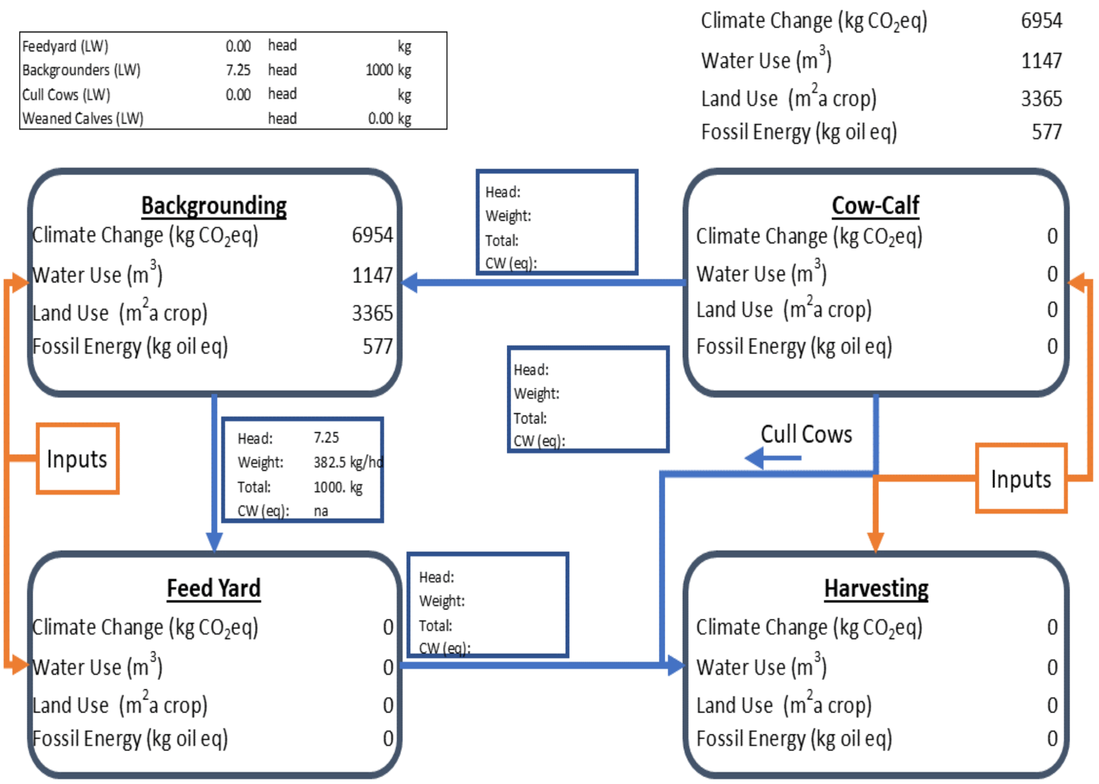

Figure H-10. Scenario 2: Calibrated KSU full backgrounding trial. Conventional Corn.

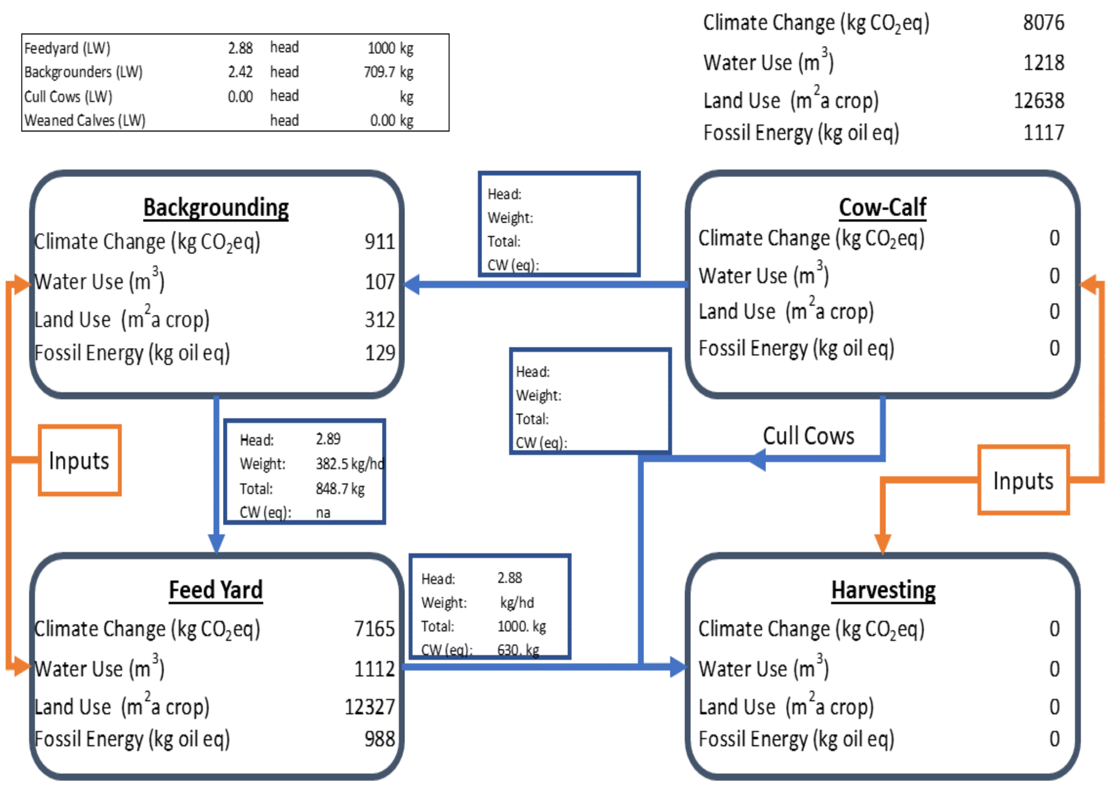

Figure H-11. Scenario 4: truncated BG plus calibrated FY yard. Enogen Feed Corn

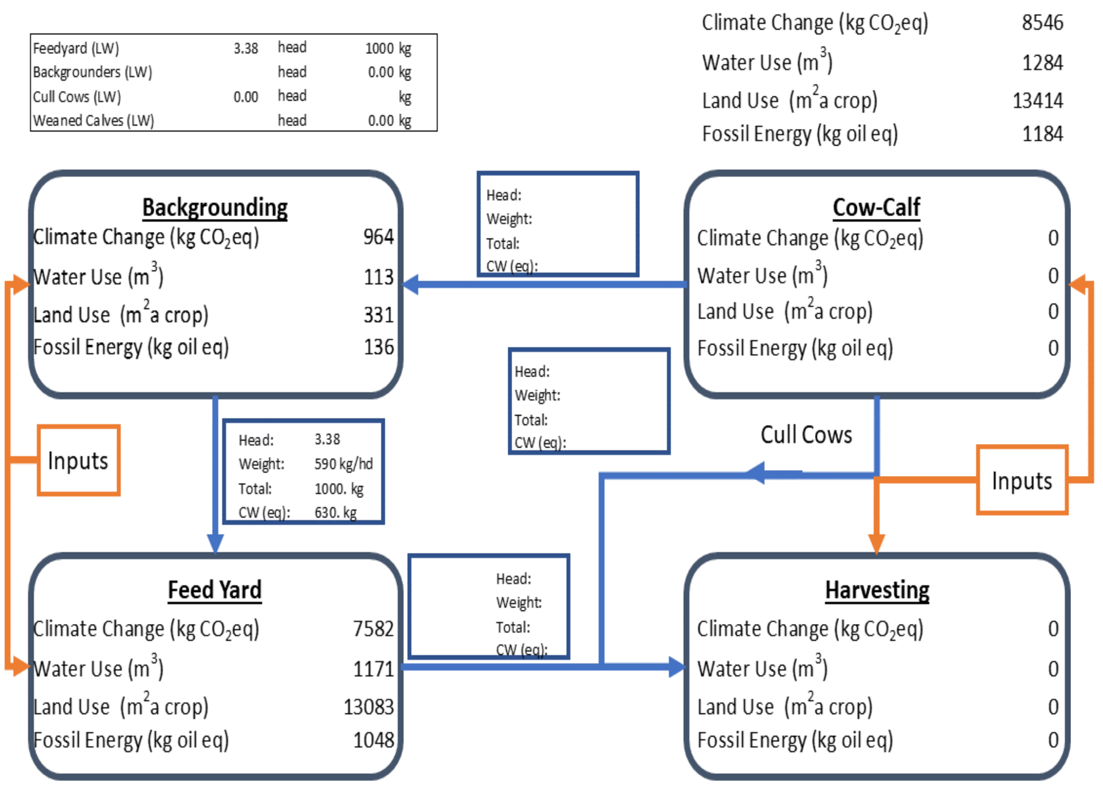

Figure H-12. Scenario 4: truncated BG plus calibrated FY. Conventional Corn

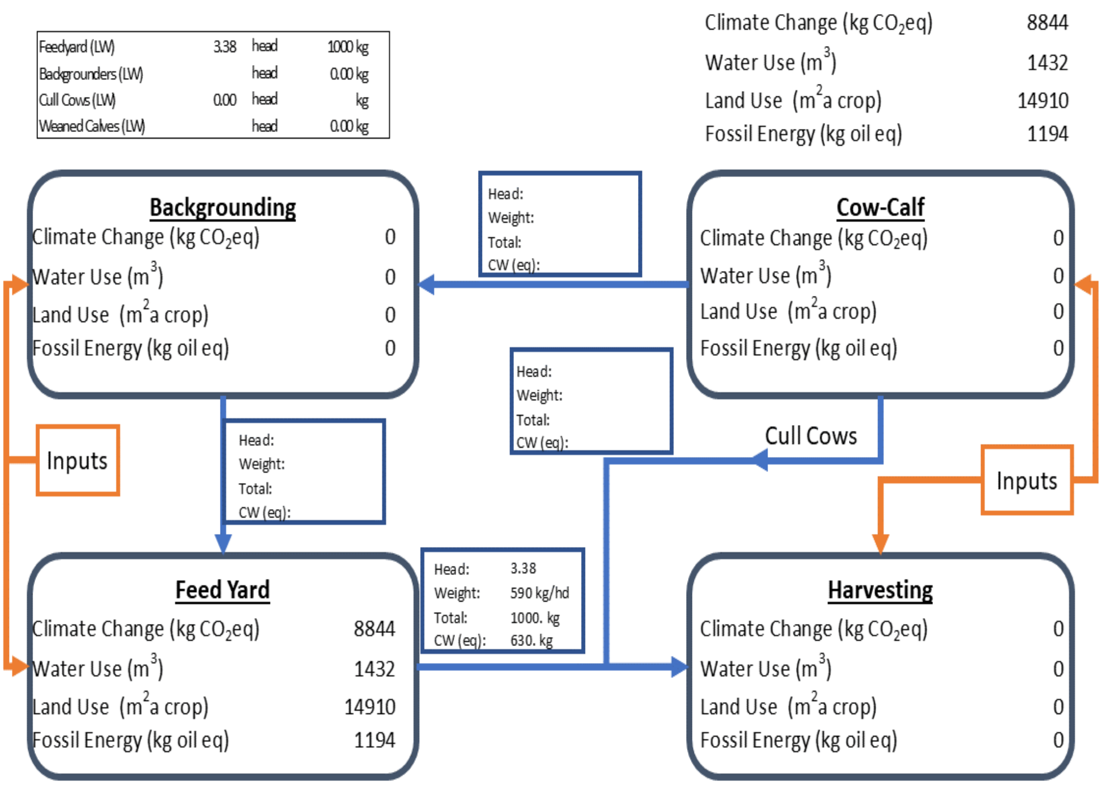

Figure H-13. Scenario 6: feed yard only; matched LWG. Enogen Feed Corn.

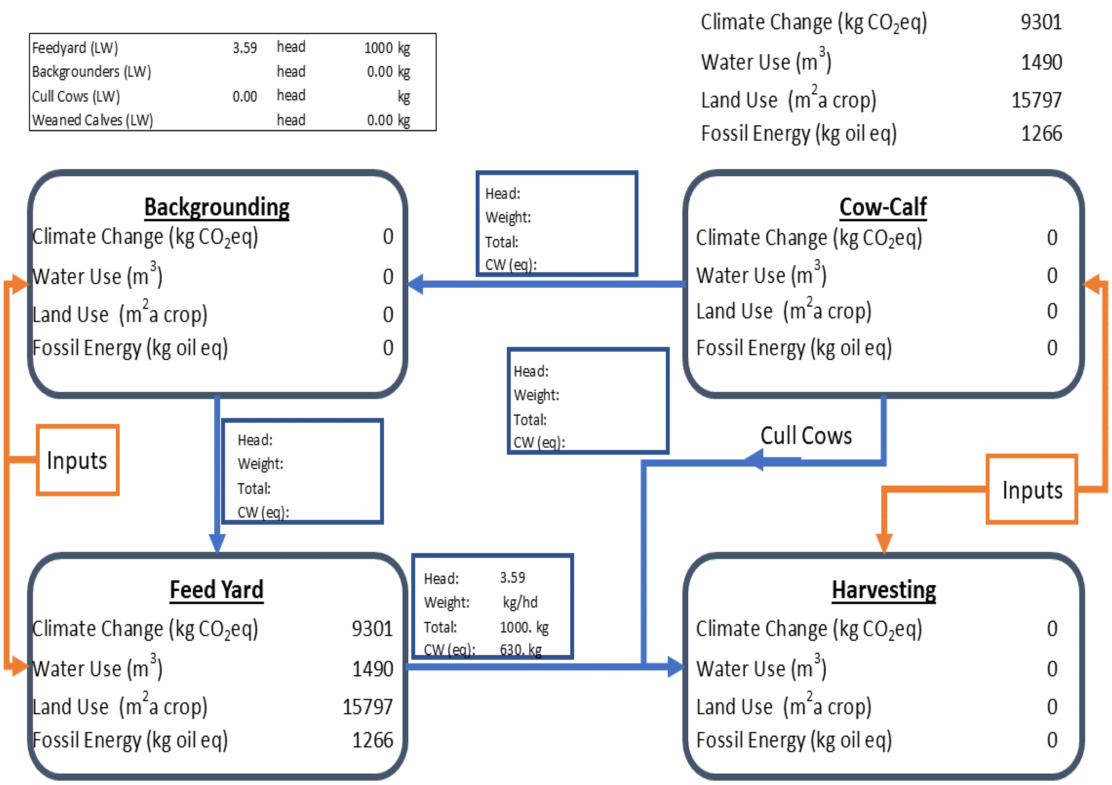

Figure H-14. Scenario 6: feed yard only; matched LWG. Conventional Corn.

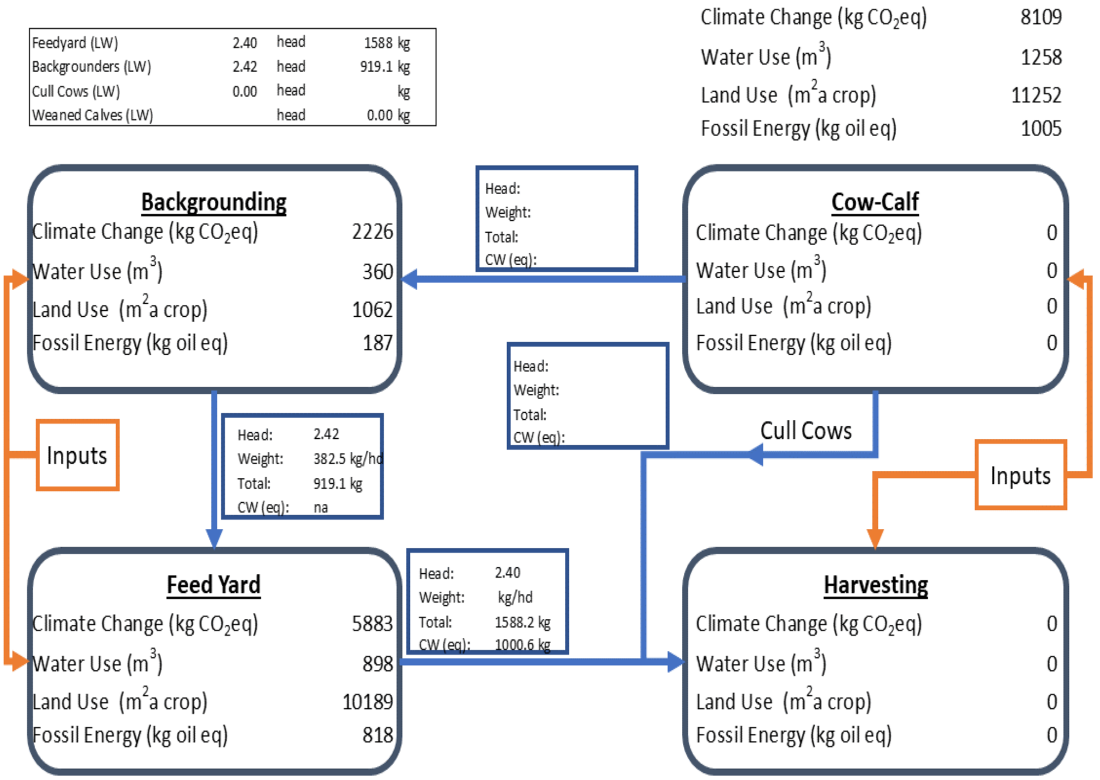

Figure H-15. Scenario 5: background plus feed yard; paired LWG. Enogen Feed Corn.

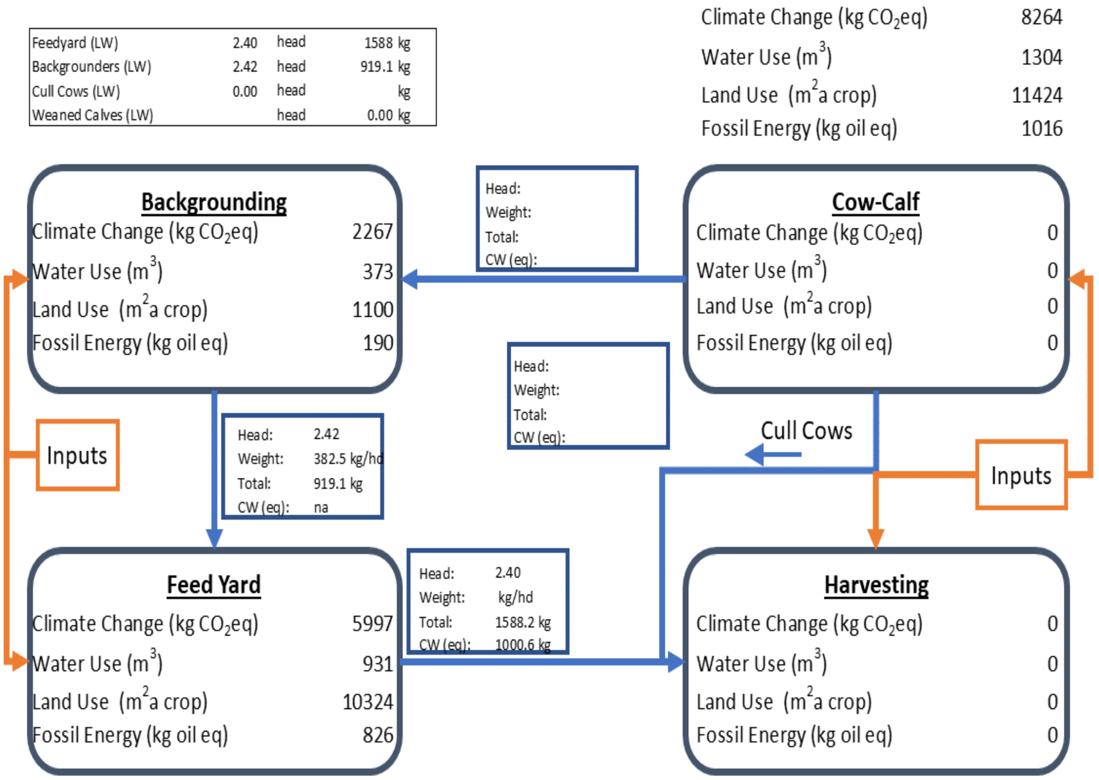

Figure H-16. Scenario 5: background plus feed yard; paired LWG. Conventional Corn.

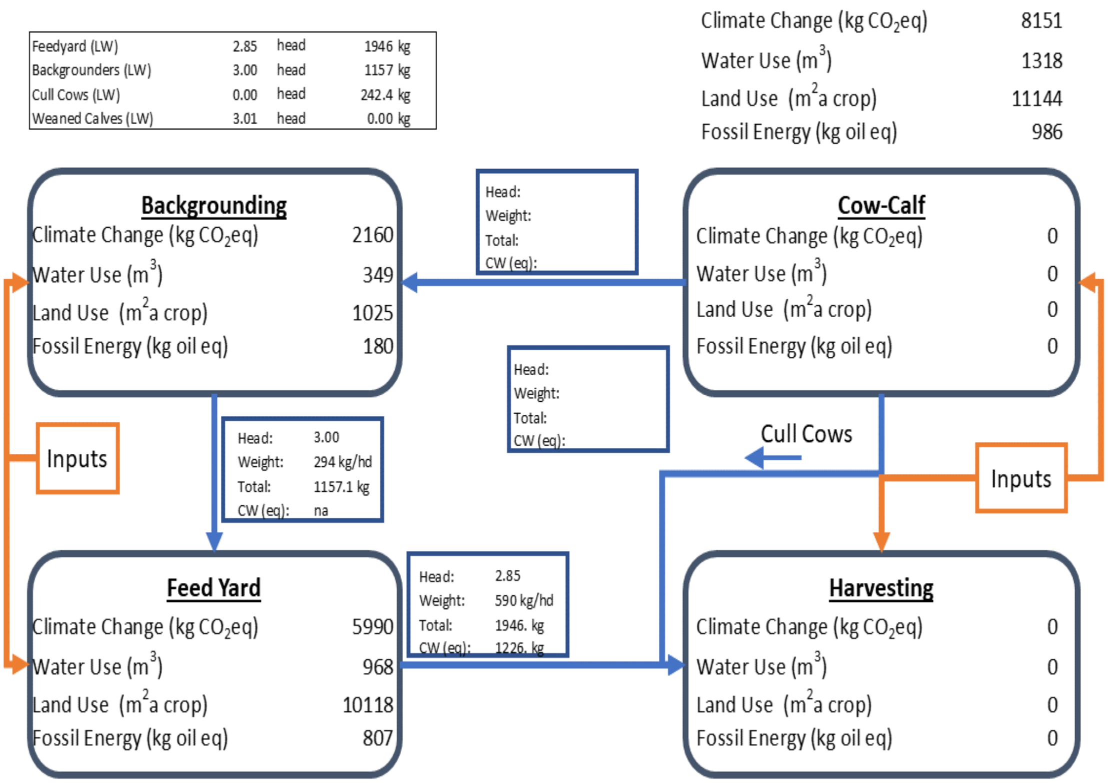

Figure H-17. Scenario 6: full BG plus FY; matched LWG yard. Enogen Feed Corn.

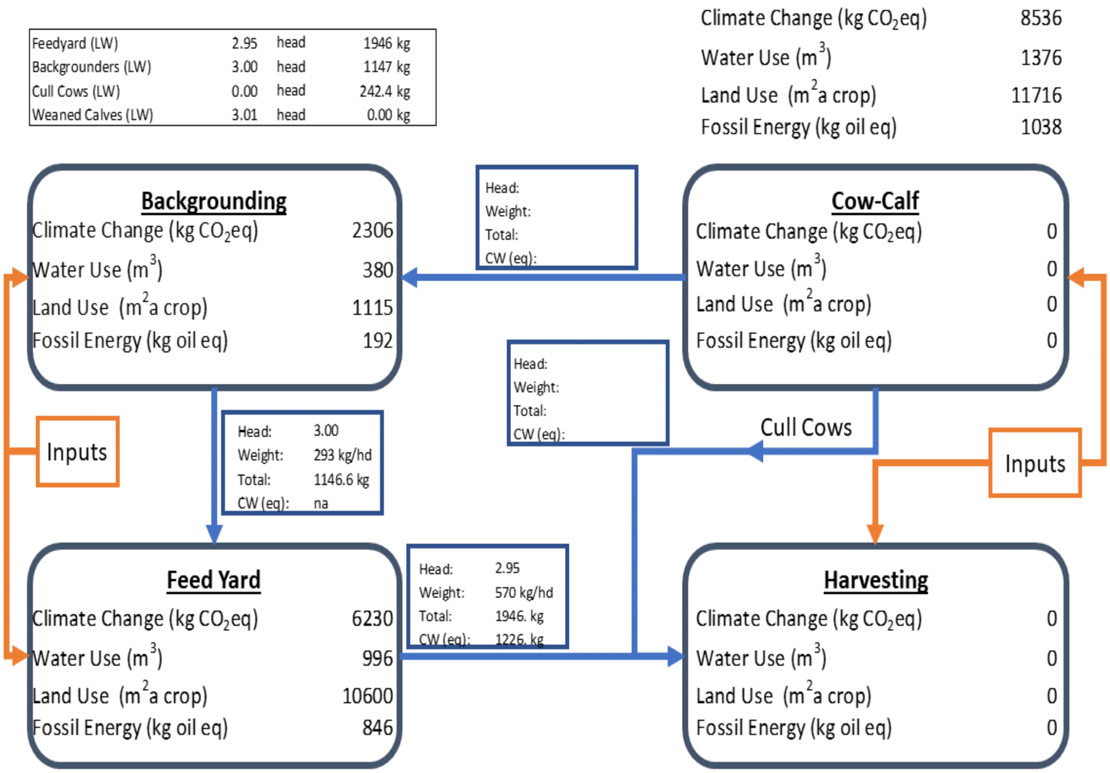

Figure H-18. Scenario 6: full BG plus FY; matched LWG yard. Conventional Corn.

Appendix I. Bootstrap Monte Carlo simulation statistical significance testing.

Each input parameter to the open LCA lifecycle inventory model was assigned a probability distribution based on either a) the mean and standard deviation reported from the 25 year simulation using the Integrated Farm System Model, or B) when the coefficient of variation was unreasonably small from the IFSM simulation, we assigned a distribution with a coefficient of variation of 5% for input parameters and 10% for emissions to the environment. These were incorporated as lognormal distributions to avoid the possibility of selecting a negative result for positive-definite parameters. Each scenario was simulated by selecting random variance from the probability distribution function for each input parameter using the open LCA software platform and the lifecycle impact assessment calculated for 250 Monte Carlo runs.

The open LCA platform returns a difference from a constructed unit process with an input of 1,000 kg from the conventional corn-based ration and a -1,000 kg input from the Enogen Feed corn simulation. The bootstrap technique involved selecting a random difference from the Monte Carlo simulation population 30 times, with replacement. This sub- sample of the Monte Carlo runs was then evaluated using a one tailed Student's t-test and a T-value calculated to determine whether the null hypothesis that CNV equals EFC should be rejected in favor of the alternate hypothesis CNV greater than EFC.

Table I-1 presents the results of the bootstrap evaluation. In that table, green highlighted cells indicate for the scenario and impact combination that CNV > EFC ($p<0.01$). Tabulated values are the upper 95% confidence interval for the distribution of p-values from the bootstrap sampling of 250 Monte Carlo runs.

Table I-1. Bootstrap Monte Carlo results. Green cells indicate p-value less than 0.01; salmon color cells indicate p-value greater than 0.01

**Scenario (#)**	Global warming	Water consumption	Land use	Fossil resource scarcity	Freshwater eutrophication	Marine eutrophication	Fine particulate matter formation	Freshwater ecotoxicity	Mineral resource scarcity
UNL calibration (1)	2.3E-08	6.6E-05	2.1E-18	7.5E-09	2.1E-07	2.8E-05	1.2E-04	1.4E-07	9.7E-08
KSU calibration (2)	4.8E-03	5.9E-06	6.6E-07	4.9E-05	4.6E-07	7.5E-03	1.6E-02	4.3E-06	3.7E-07
BGFY (4)	4.1E-04	2.7E-02	8.8E-04	4.0E-04	2.2E-03	1.2E-01	6.4E-06	2.1E-03	2.1E-03
Cradle-to-gate (4)	0.0E+00	4.2E-04	0.0E+00	0.0E+00	0.0E+00	3.6E-01	0.0E+00	1.4E-09	3.5E-17
BGFY (5)	9.8E-03	6.2E-04	2.3E-04	7.8E-02	2.2E-03	5.8E-04	3.0E-02	2.0E-02	4.8E-03
Retail (5)	1.4E-01	1.2E-03	1.5E-03	6.6E-02	6.9E-03	2.4E-02	1.2E-01	9.9E-02	1.2E-02
BGFY (6)	5.6E-08	2.2E-05	2.1E-15	3.0E-07	6.2E-09	1.4E-04	1.3E-04	2.9E-08	5.1E-08
Retail (6)	0.0E+00	3.3E-09	0.0E+00	0.0E+00	0.0E+00	8.3E-02	3.1E-12	2.4E-17	0.0E+00
FY (6)	7.4E-06	3.1E-03	1.4E-15	4.1E-09	8.8E-07	1.1E-03	7.8E-05	3.1E-08	1.6E-07

**Scenario**	Marine ecotoxicity	Human carcinogenic toxicity	Human non-carcinogenic toxicity	Ozone formation, Human health	Ozone formation ecosystems	Stratospheric ozone depletion	Terrestrial acidification	Terrestrial ecotoxicity	Ionizing radiation
UNL calibration (1)	3.9E-08	2.7E-06	6.8E-07	5.5E-06	1.4E-05	6.6E-08	1.6E-03	3.7E-08	1.8E-05
KSU calibration (2)	1.7E-06	9.4E-05	1.1E-05	2.1E-02	1.9E-02	3.0E-02	1.7E-02	2.5E-06	1.9E-04
BGFY (4)	5.0E-03	5.5E-03	2.6E-03	5.1E-04	8.5E-05	2.2E-04	4.6E-05	2.8E-03	1.2E-03
Cradle-to-gate (4)	2.9E-09	1.2E-08	3.6E-11	0.0E+00	0.0E+00	0.0E+00	0.0E+00	4.0E-18	2.4E-10
BGFY (5)	1.8E-02	1.7E-01	2.1E-02	4.8E-01	4.7E-01	4.4E-03	3.3E-02	1.2E-02	1.9E-01
Retail (5)	1.0E-01	1.9E-01	1.4E-01	4.5E-01	4.3E-01	1.4E-01	1.5E-01	9.1E-03	7.0E-02
BGFY (6)	5.8E-08	1.2E-05	6.2E-08	3.8E-05	2.4E-05	1.4E-06	1.1E-03	6.4E-07	2.2E-06
Retail (6)	3.7E-17	8.7E-09	2.0E-15	0.0E+00	0.0E+00	2.3E-08	4.4E-09	0.0E+00	7.0E-13
FY (6)	7.6E-08	3.1E-03	1.4E-07	1.9E-05	6.8E-06	2.6E-05	2.9E-03	3.5E-07	1.1E-07

Appendix J. Contribution analysis.

This section presents the full array of midpoint impact categories from the ReCiPe 2016 (H) impact method. The contribution analysis is based on an assignment of each of the unit processes in the supply chain to a class of activity as shown in the legend. It should be noted that due to the focus of the project on four main categories that inventory for each of additional these categories was not explicitly included. For example, specific pesticides used in cattle operations were adopted as generic pesticides based on the simulation. Dust and particulate matter associated with feedlot operations were not included in the analysis.

Table J-1. Lifecycle impact assessment results comparing CNV and EFC ration treatments across all scenarios.

Scenario	Land use m2a crop eq	Global warming kg CO2 eq	Terrestrial ecotoxicity kg 1,4-DCB	Human non-carcinogenic toxicity kg 1,4-DCB	Fossil resource scarcity kg oil eq	Water consumption m3	Terrestrial acidification kg SO2 eq	Ionizing radiation kBq Co-60 eq	Human carcinogenic toxicity kg 1,4-DCB
Scenario 1: FY, CNV	15,405	8,608	5,764	1,342	1,127	1,384	152	76.1	74.7
Scenario 1: FY, EFC	14,461	8,109	5,432	1,265	1,060	1,307	141	71.6	70.2
Scenario 2: BG, CNV	3,365	6,954	2,794	760	577	1,147	129	45.2	42.4
Scenario 2: FY, EFC	3,191	6,719	2,675	727	557	1,088	120	43.6	40.9
Scenario 3: Truncated CNV	13,414	8,546	5,581	1,266	1,184	1,284	144	75.2	76.7
Scenario 3: Truncated EFC	12,638	8,076	5,268	1,197	1,117	1,218	134	71	72.3
Scenario 4: Retail Cut, CNV	132,641	30,071	9,667	3,440	1,973	1,712	242	211	178
Scenario 4: Retail Cut, EFC	128,636	29,233	9,446	3,380	1,925	1,674	234	207	174
Scenario 5: BGFY Paired LWG, EFC	11,442	8,286	4,980	1,177	1,021	1,310	146	69	68.3
Scenario 5: BGFY Paired LWG, CNV	11,269	8,131	4,877	1,149	1,010	1,264	140	67.9	67.3
Scenario 5: Retail Cut, EFC	117,308	28,387	9,391	3,388	1,869	1,875	253	205	171
Scenario 5: Retail Cut, CNV	117,261	28,337	9,364	3,381	1,865	1,859	251	204	171
Scenario 6: BGFY, Match LWG, CNV	11,733	8,559	5,173	1,225	1,043	1,381	155	70.9	70
Scenario 6: BGFY, Match LWG, EFC	11,160	8,172	4,938	1,171	991	1,323	146	67.6	66.6
Scenario 6: FY, CNV	15,797	9,301	6,282	1,445	1,266	1,490	167	83.1	83.1
Scenario 6: FY, EFC	14,910	8,844	5,966	1,377	1,194	1,432	158	78.7	78.6
Scenario 6: Retail Cut, EFC	117,730	28,718	9,593	3,440	1,891	1,957	264	206	173
Scenario 6: Retail Cut, CNV	114,224	27,943	9,387	3,384	1,843	1,915	255	202	169

Scenario	Marine ecotoxicity kg 1,4-DCB	Freshwater ecotoxicity kg 1,4-DCB	Ozone formation, Terrestrial ecosystems kg NOx eq	Ozone formation, Human health kg NOx eq	Fine particulate matter formation kg PM2.5 eq	Mineral resource scarcity kg Cu eq	Marine eutrophication kg N eq	Freshwater eutrophication kg P eq	Stratospheric ozone depletion kg CFC11 eq
Scenario 1: FY, CNV	62.6	42.8	24.4	23.9	24.5	9.51	3.01	0.764	0.076
Scenario 1: FY, EFC	59	40.3	22.9	22.4	22.8	8.95	2.83	0.719	0.071
Scenario 2: BG, CNV	35.8	24.9	12.5	12.2	18.9	5.22	2.01	0.501	0.066
Scenario 2: FY, EFC	34.3	23.9	12.2	11.9	17.7	4.99	1.9	0.481	0.063
Scenario 3: Truncated CNV	59.2	40.2	28.4	27.9	24	9.36	2.72	0.713	0.071
Scenario 3: Truncated EFC	56	38	26.7	26.2	22.4	8.84	2.57	0.674	0.067
Scenario 4: Retail Cut, CNV	131	91.4	44.7	43.4	41.5	18.3	3.95	1.92	0.13
Scenario 4: Retail Cut, EFC	128	89.4	43.5	42.2	40.1	17.9	3.84	1.87	0.126
Scenario 5: BGFY Paired LWG, EFC	55.2	37.8	23.2	22.8	23.3	8.47	2.71	0.689	0.073
Scenario 5: BGFY Paired LWG, CNV	53.9	36.9	23.1	22.7	22.5	8.28	2.6	0.673	0.071
Scenario 5: Retail Cut, EFC	128	90.1	40.7	39.5	42.1	17.9	4.17	1.88	0.135
Scenario 5: Retail Cut, CNV	128	89.8	40.7	39.5	41.8	17.9	4.13	1.88	0.134
Scenario 6: BGFY, Match LWG, CNV	57.3	39.3	23.5	23	24.5	8.78	2.84	0.715	0.075
Scenario 6: BGFY, Match LWG, EFC	54.7	37.5	22.2	21.8	23.1	8.39	2.71	0.684	0.071
Scenario 6: FY, CNV	67.5	46.1	28.7	28.2	27.1	10.5	3.23	0.817	0.079
Scenario 6: FY, EFC	64.3	43.9	26.9	26.4	25.6	9.97	3.09	0.779	0.074
Scenario 6: Retail Cut, EFC	130	91.3	41	39.8	43.5	18.2	4.3	1.9	0.137
Scenario 6: Retail Cut, CNV	128	89.5	39.8	38.6	42.1	17.9	4.2	1.86	0.133

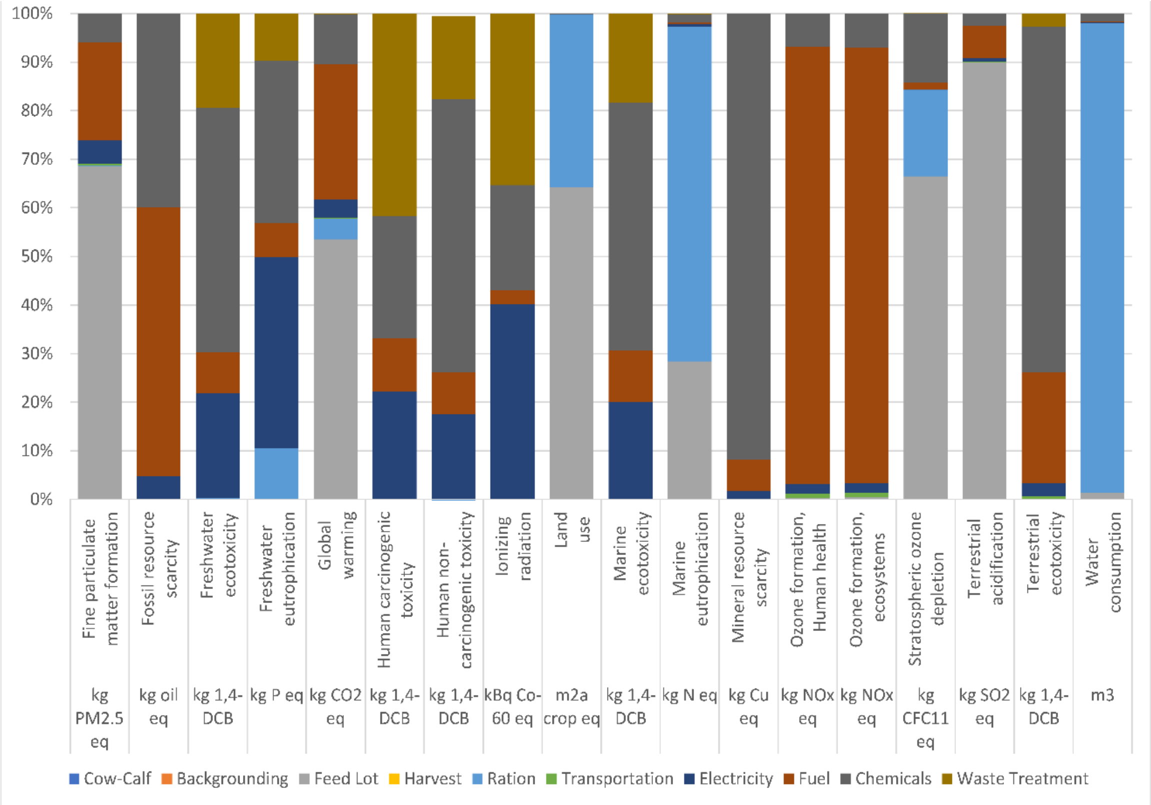

Figure J-1. Scenario 1: UNL Feed Yard Calibrated Conventional Corn

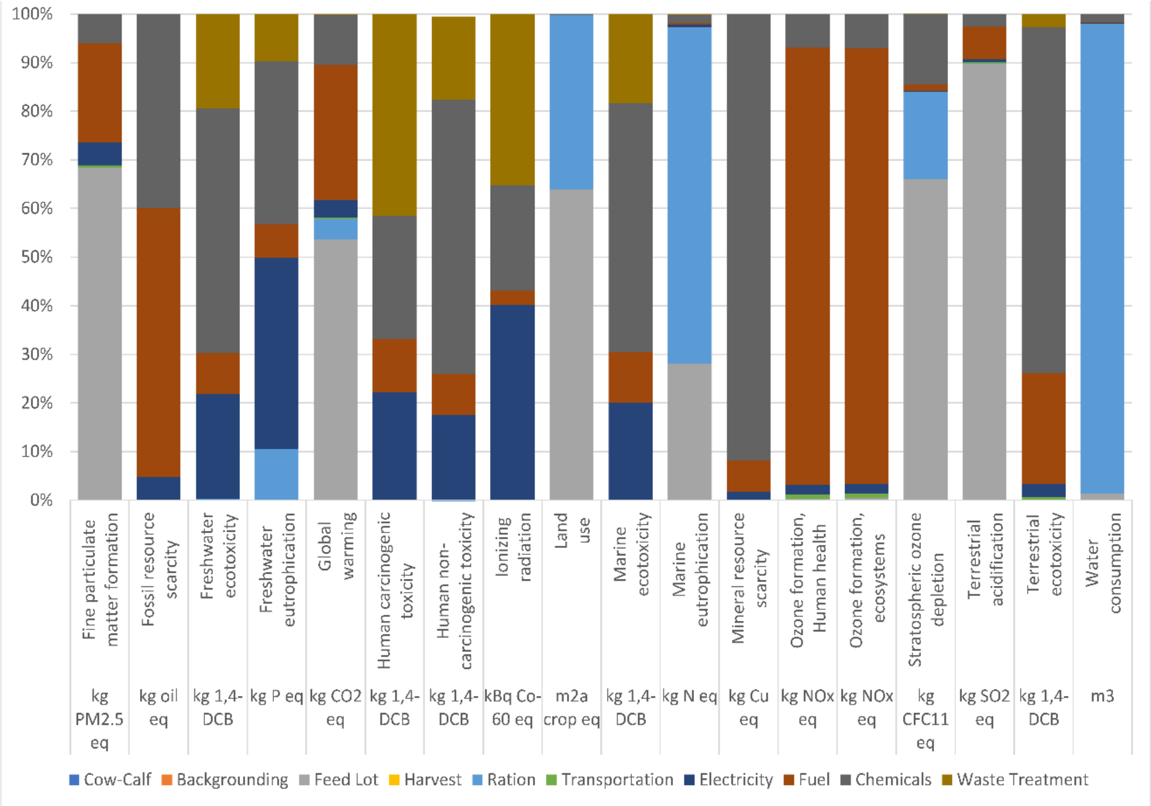

Figure J-2. Scenario 1: UNL Feed Yard Calibrated Enogen Feed Corn

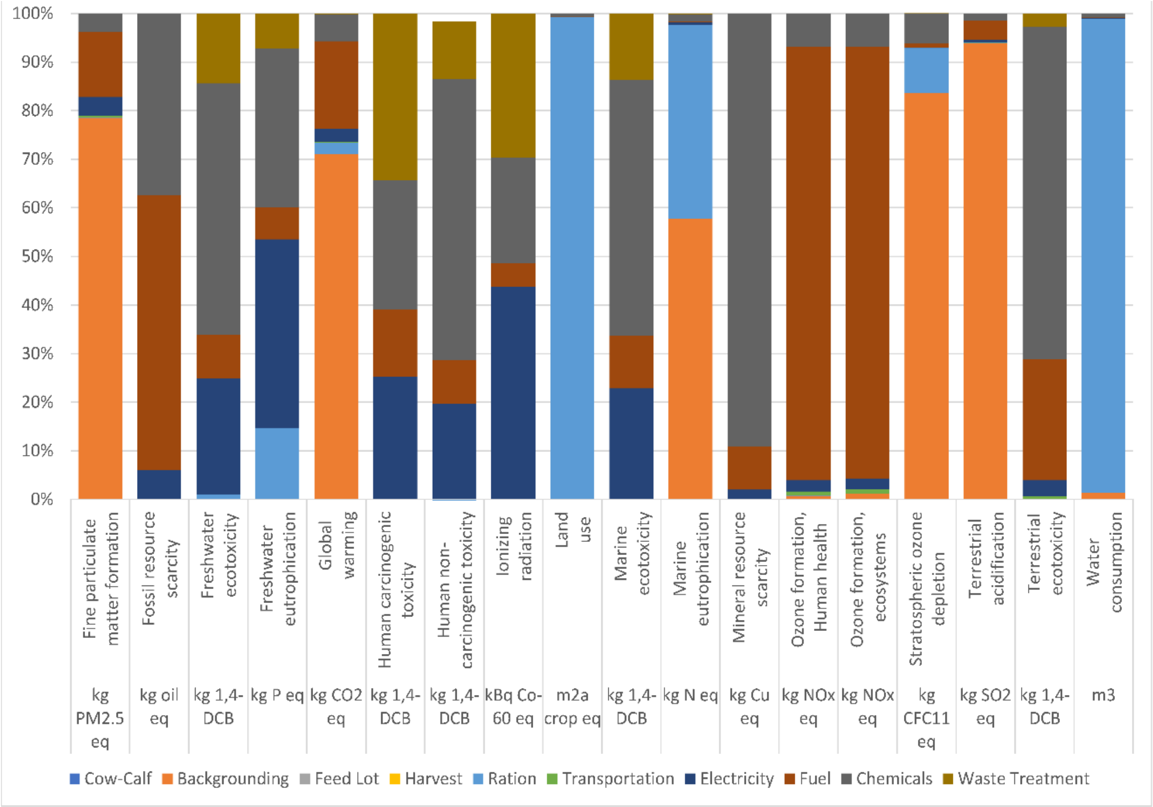

Figure J-3. Scenario 2: KSU Full Background Calibrated Conventional Corn

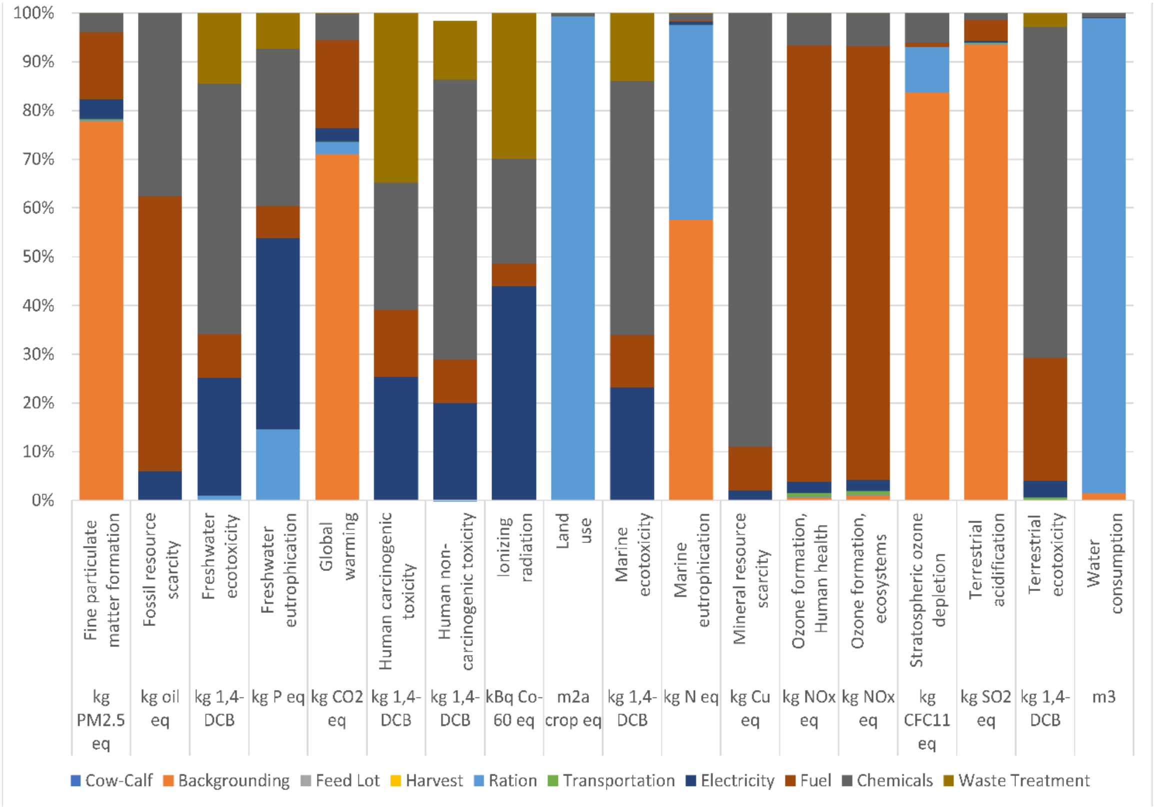

Figure J-4. Scenario 2: KSU Full Background Calibrated Enogen Feed Corn

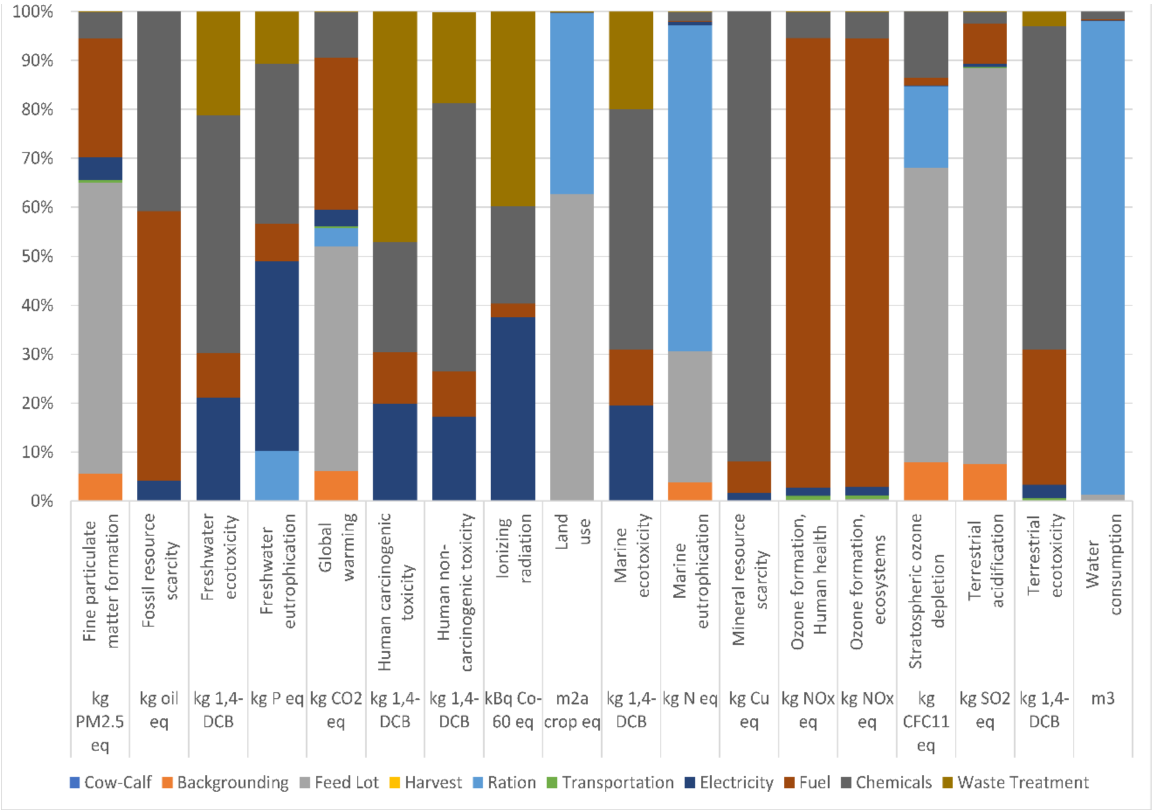

Figure J-5. Scenario 4: Background (truncated) plus Feed Yard GtG Conventional Corn

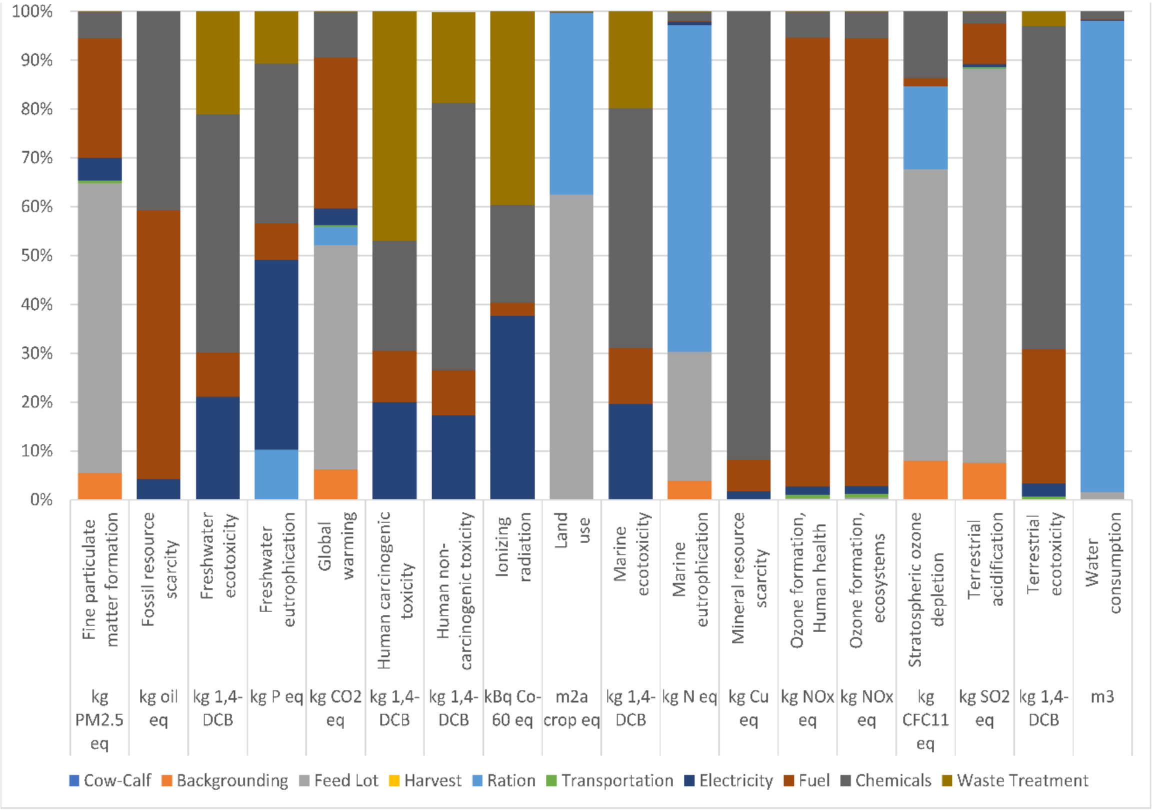

Figure J-6. Scenario 4: Background (truncated) plus Feed Yard GtG Enogen Feed Corn

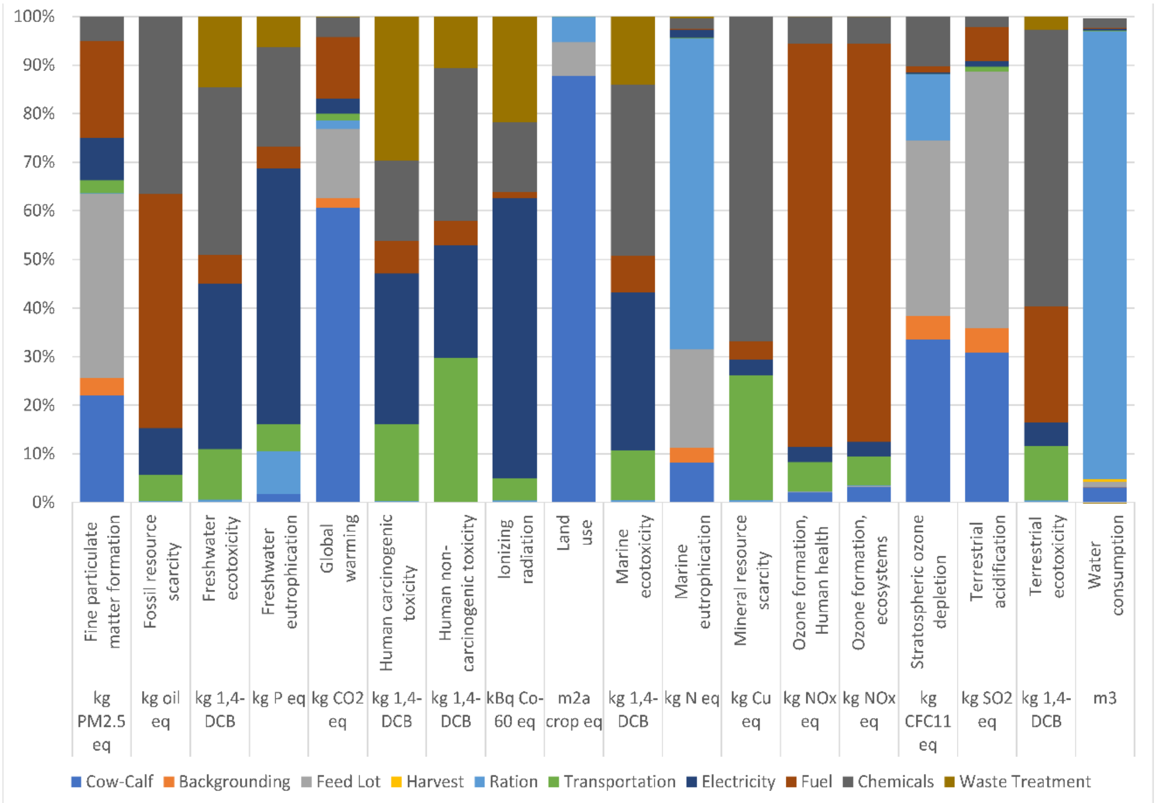

Figure J-7. Scenario 4: Cradle-to-Retail Cut; Truncated BG; Full FY Conventional Corn

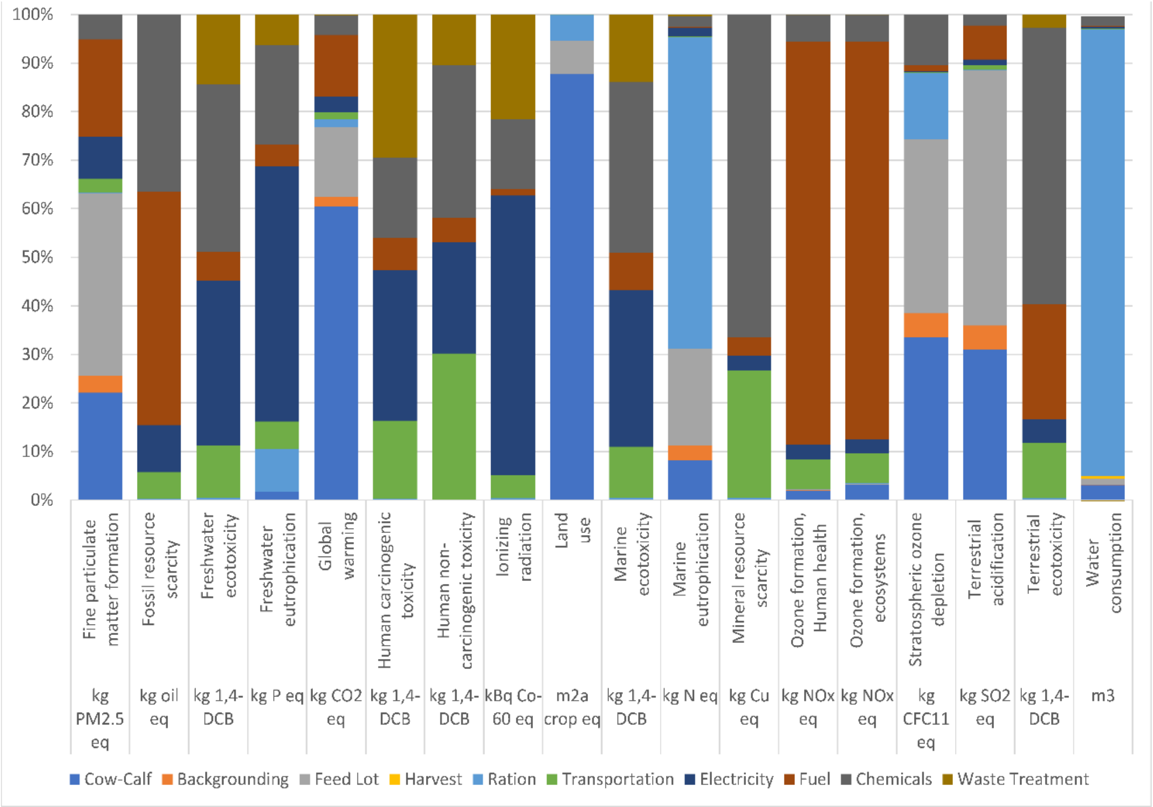

Figure J-8. Scenario 4: Cradle-to-Retail Cut; Truncated BG; Full FY Enogen Feed Corn

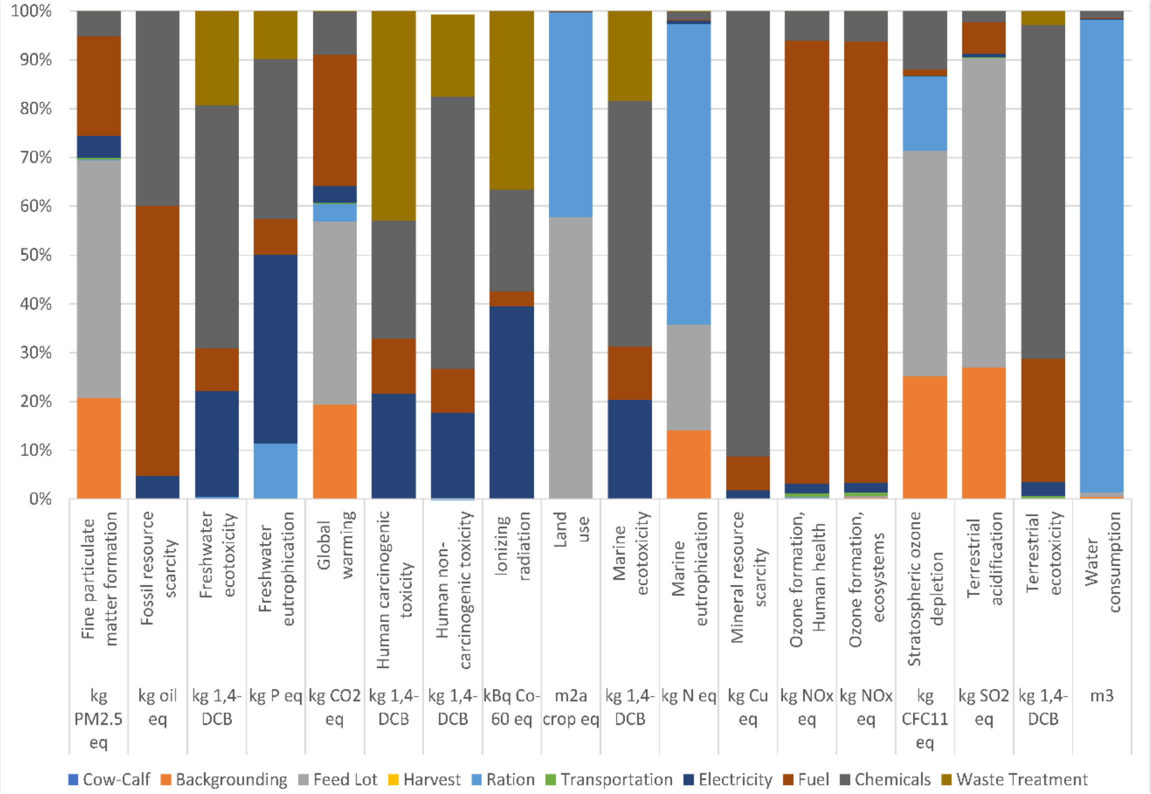

Figure J-9. Scenario 5: Background plus Feed Yard GtG Conventional Corn - paired LWG

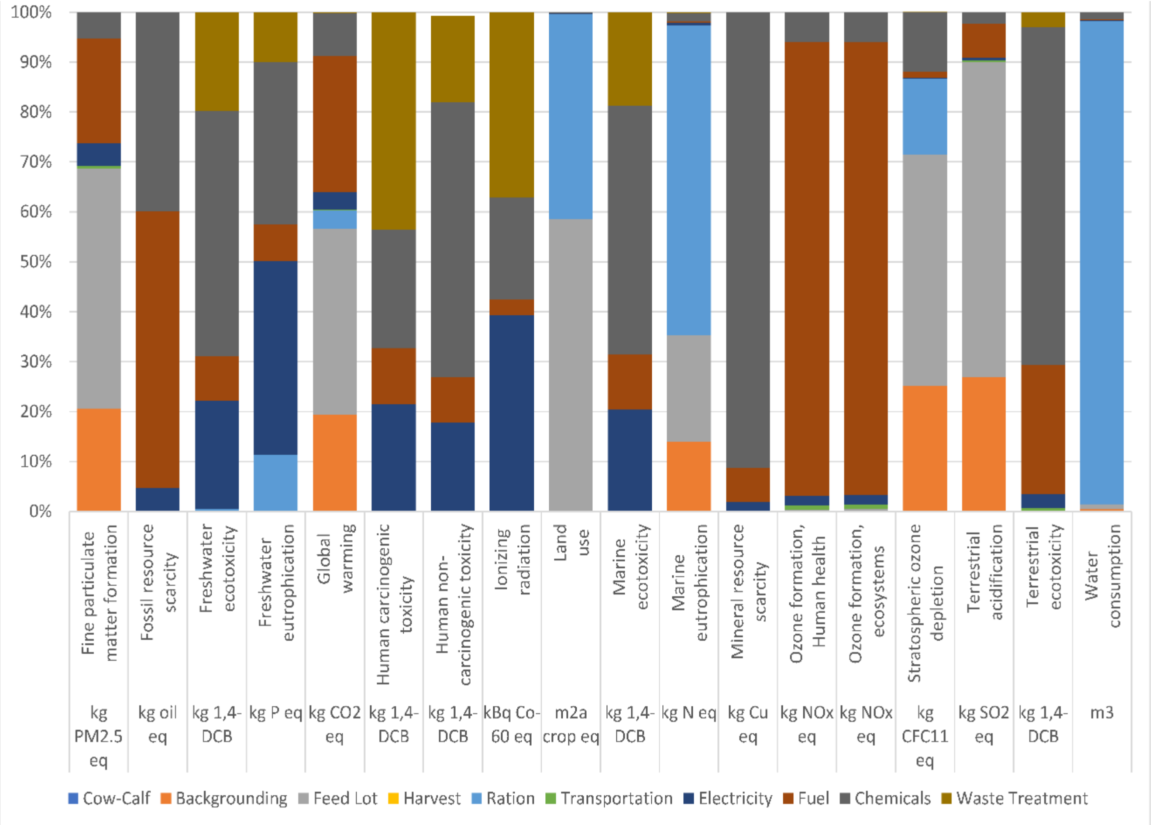

Figure J-10. Scenario 5: Background plus Feed Yard GtG Enogen Feed Corn - paired LWG

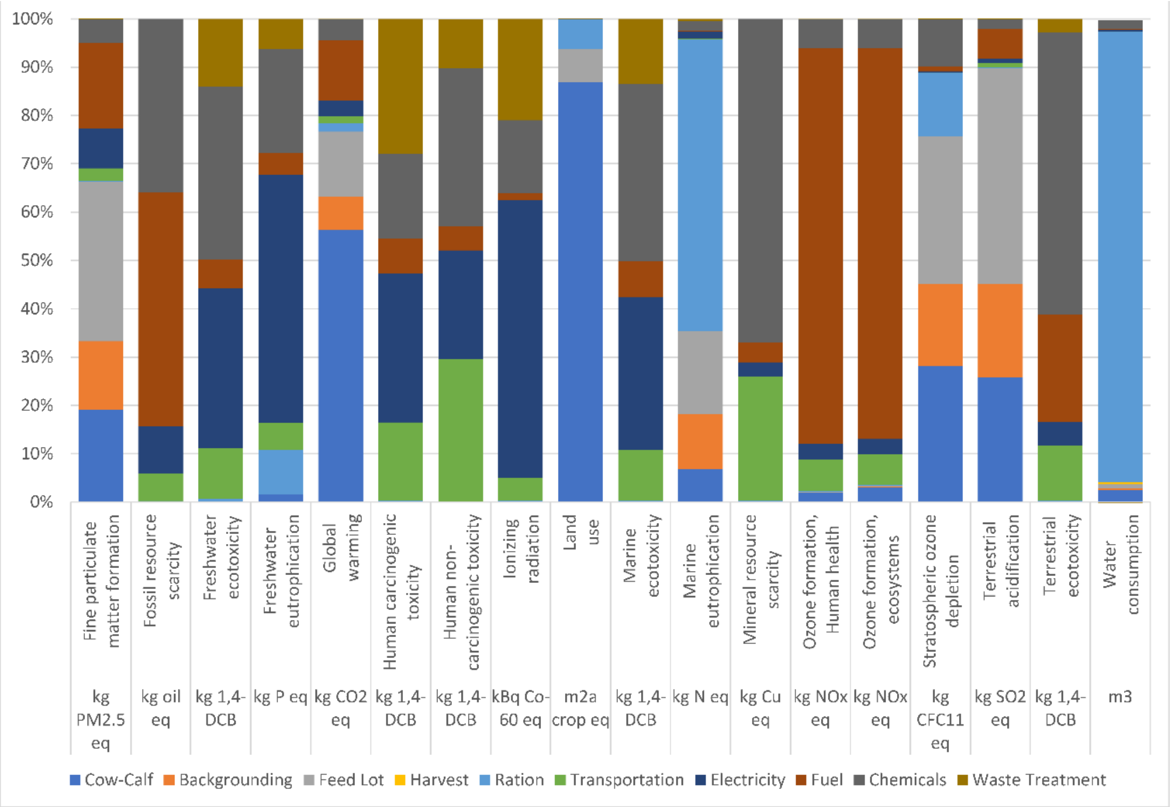

Figure J-11. Scenario 5: Cradle-to-Retail Cut; Conventional Corn - paired LWG

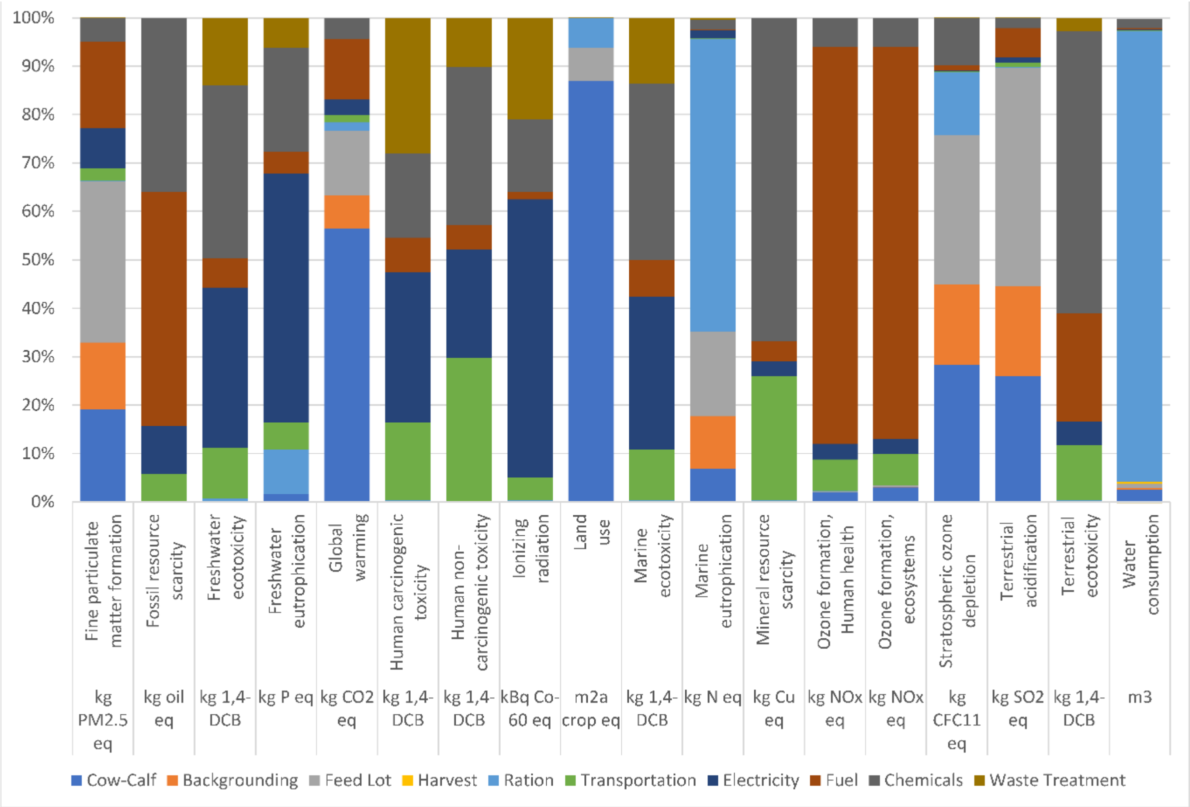

Figure J-12. Scenario 5: Cradle-to-Retail Cut; Enogen Feed Corn - paired LWG

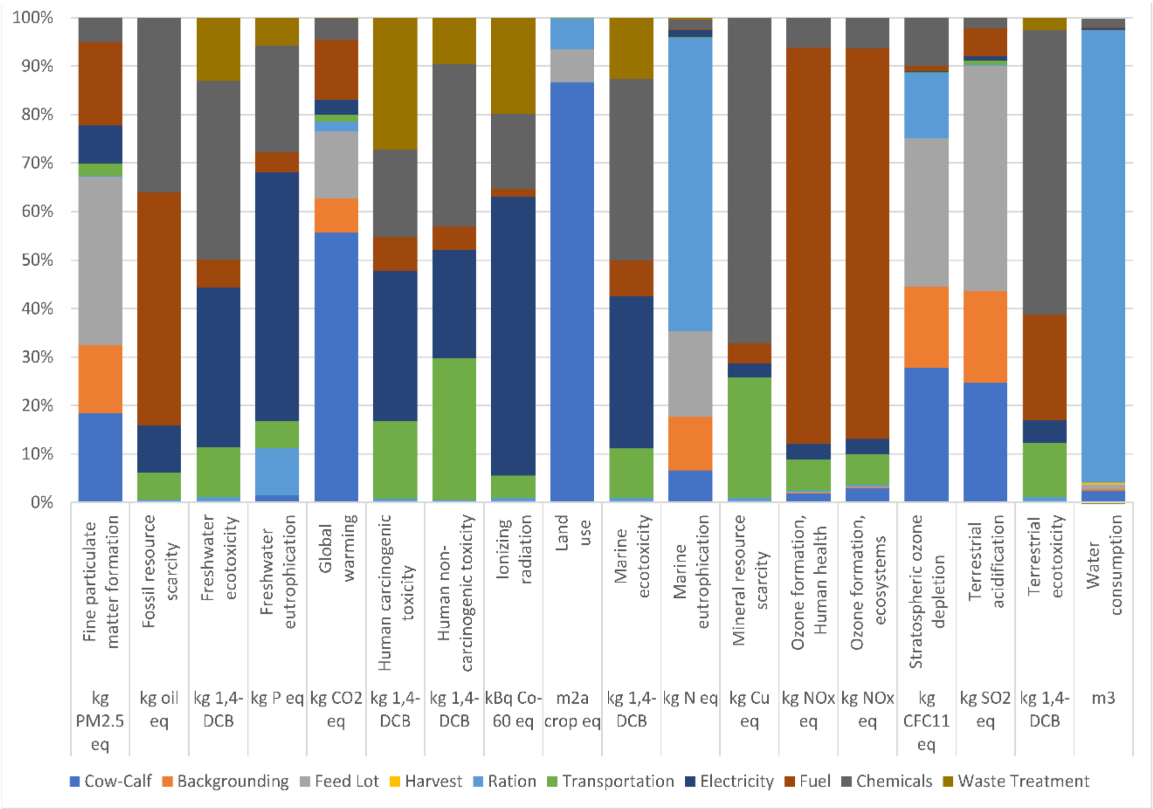

Figure J-13. Scenario 6 Background plus Feed Yard GtG Conventional Corn - matched LWG

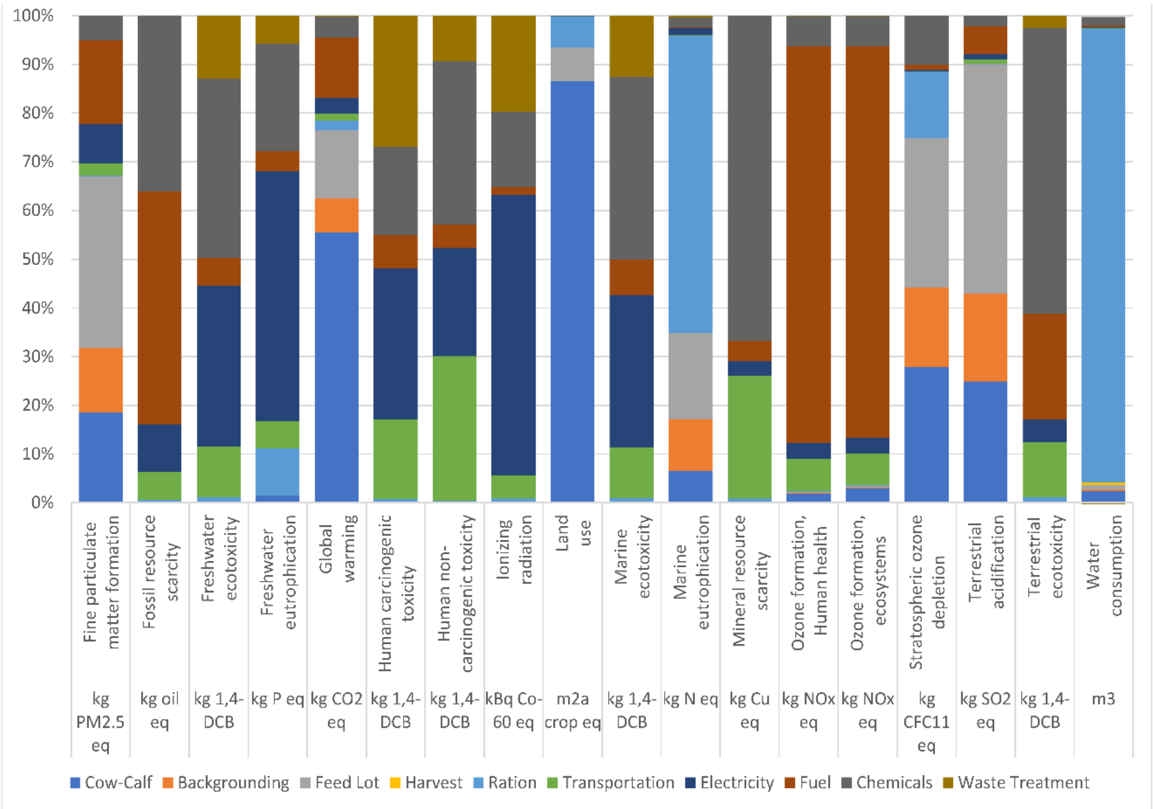

Figure J-14. Scenario 6: Background plus Feed Yard GtG Conventional Corn - matched LWG

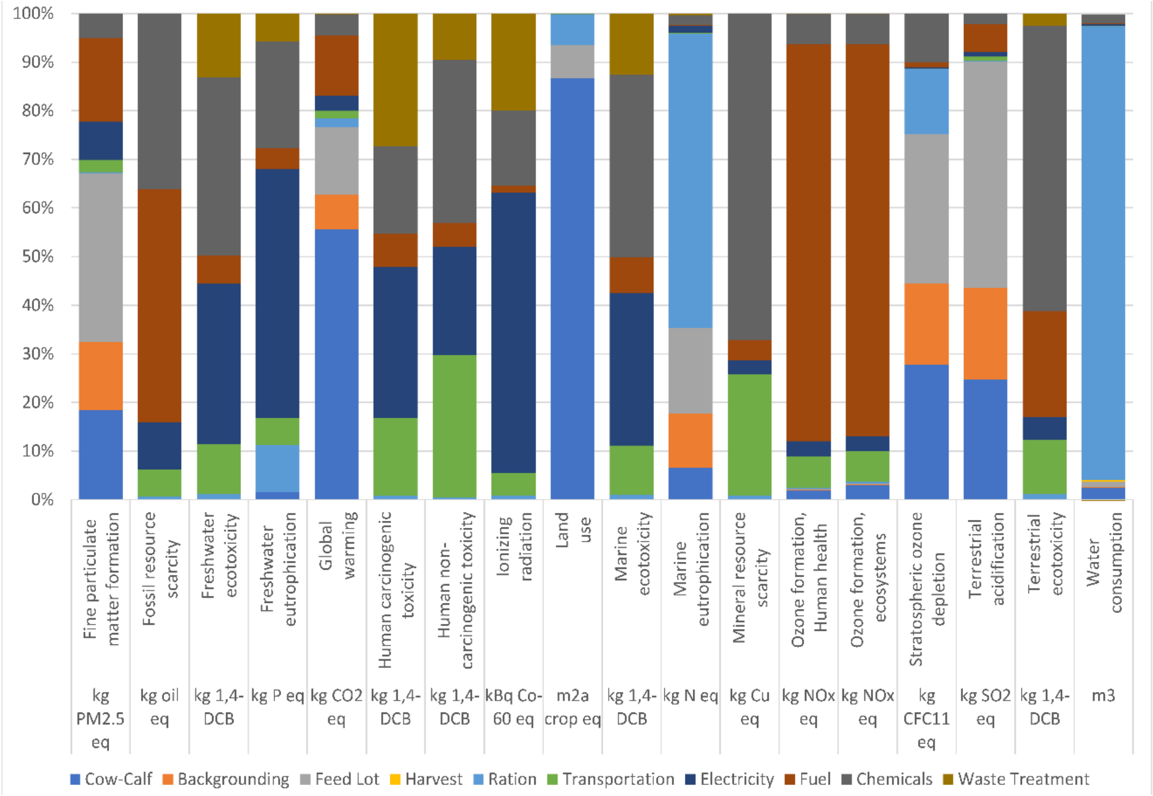

Figure J-15. Scenario 6: Cradle-to-Retail Cut; Conventional Corn - matched LWG

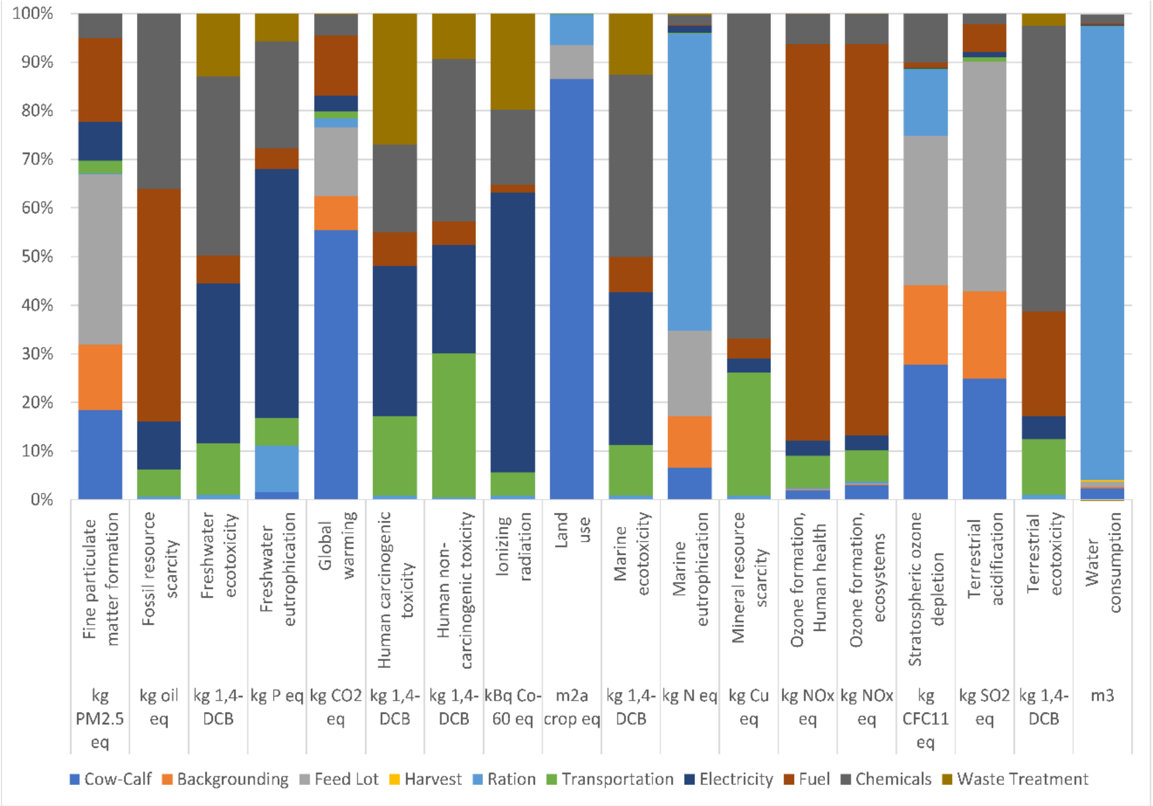

Figure J-16. Scenario 6: Cradle-to-Retail Cut; Enogen Feed Corn - matched LWG

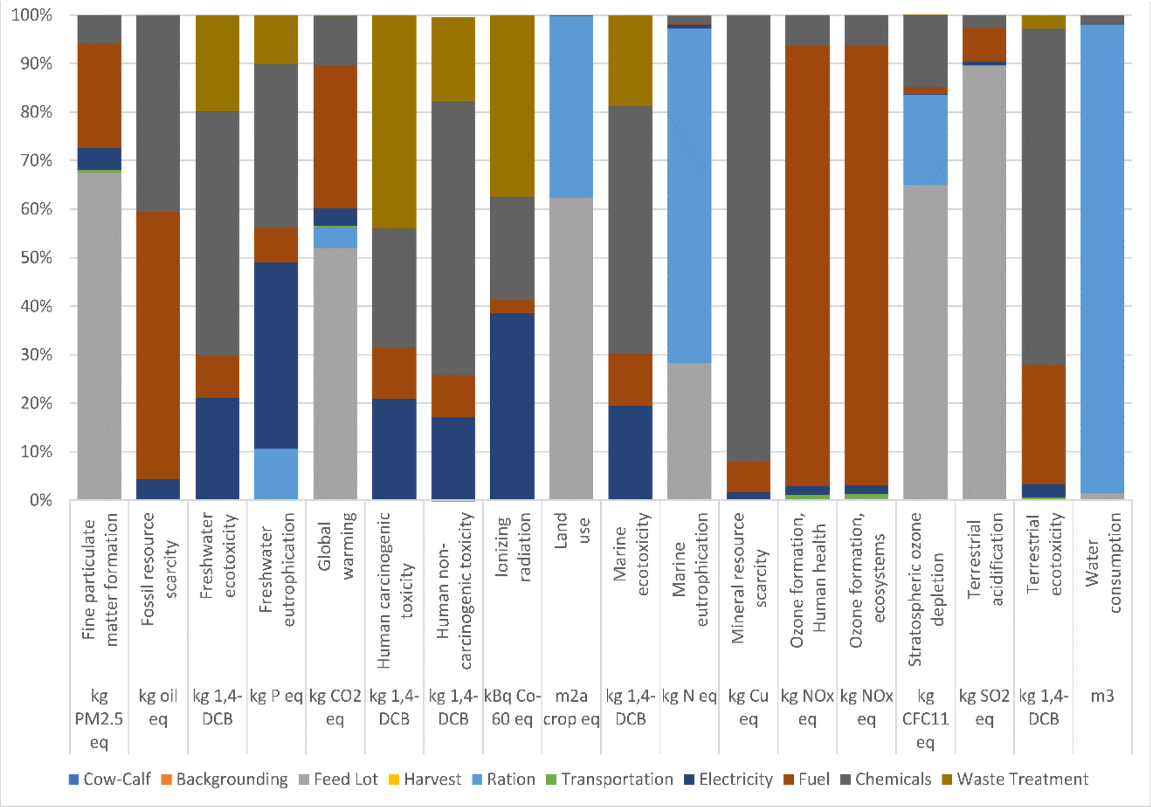

Figure J-17. Scenario 6: Feed Yard, Conventional Corn - matched LWG

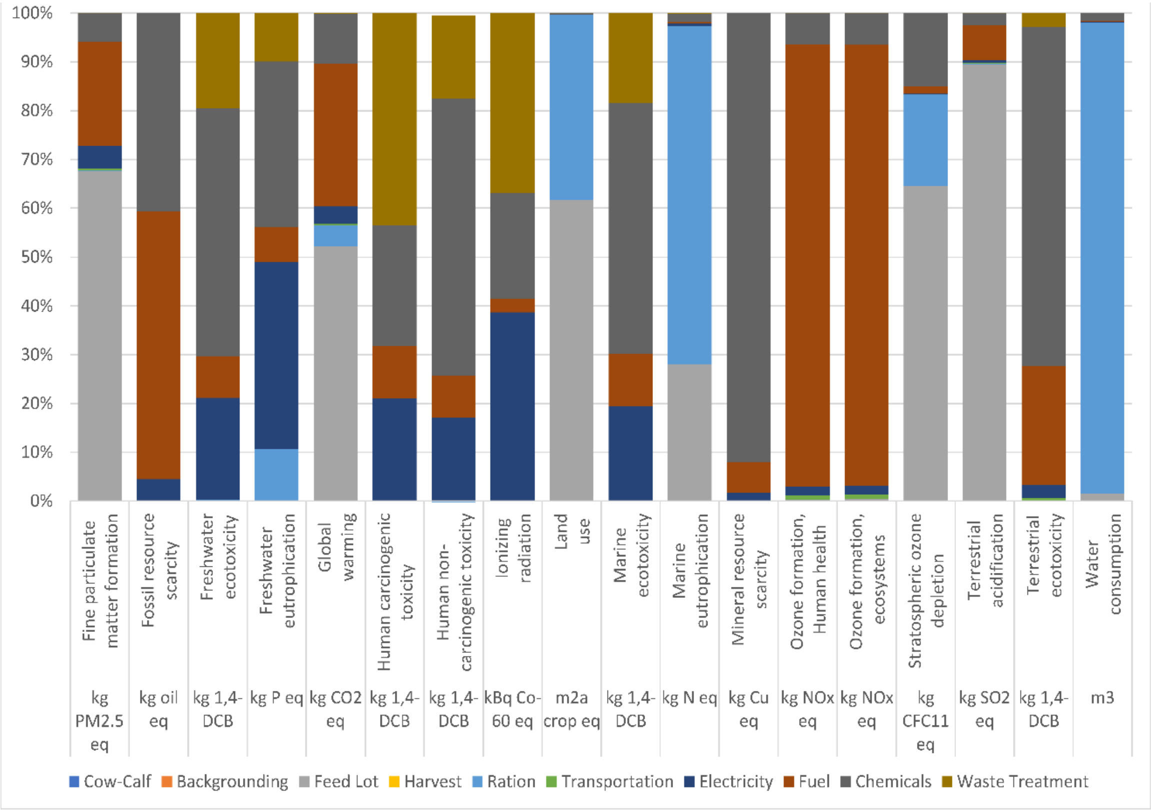

Figure J-18. Scenario 6: Feed Yard, Enogen Feed Corn - matched LWG

Appendix K. Additional LCI datasets

Inventory data for supporting unit processes are provided in the following table.

Alfalfa Hay

Input Flow	Category	Amount	Unit	Uncertainty	Provider	Description
**diesel, burned in building machine**	Ecoinvent_34_cutoff/F:Construction/43:Specialized construction activities/431:Demolition and site preparation/4312:Site preparation	773,156	MJ	normal: mean=773156 sigma=59270.9	diesel, burned in building machine | diesel, burned in building machine | Cutoff, S - GLO	
**Water, groundwater consumption**	Elementary flows/Resource/unspecified	518,835	t	normal: mean=518835 sigma=92191.0		Irrigation water, smr
**potassium chloride, as K2O**	Ecoinvent_34_cutoff/C:Manufacturing/20:Manufacture of chemicals and chemical products/201:Manufacture of basic chemicals, fertilizers and nitrogen compounds, plastics and synthetic rubber in primary forms/2011:Manufacture of basic chemicals	69,666	kg	none	market for potassium chloride, as K2O | potassium chloride, as K2O | Cutoff, S - GLO	
**phosphate fertiliser, as P2O5**	Ecoinvent_34_cutoff/C:Manufacturing/20:Manufacture of chemicals and chemical products/201:Manufacture of basic chemicals, fertilizers and nitrogen compounds, plastics and synthetic rubber in primary forms/2012:Manufacture of fertilizers and nitrogen compounds	14,943	kg	none	triple superphosphate production | phosphate fertiliser, as P2O5 | Cutoff, S - RoW	
**heat, central or small-scale, natural gas**	Ecoinvent_34_cutoff/D:Electricity, gas, steam and air conditioning supply/35:Electricity, gas, steam and air conditioning supply/353:Steam and air conditioning supply/3530:Steam and air conditioning supply	14,534	m3	normal: mean=14534.3 sigma=2425.90	natural gas, burned in micro gas turbine, 100kWe | heat, central or small-scale, natural gas | Cutoff, S - RoW	
**pesticide, unspecified**	Ecoinvent_34_cutoff/C:Manufacturing/20:Manufacture of chemicals and chemical products/202:Manufacture of other chemical products/2021:Manufacture of	400	kg	none	market for pesticide, unspecified | pesticide, unspecified | Cutoff, S - GLO	

	pesticides and other agrochemical products					
**Occupation, pasture, man made**	Elementary flows/Resource/land	200	ha*a	none		HQ alfalfa hay area
**Output Flow**	**Category**	**Amount**	**Unit**	**Uncertainty**	**Provider**	**Description**
**hay; regional average production; at farm; dry matter**	Resilient Food Database/Agriculture, forestry, and fishing/ISIC 0119: Growing of other non-perennial crops	1,851	t	normal: mean=1851.00 sigma=318.000		
**Dinitrogen monoxide**	Elementary flows/Emission to air/low population density	79	kg	normal: mean=79.0392 sigma=0.06600 70		Nitrous oxide from farmland
**Phosphorus**	Elementary flows/Emission to water/fresh water	19.9	kg	normal: mean=19.9000 sigma=16.7000		Phosphorus from sediment runoff
**Phosphorus**	Elementary flows/Emission to water/fresh water	12.6	kg	normal: mean=12.6000 sigma=11.2500		Phosphorus from soluble runoff
**Nitrogen**	Elementary flows/Emission to water/fresh water	6	kg	normal: mean=6.00000 sigma=7.90000		Nitrogen from runoff
**Phosphorus**	Elementary flows/Emission to soil/agricultural	0.1	kg	normal: mean=0.10000 0 sigma=0.23000 0		Phosphorus from leaching

Weaned Calves

Input Flow	Category	Amount	Unit	Uncertainty	Provider	Description
**diesel, burned in building machine**	Ecoinvent_34_cutoff/F:Construction/43:Specialized construction activities/431:Demolition and site preparation/4312:Site preparation	369,635	MJ	normal: mean=369635 sigma=18481.8	diesel, burned in building machine | diesel, burned in building machine | Cutoff, U - GLO	
**electricity, medium voltage**	Ecoinvent_34_cutoff/D:Electricity, gas, steam and air conditioning supply/35:Electricity, gas, steam and air conditioning supply/351:Electric power generation, transmission and distribution/3510:Electric power generation, transmission and distribution	24,283	kWh	normal: mean=24283.0 sigma=1214.15	market for electricity, medium voltage | electricity, medium voltage | Cutoff, S - MRO, US only	
**Water, groundwater consumption**	Elementary flows/Resource/unspecified	2,930	t	normal: mean=2930.00 sigma=293.000		Drinking water, smr
**Occupation, pasture, man made, intensive**	Elementary flows/Resource/land	815	ha*a	none		Grass pasture area
**Occupation, pasture, man made, intensive**	Elementary flows/Resource/land	350	ha*a	none		Small grain grazing area
**pesticide, unspecified**	Ecoinvent_34_cutoff/C:Manufacturing/20:Manufacture of chemicals and chemical products/202:Manufacture of other chemical products/2021:Manufacture of pesticides and other agrochemical products	163	kg	normal: mean=163.000 sigma=8.15000	market for pesticide, unspecified | pesticide, unspecified | Cutoff, S - GLO	

**undegradable protein supplement; regional mix; at regional storage; dry matter**	Resilient Food Database/Manufacturing/ISIC 1080: Manufacture of prepared animal feeds	35.2	t	normal: mean=35.2000 sigma=1.76000	distillers grain production; ethanol production; at regional storage	
**corn grain feed; regional average production; at farm; dry matter**	Resilient Food Database/Agriculture, forestry, and fishing/ISIC 0111: Growing of cereals (except rice), leguminous crops and oil seeds	33.4	t	normal: mean=33.4000 sigma=1.67000	corn grain production; north plains regional average; at farm - US-NPR	
**crude protein supplement; regional mix; at regional storage; dry matter**	Resilient Food Database/Manufacturing/ISIC 1080: Manufacture of prepared animal feeds	16.6	t	normal: mean=16.6000 sigma=0.83000 0	corn gluten meal; crude protein supplement; for cattle; at regional storage - US	
**hay; regional average production; at farm; dry matter**	Resilient Food Database/Agriculture, forestry, and fishing/ISIC 0119: Growing of other non-perennial crops	11.4	t	normal: mean=11.4000 sigma=0.57000 0	alfalfa hay production; north plains regional average; at farm - US-NPR	
**vitamin premix; cow feed vitamin mix; at processing plant gate**	Resilient Food Database/Manufacturing/ISIC 1080: Manufacture of prepared animal feeds	9.6	t	normal: mean=9.60000 sigma=0.48000 0	vitamin premix; cow feed vitamin mix; at processing plant gate - US	

Output Flow	Category	Amount	Unit	Uncertainty	Provider	Description
**weaned calves; CC operation; at farm - US**	Resilient Food Database/Agriculture, forestry, and fishing/ISIC 0141: Raising of cattle and buffaloes	189	Item(s)	none		
**cull cattle; CC operation; cows and bulls, at farm; live weight - US**	Resilient Food Database/Agriculture, forestry, and fishing/ISIC 0141: Raising of cattle and buffaloes	15,571	kg	none		15571 lbs LW from culls and 0 lbs LW from finishers
**Methane, non-fossil**	Elementary flows/Emission to air/unspecified	27,169	kg	normal: mean=27168.6 sigma=2716.86		Methane from animals
**Ammonia**	Elementary flows/Emission to air/low population density	2,098	kg	normal: mean=2097.91 sigma=209.791		Ammonia from grazing
**Methane, non-fossil**	Elementary flows/Emission to air/unspecified	453	kg	normal: mean=453.047 sigma=45.3047		Methane from field application
**NMVOC, non-methane volatile organic compounds, unspecified origin**	Elementary flows/Emission to air/low population density	263	kg	normal: mean=263.159 sigma=26.3159		NMVOC from field and grazing
**Dinitrogen monoxide**	Elementary flows/Emission to air/low population density	169	kg	normal: mean=169.379 sigma=16.9379		Nitrous oxide from farmland
**Nitrogen**	Elementary flows/Emission to soil/agricultural	142	kg	normal: mean=142.125 sigma=14.2125		Nitrogen from leaching

**Dinitrogen monoxide**	Elementary flows/Emission to air/low population density	48.6	kg	normal: mean=48.5608 sigma=4.85608		Nitrous oxide from animals
**Hydrogen sulfide**	Elementary flows/Emission to air/low population density	2.27	kg	normal: mean=2.26855 sigma=0.226855		Hydrogen sulfide from grazing
**Phosphorus**	Elementary flows/Emission to water/fresh water	1.1	kg	normal: mean=1.10000 sigma=0.110000		Phosphorus from soluble runoff
**Phosphorus**	Elementary flows/Emission to water/fresh water	0.7	kg	normal: mean=0.700000 sigma=0.0700000		Phosphorus from sediment runoff
**Phosphorus**	Elementary flows/Emission to soil/agricultural	0.5	kg	normal: mean=0.500000 sigma=0.0500000		Phosphorus from leaching
**Dinitrogen monoxide**	Elementary flows/Emission to air/low population density	0.04	kg	normal: mean=0.0400000 sigma=0.00400000		Nitrous oxide from indirect sources

Corn Grain

Input Flow	Category	Amount	Unit	Uncertainty	Provider	Description
**Water, groundwater consumption**	Elementary flows/Resource/unspecified	1,217,515	t	normal: mean=1.21752 E+06 sigma=318931		Irrigation water, smr

**diesel, burned in building machine**	Ecoinvent_34_cutoff/F:Construction/43:Specialized construction activities/431:Demolition and site preparation/4312:Site preparation	666,844	MJ	normal: mean=666844 sigma=77582.2	diesel, burned in building machine | diesel, burned in building machine | Cutoff, S - GLO	
**electricity, medium voltage**	Ecoinvent_34_cutoff/D:Electricity, gas, steam and air conditioning supply/35:Electricity, gas, steam and air conditioning supply/351:Electric power generation, transmission and distribution/3510:Electric power generation, transmission and distribution	161,895	kWh	normal: mean=161895 sigma=23983.0	market for electricity, medium voltage | electricity, medium voltage | Cutoff, S - MRO, US only	
**nitrogen fertiliser, as N**	Ecoinvent_34_cutoff/C:Manufacturing/20:Manufacture of chemicals and chemical products/201:Manufacture of basic chemicals, fertilizers and nitrogen compounds, plastics and synthetic rubber in primary forms/2012:Manufacture of fertilizers and nitrogen compounds	89,670	kg	none	urea ammonium nitrate production | nitrogen fertiliser, as N | Cutoff, S - RoW	
**heat, central or small-scale, natural gas**	Ecoinvent_34_cutoff/D:Electricity, gas, steam and air conditioning supply/35:Electricity, gas, steam and air conditioning supply/353:Steam and air conditioning supply/3530:Steam and air conditioning supply	62,247	m3	normal: mean=62247.4 sigma=102802	natural gas, burned in micro gas turbine, 100kWe | heat, central or small-scale, natural gas | Cutoff, S - RoW	
**potassium chloride, as K2O**	Ecoinvent_34_cutoff/C:Manufacturing/20:Manufacture of chemicals and chemical products/201:Manufacture of basic chemicals, fertilizers and nitrogen compounds, plastics and synthetic rubber in primary forms/2011:Manufacture of basic chemicals	50,633	kg	none	market for potassium chloride, as K2O | potassium chloride, as K2O | Cutoff, S - GLO	

**phosphate fertiliser, as P2O5**	Ecoinvent_34_cutoff/C:Manufacturing/20:Manufacture of chemicals and chemical products/201:Manufacture of basic chemicals, fertilizers and nitrogen compounds, plastics and synthetic rubber in primary forms/2012:Manufacture of fertilizers and nitrogen compounds	50,250	kg	none	triple superphosphate production | phosphate fertiliser, as P2O5 | Cutoff, S - RoW	
**pesticide, unspecified**	Ecoinvent_34_cutoff/C:Manufacturing/20:Manufacture of chemicals and chemical products/202:Manufacture of other chemical products/2021:Manufacture of pesticides and other agrochemical products	2,000	kg	none	market for pesticide, unspecified | pesticide, unspecified | Cutoff, S - GLO	
**Occupation, annual crop, intensive**	Elementary flows/Resource/land	500	ha*a	none		Corn grain area
**lime**	Ecoinvent_34_cutoff/B:Mining and quarrying/08:Other mining and quarrying/081:Quarrying of stone, sand and clay/0810:Quarrying of stone, sand and clay	290	t	none	lime production, milled, loose | lime | Cutoff, S - CA-QC	

**Output Flow**	**Category**	**Amount**	**Unit**	**Uncertainty**	**Provider**	**Description**
**corn grain feed; regional average production; at farm; dry matter**	Resilient Food Database/Agriculture, forestry, and fishing/ISIC 0111: Growing of cereals (except rice), leguminous crops and oil seeds	4,617	t	normal: mean=4617.00 sigma=793.000		
**Nitrogen**	Elementary flows/Emission to soil/agricultural	14,251	kg	normal: mean=14251.0 sigma=11937.8		Nitrogen from leaching

**Dinitrogen monoxide**	Elementary flows/Emission to air/low population density	983	kg	normal: mean=982.543 sigma=0.177729		Nitrous oxide from farmland
**Nitrogen**	Elementary flows/Emission to water/fresh water	244	kg	normal: mean=244.000 sigma=346.800		Nitrogen from runoff
**Dinitrogen monoxide**	Elementary flows/Emission to air/low population density	127	kg	normal: mean=127.400 sigma=0.355641		Nitrous oxide from indirect sources
**Phosphorus**	Elementary flows/Emission to water/fresh water	62.2	kg	normal: mean=62.2000 sigma=85.9500		Phosphorus from sediment runoff
**Phosphorus**	Elementary flows/Emission to soil/agricultural	10.8	kg	normal: mean=10.8000 sigma=7.09000		Phosphorus from leaching
**Phosphorus**	Elementary flows/Emission to water/fresh water	7.6	kg	normal: mean=7.60000 sigma=9.84000		Phosphorus from soluble runoff

**Corn Silage**						
**Input Flow**	**Category**	**Amount**	**Unit**	**Uncertainty**	**Provider**	**Description**
**diesel, burned in building machine**	Ecoinvent_34_cutoff/F:Construction/43:Specialized construction activities/431:Demolition and site preparation/4312:Site preparation	666,844	MJ	normal: mean=666844 sigma=77582.2	diesel, burned in building machine | diesel, burned in building machine | Cutoff, S - GLO	
**electricity, medium voltage**	Ecoinvent_34_cutoff/D:Electricity, gas, steam and air conditioning supply/35:Electricity, gas, steam and air conditioning supply/351:Electric power generation, transmission and	161,895	kWh	normal: mean=161895 sigma=23983.0	market for electricity, medium voltage | electricity, medium voltage | Cutoff, S - MRO, US only	

	distribution/3510:Electric power generation, transmission and distribution					
**heat, central or small-scale, natural gas**	Ecoinvent_34_cutoff/D:Electricity, gas, steam and air conditioning supply/35:Electricity, gas, steam and air conditioning supply/353:Steam and air conditioning supply/3530:Steam and air conditioning supply	62,247	m3	normal: mean=62247.4 sigma=102802	natural gas, burned in micro gas turbine, 100kWe | heat, central or small-scale, natural gas | Cutoff, S - RoW	
**lime**	Ecoinvent_34_cutoff/B:Mining and quarrying/08:Other mining and quarrying/081:Quarrying of stone, sand and clay/0810:Quarrying of stone, sand and clay	290	t	none	lime production, milled, loose | lime | Cutoff, S - CA-QC	
**nitrogen fertiliser, as N**	Ecoinvent_34_cutoff/C:Manufacturing/20:Manufacture of chemicals and chemical products/201:Manufacture of basic chemicals, fertilizers and nitrogen compounds, plastics and synthetic rubber in primary forms/2012:Manufacture of fertilizers and nitrogen compounds	89,670	kg	none	urea ammonium nitrate production | nitrogen fertiliser, as N | Cutoff, S - RoW	
**Occupation, annual crop, intensive**	Elementary flows/Resource/land	500	ha*a	none		Corn grain area
**pesticide, unspecified**	Ecoinvent_34_cutoff/C:Manufacturing/20:Manufacture of chemicals and chemical products/202:Manufacture of other chemical products/2021:Manufacture of pesticides and other agrochemical products	2,000	kg	none	market for pesticide, unspecified | pesticide, unspecified | Cutoff, S - GLO	

**phosphate fertiliser, as P2O5**	Ecoinvent_34_cutoff/C:Manufacturing/20:Manufacture of chemicals and chemical products/201:Manufacture of basic chemicals, fertilizers and nitrogen compounds, plastics and synthetic rubber in primary forms/2012:Manufacture of fertilizers and nitrogen compounds	50,250	kg	none	triple superphosphate production | phosphate fertiliser, as P2O5 | Cutoff, S - RoW	
**potassium chloride, as K2O**	Ecoinvent_34_cutoff/C:Manufacturing/20:Manufacture of chemicals and chemical products/201:Manufacture of basic chemicals, fertilizers and nitrogen compounds, plastics and synthetic rubber in primary forms/2011:Manufacture of basic chemicals	50,633	kg	none	market for potassium chloride, as K2O | potassium chloride, as K2O | Cutoff, S - GLO	
**Water, groundwater consumption**	Elementary flows/Resource/unspecified	1,217,515	t	normal: mean=1.21752 E+06 sigma=318931		Irrigation water, smr

**Output Flow**	**Category**	**Amount**	**Unit**	**Uncertainty**	**Provider**	**Description**
**grain silage; regional average production; at farm; dry matter**	Resilient Food Database/Agriculture, forestry, and fishing/ISIC 0111: Growing of cereals (except rice), leguminous crops and oil seeds	4,617	t	normal: mean=4617.00 sigma=793.000		
**Dinitrogen monoxide**	Elementary flows/Emission to air/low population density	983	kg	normal: mean=982.543 sigma=0.17773		Nitrous oxide from farmland
**Dinitrogen monoxide**	Elementary flows/Emission to air/low population density	127	kg	normal: mean=127.400 sigma=0.35564		Nitrous oxide from indirect sources

**Nitrogen**	Elementary flows/Emission to soil/agricultural	14,251	kg	normal: mean=14251.0 sigma=11937.8		Nitrogen from leaching
**Nitrogen**	Elementary flows/Emission to water/fresh water	244	kg	normal: mean=244.000 sigma=346.800		Nitrogen from runoff
**Phosphorus**	Elementary flows/Emission to soil/agricultural	10.8	kg	normal: mean=10.8000 sigma=7.09000		Phosphorus from leaching
**Phosphorus**	Elementary flows/Emission to water/fresh water	7.6	kg	normal: mean=7.60000 sigma=9.84000		Phosphorus from soluble runoff
**Phosphorus**	Elementary flows/Emission to water/fresh water	62.2	kg	normal: mean=62.2000 sigma=85.9500		Phosphorus from sediment runoff
